# Multimaterial fiber as a physical simulator of a capillary instability

**DOI:** 10.1038/s41467-023-41216-7

**Published:** 2023-09-26

**Authors:** Camila Faccini de Lima, Fan Wang, Troy A. Leffel, Tyson Miller, Steven G. Johnson, Alexander Gumennik

**Affiliations:** 1 Department of Intelligent Systems Engineering, Luddy School of Informatics, Computing, and Engineering, Indiana University Bloomington, Bloomington, IN USA; 2 Department of Mechanical Engineering, Massachusetts Institute of Technology, Cambridge, MA USA; 3 Department of Mathematics, Massachusetts Institute of Technology, Cambridge, MA USA; 4 Department of Physics, Massachusetts Institute of Technology, Cambridge, MA USA

**Keywords:** Electronic devices, Fluid dynamics, Applied mathematics, Optical materials and structures, Sensors

## Abstract

Capillary breakup of cores is an exclusive approach to fabricating fiber-integrated optoelectronics and photonics. A physical understanding of this fluid-dynamic process is necessary for yielding the desired solid-state fiber-embedded multimaterial architectures by design rather than by exploratory search. We discover that the nonlinearly complex and, at times, even chaotic capillary breakup of multimaterial fiber cores becomes predictable when the fiber is exposed to the spatiotemporal temperature profile, imposing a viscosity modulation comparable to the breakup wavelength. The profile acts as a notch filter, allowing only a single wavelength out of the continuous spectrum to develop predictably, following Euler-Lagrange dynamics. We argue that this understanding not only enables designing the outcomes of the breakup necessary for turning it into a technology for materializing fiber-embedded functional systems but also positions a multimaterial fiber as a universal physical simulator of capillary instability in viscous threads.

## Abstract (web summary)

Capillary breakup in multimaterial fibers is explored for the self-assembly of optoelectronic systems. However, its insights primarily stem from numerical simulations, qualitative at best. The authors formulate an analytical model of such breakup, obtaining a window in the governing parameters where the generally chaotic breakup becomes predictable and thus engineerable.

## Introduction

Monofilament fibers resulting from a thermal draw^1,2^ are a unique platform for implementing pervasive, functional devices and systems, from traditional fiber optics for telecommunication through fibers-devices for signal transduction^3–9^ and energy management^10,11^ to fibers with computational and data processing capabilities^12,13^.

**Fig. 1:**
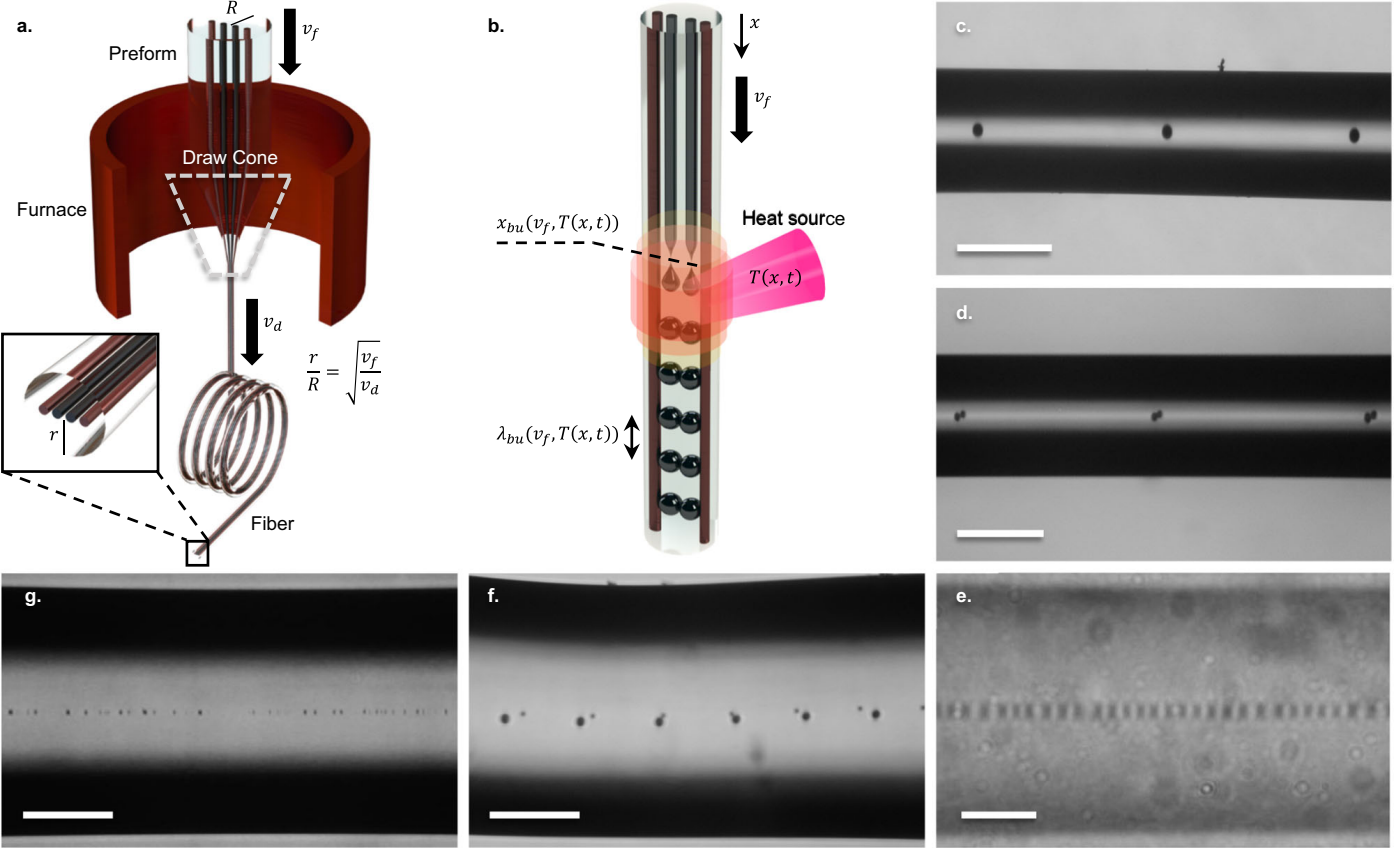
Liquid phase fiber manufacturing and post-processing. **a** Thermal Draw. Notations: *R* - preform radius, *r* - fiber radius, *R/r* - draw scaling factor, *v*_*f*_ - feed speed, *v*_*d*_ - draw speed. **b** System assembly by a spatially coherent material-selective capillary breakup in an externally induced time-varying temperature gradient. The two inner cores of the fiber are, for instance, p- and n-doped silicon, while the two outer cores are high-temperature metal, such as vanadium or platinum, which is liquid during the fiber draw at 2000 °C but stays solid during the capillary breakup process, performed in a window between the softening point of silica (~1650 °C) and melting point of metal (1768 °C for platinum, 1910 °C for vanadium). In that case, the process will result in an array of bi-spherical pn Si diodes contacted in parallel by metallic electrodes. Notations: *x*_*bu*_ - breakup location, *λ*_*bu*_ - breakup period, *T(x,t)* - spatiotemporal temperature profile. **c**–**g** Breakup in Si-core single- and dual-core fibers. **c** Regular breakup in 4 µm thick core (scale bar 200 µm). **d** Coherent breakup in dual-core fiber with similar cores 2 µm thick (scale bar 200 µm). **e** Regular breakup in 0.35 µm thick core (scale bar 5 µm, the spheres don’t look spherical due to aberrations resulting from cylindrical fiber cladding). **f** In-and-out of phase breakup in a dual-core fiber with dissimilar cores, 2 µm and 0.7 µm thick (scale bar 100 µm). **g** Irregular breakup in 0.5 µm thick core (scale bar 100 µm).

Fiber fabrication and post-processing combine pulling and shaping the cladding and materials it encapsulates in a molten, viscous state (Fig. 1a). Essentially, it is a reflow of a viscous liquid, and as such, it is governed by nonlinear, often chaotic^14–16^ fluid dynamics. Nonetheless, it is required to deliver a predictable, ordered solid-state architecture, defining the functionality of the fiber-embedded device or system.

For instance, capillary instability is increasingly used to pattern the fiber (Fig. 1b) along its axial, otherwise uniform out-of-the-draw, dimension. Either promoted globally by heating the entire fiber to a state of viscous liquid^8,17^ or locally by gradually feeding the fiber through a delineated liquefaction zone^7,18,19^, the capillary instability often bears pronounced characteristics of chaos. In the former case, the breakup is polydisperse. The main breakup period fluctuates quite pronouncedly due to any material-morphology imperfection and is often accompanied by secondary breakups, known as satellites^8,9,17^, analogous to bifurcations^14,20,21^, accompanying the onset of chaos. In the latter case, the breakup resembles a dripping faucet, which is in itself a standard chaos demonstrator^14,21,22^.

Other than ab-initio numerical simulations of Navier-Stokes equations, the mathematical approaches providing the physical intuition to the capillary instability dynamics in fibers typically fall into two categories. On the one hand, in a globally liquified fiber, the standard description is in terms of linear stability analysis, as proposed by Tomotika^23^, where out of the continuous spectrum of all the possible instability wavelengths developing concurrently within an infinite viscous thread, one, called dominant, has the fastest development rate, and defines the eventual breakup period. On the other hand, the situation in the locally liquified fiber is better described by the Plateau-Rayleigh instability propagating along the semi-infinite viscous thread beginning its tip^24^, according to the dynamics defined by the marginal stability criterion^25^. Here, the dominant wavelength is the one that has the fastest rate of propagation. Both models yield a single predictable breakup period, while we know from experiments that the real-world outcomes are far more complex.

Despite obscure physical intuition about the process, the capillary breakup is already extensively explored for the assembly of photonic devices and systems, including p-n diodes^7,26–28^, heterojunctions^6^, arrayed photodetectors^9^, photonic gratings^7^, and optical resonators^18,29^. This is achieved under conditions of localized liquefaction by feeding the fiber through a hot zone of a hydrogen-oxygen flame or a high-intensity laser beam (Fig. 1b). It is expected^7^ that the feed rate of the fiber through the liquefaction zone defines the breakup period, while the boundary of the liquefaction zone defines the pinch off-location of the droplets breaking off the cores. Starting from initially separate cores, multi-spherical devices contacted in parallel can be assembled (Fig. 1b, d) when the breakup is spatially coherent and monodisperse (Fig. 1c–e). In our experience, however, in addition to some selected conditions where this is possible, in a general case, the breakup often is characterized by an incontrollable phase (Fig. 1f) or period (Fig. 1g).

Recent extensive experimental, numerical, and analytical studies^6,19,30–33^ provide significant insight into the behavior of capillary breakup. Notably, Mowlavi et al.^30^ and Shukla et al.^31^ show successful implementations of numerical methods for simulating capillary breakup. The development of a mathematical model built upon the valuable acumen accumulated so far and capable of translating input conditions, such as the spatiotemporal viscosity profile, into breakup outcomes, such as the phase and period, in a one-to-one, quantitatively accurate fashion, yielding a physical intuition of the process is imperative to turning the molten-phase in-fiber device assembly into an engineerable technology.

Here, we interpret the governing mechanisms underlying the breakup in fibers exposed to Axial Viscosity Gradient (AVG), as in localized liquefaction conditions described above, in terms of intuitive first principles, such as balance of forces and energy/momentum conservation laws, culminating in a mathematical model of the process, reduced to Euler-Lagrange dynamics. Subsequently, we demonstrate that the physical intuition provided by the model enables steering the breakup towards predictable, spatially coherent, monodisperse outcomes by design rather than exploration.

## Results

### Physical formulation and mathematical derivation of the axial viscosity gradient instability model (AVG-IM)

In the AVG breakup, the fiber reliquefies locally, and capillary instability breaks up its initially continuous cylindrical cores into arrays of discrete spheres. Since a cylinder’s surface is larger than that of a sphere for the same volume of material, capillary instability is a process of surface energy minimization. Conventional mathematical descriptions of this process^23,24,34^ consider cylindrical liquid threads with axially uniform viscosity in which the instabilities develop at an accelerating rate from the initial white noise simultaneously at all possible spatial frequencies. Such competition between instability wavelengths translated to experimental conditions yields a breakup characterized by probabilistic rather than deterministic outcomes^8,17,35^. Conversely, we observe that in AVG conditions, in which the fiber viscosity changes axially following the temperature profile of the heating source, a specific wavelength out of the continuous spectrum can be amplified selectively, yielding a fully predictable breakup period.

**Fig. 2:**
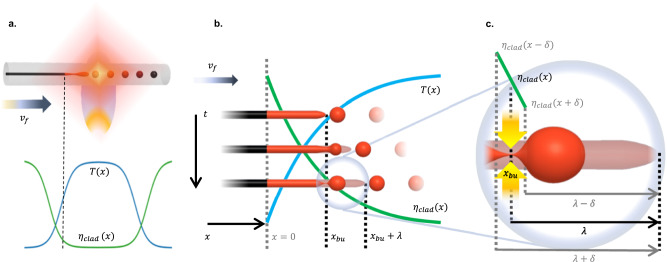
Schematic illustration of capillary breakup in the Axial Viscosity Gradient. **a** When the fiber is fed through the hot zone of a flame or laser, it is exposed to a temperature gradient T(x), which in turn produces a gradient in the fiber’s cladding viscosity, ${\eta }_{{clad}}$. **b** As the fiber is fed through the flame, the temperature gradient induces capillary breakup of the core, which occurs at a fixed position ${x}_{{bu}}$ in regular intervals with period *λ*, depending on the feed speed ${v}_{f}$. **c** Detail on pinch-off position ${x}_{{bu}}$ and neck formation process. For small enough $\delta$, the cladding viscosity may be approximated as a linear function. The core section reshaping into a growing droplet of melt at the core tip is overlayed over a fainted image of the original core, how it would look at a given time if it wouldn’t reshape. It demonstrates the time-lapse of the growing core-section length streamed into the growing droplet as the capillary instability develops between the pinch events.

Based on experimental observation, we consider such an ordered breakup to be a steady-state process where a semi-infinite core of a fiber is fed into the flame at a speed ${v}_{f}$ (Fig. 2a). The fiber geometry remains roughly unchanged before reaching some axial position ${x}_{{bu}}$, at which the core material starts reshaping to form a droplet (Fig. 2b), growing to a critical size and pinching off the core at ${x}_{{bu}}$, as is schematically demonstrated in Fig. 2c. The remainder of the pinching neck then retracts towards the detached droplet and the continuous core, and the process repeats.

**Fig. 3:**
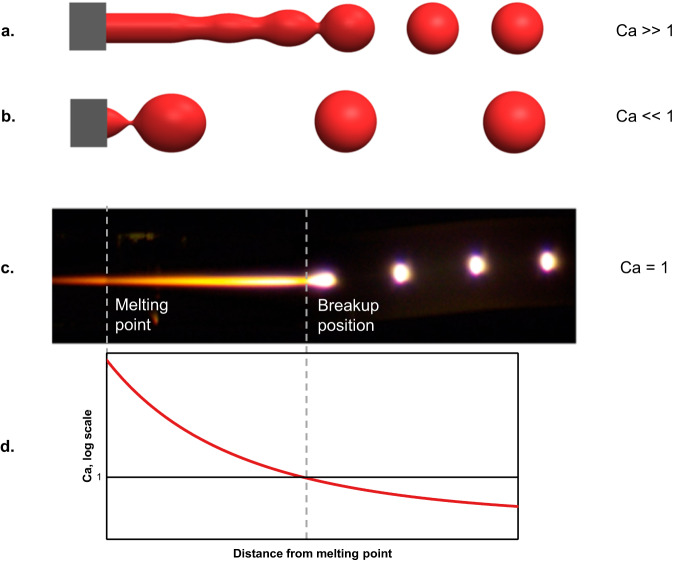
Effective capillary number regimens. **a** Jetting Regime characterized by the formation of a fluid jet and pinch-off away from the orifice (Ca >>1). **b** Dripping Regime characterized by pinch off immediately after the orifice (Ca <<1). **c** Snapshot of AVG Capillary breakup experiment illustrating the formation of a jet and pinch-off at position ${x}_{{bu}}$. **d** Effective capillary number as a function of position: In AVG breakup, the fluid thread switches from jetting to dripping regimens at the breakup location, characterized by Ca=1.

One commonly used criterion for evaluating the breakup of a liquid thread ejected from an orifice, a process closely resembling the description above, is the Effective Capillary Number^35^ (Ca), as it represents the relative effects of viscous and surface tension forces, which determine the breakup behavior. Qualitatively, if $${{{{{\rm{Ca}}}}}} > 1,$$ the system is in a jetting regime since the inner fluid jet extends past the orifice to a length larger than the smallest possible droplet size in the time needed for it to pinch (Fig. 3a). In the case of Ca<1, the system is in the dripping regime, and the pinching will occur immediately after the orifice^35^ (Fig. 3b). We define $${{{{{\rm{Ca}}}}}}={t}_{{pinch}}/{t}_{g}$$ where $${{{{{t}}}}}_{{{{{pinch}}}}}$$ is the droplet pinch time, and $${{{{{t}}}}}_{{{{{g}}}}}$$ is the growth time of the fluid thread before pinching happens. In multimaterial fibers, a core of radius $$a$$ is embedded in a cladding of viscosity $${{{{{\eta }}}}}_{{{{{clad}}}}}$$, with surface tension $${{{{\gamma }}}}$$ between the materials. For the case where the cladding is orders of magnitude more viscous than the core, as is the case with a silica fiber with a silicon core, the pinching time is defined as $${t}_{{pinch}}=\frac{\beta a{\eta }_{{clad}}}{\gamma }$$. *β* is a function of the viscosity ratio between the cladding and core, which asymptotically converges to a constant towards very high viscosity ratios^24^, as for our case. The breakup wavelength is defined as the product between the droplet growth time at the breakup location $${x}_{{bu}}$$$$,$$ and the axial velocity $${{{{{v}}}}}_{{{{{f}}}}}$$ of the core: $$\lambda \equiv {t}_{g}({x}_{{bu}})\cdot{v}_{f}$$. Thus, we can express the Effective Capillary Number as

1 $${{{{{\rm{Ca}}}}}}=\frac{{t}_{{pinch}}}{{t}_{g}}=\frac{\beta a\,{\eta }_{{clad}}{v}_{f}}{\gamma \lambda }$$

Experimentally, the predictable AVG breakup is a steady-state process, in which every droplet pinches off with reaching the critical size at the same axial location ${x}_{{bu}}$. This can be formulated simply as ${t}_{{pinch}}={t}_{g}$, or ${{{{{\rm{Ca}}}}}}=1$ at ${x}_{{bu}}$ (Fig. 3c, d).

One interesting conclusion that stems from the capillary number analysis is that AVG breakup cannot be thought of in common terms of either dripping or jetting, but rather starts as a jetting process and switches to dripping, the deeper into the hot zone the core is fed, while the pinch happens at the regime-switching position, ${{{{{\rm{Ca}}}}}}=1$.

We are looking for a predictable breakup with a well-defined period $\lambda$. Yet, obtaining such $\lambda$ in AVG conditions is not only a matter of synchronizing the droplet growth and its pinch off. Since ${{{{{\eta }}}}}_{{{{{clad}}}}}$ varies in space, Eq. (1), ${{{{{\rm{Ca}}}}}}=\frac{{t}_{{pinch}}}{{t}_{g}}=\frac{\beta a\,{\eta }_{{clad}}{v}_{f}}{\gamma \lambda }=1$, the only equation we have so far to describe our process, becomes a single equation of two unknowns, ${\eta }_{{clad}}$ and $\lambda$, making ${x}_{{bu}}$ indefinable. We suggest that the second independent equation stems from the overlooked fact that the breakup location doesn’t just exist but is also unique. At ${x}_{{bu}}$, the instability doesn’t just synchronize the droplet’s pinch-off with its growth, but completes this synchronization earlier than at any other location. In other words, not just that ${\left.\frac{{t}_{{pinch}}}{{t}_{g}}\right|}_{{x=x}_{{bu}}}=1$, but also ${\left.\frac{{t}_{{pinch}}}{{t}_{g}}\right|}_{{x\ne x}_{{bu}}} > \,1$. I.e., ${{{{{\rm{Ca}}}}}}=\frac{{t}_{{pinch}}}{{t}_{g}}$ assumes a minimum at ${x}_{{bu}}$, or, formally speaking, ${\left.\left(\frac{d}{{dx}}{{{{{\rm{Ca}}}}}}(\lambda,x)\right)\right|}_{{x}_{{bu}}}=0$.

Here, it is critical to note that the uniqueness of the breakup position means that ${\left.x\right|}_{{x}_{{bu}}}$ is not independent of ${\left.\lambda \right|}_{{x}_{{bu}}}$: If there was a different pinch-off at ${\left.(x+{{{{\delta }}}})\right|}_{{x}_{{bu}}}$, happening concurrently to the one at ${\left.x\right|}_{{x}_{{bu}}}$, it would result from reshaping a section of a core fed through ${\left.(x+{{{{\delta }}}})\right|}_{{x}_{{bu}}}$ shorter by $\delta$ compared to the one fed through ${\left.x\right|}_{{x}_{{bu}}}$ for the same moment in time, as is demonstrated in Fig. 2c. On the other hand, the concurrent pinch-off at ${\left.(x-{{{{\delta }}}})\right|}_{{x}_{{bu}}}$ would result from a section of a core longer by ${{{{\delta }}}}$. In other words, ${\left.\left({dx}/d\lambda \right)\right|}_{{x}_{{bu}}}=-\!1$. Noting that ${\left.\frac{d}{{dx}}\right|}_{{x}_{{bu}}}=\,{\left.\left(\frac{\partial }{\partial x}+\frac{{dx}}{d\lambda }\cdot \frac{\partial }{\partial \lambda }\right)\right|}_{{x}_{{bu}}}={\left.\left(\frac{\partial }{\partial x}-\frac{\partial }{\partial \lambda }\right)\right|}_{{x}_{{bu}}}$, and assuming that *γ* is independent of temperature (or, at least, has a much weaker dependence on temperature than the cladding viscosity, which is valid for material combinations that we consider here), we can finally write the two equations of our model as:

Here, ${\eta }_{{clad}}$ is the viscosity of silica, ${\eta }_{{{SiO}}_{2}}\left(T\left(x\right)\right)=A{e}^{B/T(x)}$, with $A=5.7\times {10}^{-8}$ ${Pa}\cdot s$ and $B=61812$ *K* material constants of silica^36^. *β* is likely to depend on the viscosity contrast between the cladding and the core^24^ and for increasing contrast is likely to asymptotically converge to a constant. *β* is the free parameter in the current model and has to be determined from ab-initio calculations of the full Navier-Stokes equations^33^. Interestingly, if rearranging Eq. (2) and (3) to formulate it in terms of ${F}^{{v}_{f}}\left(\lambda,x\right)\equiv {t}_{g}-{t}_{{pinch}}$ rather than ${{{{{\rm{Ca}}}}}}={{{{{t}}}}}_{{{{{pinch}}}}}/{{{{{t}}}}}_{{{{{g}}}}}$, as initially formulated, we arrive atwhich can be derived using purely geometrical arguments (see Supplementary Note 1):

Equivalent to Eqs. (2), (4) simply means that the droplet grows as long as it has not pinched off;

Equivalent to Eqs. (3), (5) means that the pinch-off location is unique because, at any other location, the droplet would need longer to pinch-off than it is allowed to grow.

${F}^{{v}_{f}}\left(\lambda,x\right)$ can be rewritten as ${{F}^{{v}_{f}}(\lambda,x)|}_{{x}_{{bu}}}={t}_{g}-{t}_{{pinch}}={(\lambda /{v}_{f}-\beta a{\eta }_{{clad}}/\gamma )|}_{{x}_{{bu}}}$, which multiplied by a constant $C=2\pi {a}{v}_{f}\gamma$, results in $C{F}^{{v}_{f}}(\lambda,x)={(2\pi a\gamma \lambda -2\pi {a}^{2}\beta {\eta }_{{clad}}{v}_{f})|}_{{x}_{{bu}}}$. Note that:

${\left.2\pi a\gamma \lambda \right|}_{{x}_{{bu}}}$ is the energy stored in the fluid thread of length ${\left.\lambda \right|}_{{x}_{{bu}}}$, or the potential energy $({{{{{\rm{PE}}}}}})$ stored in the pinching-off section of a thread before the development of capillary instability in it;

$2\pi {a}^{2}\beta {\eta }_{{clad}}{v}_{f}|_{{x}_{{bu}}}$ is the work invested in overcoming the viscosity forces to cause the pinch-off, or the kinetic energy $({{{{{\rm{KE}}}}}})$ imparted to the pinching-off section of a thread by the progression of the instability towards the breakup.

In other words, $C{F}^{{v}_{f}}\left(\lambda,x\right)$ is, by definition, the Lagrangian $L={{{{{\rm{PE}}}}}}-{{{{{\rm{KE}}}}}}$ of the system. In fact, we can claim that the AVG-IM is equivalent to a description of the system in terms of Euler-Lagrange mechanics (for detailed proof, see Methods and Supplementary Note 2).

Finally, it is essential to formulate the validity limitations (VL’s) of the model. We ascertain (see Methods) that the model is valid when:

VL1: The breakup period ${\left.\lambda \right|}_{{x}_{{bu}}}$ is comparable to the width of the hot-zone boundary $w$, and

VL2: The pinch location ${x}_{{bu}}$ is within the hot-zone boundary $w$. In other words, the pinch time ${t}_{{pinch}}$ is comparable to the fiber dwell time in the hot zone boundary $w/{v}_{f}$.

Validity limitations (VL1 + VL2) explain the heuristic observation^7,19^ that the width of the liquified region boundary and the feed speed are the user-controlled “tuning knobs” for the breakup period (${{\lambda }} |_{{{{{x}}}}_{{{{{bu}}}}}} \, \approx \, {{{{w}}}}$) and the pinch-off location $({x}_{{bu}} \sim {v}_{f}\cdot {t}_{{pinch}} \, \approx \, {{{{w}}}})$.

### Corroboration of the AVG-IM against experimental results and ab-initio calculations

To validate the AVG-IM, we compare it to the experimental breakup in silicon-core silica fibers. The silica-silicon interface is well studied in the microelectronics realm, with the interface tension $\gamma=0.3$ $J/{m}^{2}$ measured experimentally elsewhere^37^ (see Supplementary Note 3). We obtain $\beta$ by corroborating our model against the ab-initio Navier-Stokes equation solution, verified in previous works^30,31,33^.

We assume that an exponential function of the form below can describe the shape of the temperature profile at the hot-zone boundary:

6 $$T\left(x\right)={T}_{\max }-({T}_{\max }-{T}_{{Si}}){e}^{-x/w}$$

where ${T}_{\max }$ is the maximal asymptotic temperature, ${T}_{{Si}}$ is the melting temperature of silicon, and $w$ defines the profile width. This approximate profile shape is based on two arguments. The first is purely heuristic: such an exponent is a monotonic function asymptotically converging to a maximal value ${T}_{\max }$ following a growth region of a width $w$. This fits a qualitative behavior of the flame: it assumes its maximal temperature ${T}_{\max }$ in its center following a monotonic growth within its boundary of a width $w$. The second argument is mathematical: complex exponentials form a complete set, i.e., any function, without the loss of generality, can be approximated by a set of exponentials, and thus $T\left(x\right)$, in its approximate form, is just a first-order decomposition of the real temperature profile into exponential components.

**Fig. 4:**
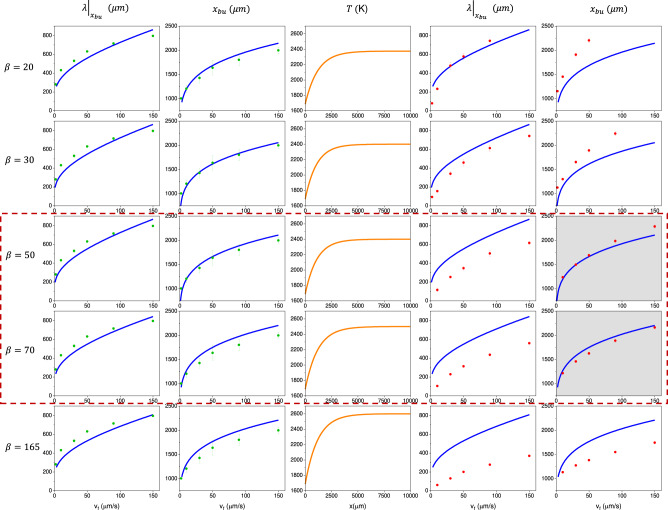
Comparison between experimental data, AVG-IM, and ab-initio simulation, assuming *β* values from 20 to 165 (From top to bottom, in rows). Results for $\beta=50$ and $\beta=70$ are highlighted by a red dashed box. The improved fit between the model-derived and simulation-derived ${x}_{{bu}}$ (the light-gray background highlights the best-fit graphs) signifies the range where the optimized $\beta$ falls, i.e., $\beta=60\pm 10$. From left to right: Columns 1 and 2: Experimental results (green circles) for the breakup period ${\left.\lambda \right|}_{{x}_{{bu}}}$ and ${x}_{{bu}}$, compared to the fitted model (solid blue line); Column 3: Temperature profile obtained by fitting AVG-IM to the experimental values and implemented in the ab-initio simulations. Columns 4 and 5: ab-initio simulation results (red circles) for ${\left.\lambda \right|}_{{x}_{{bu}}}$ and ${x}_{{bu}}$, and model predictions. Error bars – 1 σ.

We note that no reliable technique for directly measuring the temperature profile in breakup experiments exists. The most promising approach demonstrated so far is a reconstruction of the temperature profile from the core emissivity^38^, demonstrated in the recrystallization of silicon-germanium-core silica fibers. In laser recrystallization, the core is held at temperatures that don’t significantly exceed the melting point, just by a few tens of degrees. Hence, the intensity range of the core’s optical emission signal is relatively low. In the breakup process, on the other hand, the range of temperatures to be captured is of the order of one thousand degrees (Fig. 4). This necessitates a custom-developed image-capturing setup with a high dynamic range and, simultaneously, low noise, so the details, such as the dip in emissivity signal due to the melting of silicon (see Supplementary Note 4), are reliably captured.

Starting from $\beta$ = 20, we perform a monotonic parametric scan of $\beta$ ending at 165. For each chosen value of $\beta$, the temperature profile parameters ${T}_{\max }$ and $w$ were determined through an algorithm that minimizes the difference between the AVG-IM predictions for ${\left.\lambda \right|}_{{x}_{{bu}}}$ and ${x}_{{bu}}$ and the experimentally obtained data. In all cases, the maximal temperature experienced by the fiber in the simulations does not surpass the boiling point of silica.

The algorithm results in a 2D map (see Supplementary Note 5) of the model-to-experiment fit error as a function of the temperature profile parameters, yielding a single combination of ${T}_{\max }$ and $w$ minimizing it. Figure 4 shows the experimental data and the AVG-IM fit resulting from this process (Fig. 4, Columns 1, 2). The determined temperature profile (Fig. 4, Column 3) was then used in ab-initio simulation of breakup. Finally, the results of ab-initio calculations were corroborated against the model predictions (Fig. 4, Columns 4, 5), with optimal correspondence for ${{{{{\rm{\beta }}}}}}=60\pm 10$.

Here, it is important to notice that the level-set method implemented^33^ in the simulations introduces a simplification in the time propagation of the Navier-Stokes equations, as a result of which the mass—and consequently volume—is not conserved (see Supplementary Movie 1). For this reason, we consider that the breakup period data from the simulations may be trusted qualitatively, while the breakup location data can be trusted quantitatively.

**Fig. 5:**
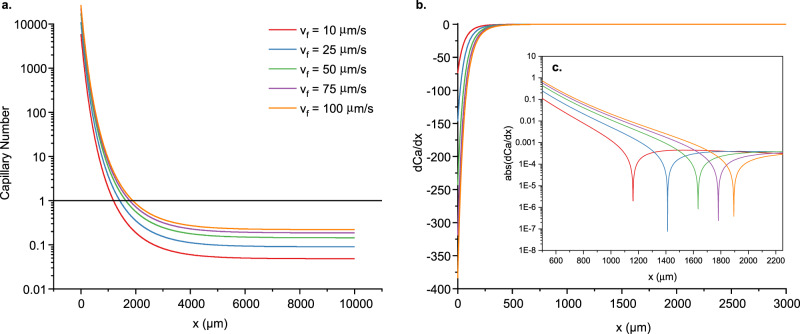
Evolution of the capillary number for *β*=60. **a** Capillary Number as a function of the distance from the melting point of Si, for selected feed speeds *v*_*f*_ (in µm/s), graphically representing Eq. (2). **b** Derivative of the Capillary Number for the same feed-speed set, graphically representing Eq. (3). **c** The absolute value of $\frac{d \rm Ca}{{dx}}$ in logarithmic scale is shown to illustrate better the extreme points corresponding to the breakup position ${x}_{{bu}}$.

As the capillary instability develops, the lack of mass conservation results in the core thinning out in addition to pinching, causing the pinch process to happen faster, at a higher rate. If assuming a steady-state process, the period of such a simulated breakup is expected to be shorter than experimentally observed. At the same time, to even start reshaping into a droplet that eventually pinches off, the core needs to reach the viscosity where Ca=1 (Fig. 5a), allowing such a reshaping. Such viscosity conditions translate one-to-one into the appropriate breakup location (Fig. 5a, c), unimpacted by the lack of volume conservation.

**Fig. 6:**
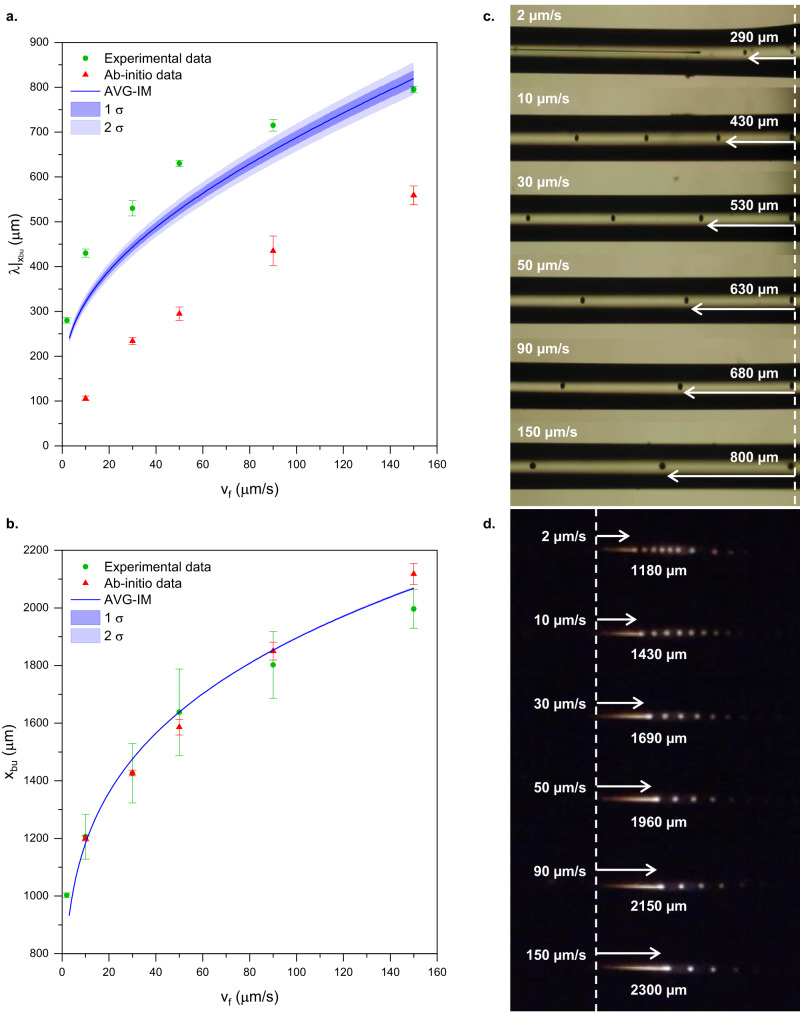
Experiments, AVG-IM, and ab-initio simulations are in accord for *β* = 60. Experimental data, ab-initio simulation results and AVG-IM predictions for **a**—the breakup wavelength ${\lambda }_{{bu}}$ and **b—**the breakup location ${x}_{{bu}}$ for a varying feed speed, assuming $\beta=60$ and an exponential temperature profile with ${T}_{\max }=2475\pm 25K$ and $w=1150\pm 50\mu m$. The shaded region around the model prediction indicates the error (one and two ${{\rm{\sigma}}}$), Error bars on experimental and simulation data **–** 1 σ. Optical micrographs of the breakup: **c** Microscope image of fiber post-breakup indicating the breakup period resulting from AVG breakup for the respective feed speeds; **d** Snapshots of the AVG breakup experiment at different feed speeds, where the breakup location can be identified as the distance of the pinch-off from the melting position of silicon (indicated by the dashed vertical line). The experiments were performed in 280 μm thick silica fiber with 4 μm Si core by feeding it through a hydrogen-oxygen flame resulting from a flow of 0.30 l/min of H_2_, 0.10 l/min of O_2_ (also see Methods).

Choosing $\beta=60$, AVG-IM quantitatively reproduces the entire set of the experimental data and the pinch-off positions calculated from the first principles (Fig. 6, Supplementary Movie 2). Albeit with the benefit of fitting *β*, this is the first time any ab-into-corroborated analytical model fits the experimental results quantitatively^19,30,31^.

Previously^26^, using COMSOL^39^, we evaluated $\beta$ for the breakup in a slightly different system—a stationary fiber experiencing increasing local liquefaction (see Supplementary Note 6). Comparing measured and simulated pinch-off times, we found $\beta$ to be approximately 65, in line with the results presented here.

Generally, parameters defining the breakup behavior divide into two groups: the first relates to the fiber, while the second—to the liquefaction zone it is fed through. The fiber-related properties are the dimensions and viscosities of the core and cladding and the interface energy between the two (five parameters). The relevant liquefaction zone properties are its maximal temperature and the width of its boundary (two parameters).

There are two Model Simplifications (MS’s) that AVG-IM makes, narrowing the space of parameters:

MS1: The cladding-to-air interface of the fiber is not significantly prone to capillary instability; thus, the cladding can be considered of an infinitely large diameter. It allows us to suggest that the model is valid for tapered-cladding geometries^40^, recently acquiring technological relevance. We have verified experimentally (see Supplementary Note 9) that AVG-IM is valid for adiabatically tapered geometries of both core and cladding. For non-adiabatic tapers, i.e., when $a=a(x)$ on a scale comparable to the breakup wavelength, we suggest that the model can be extended to the tapered geometries by replacing ${{{{{\rm{Ca}}}}}}$ in Eqs. (2) and (3) with a modified ${{{{{{\rm{Ca}}}}}}}^{{taper}}(\lambda,x)\equiv \frac{\beta a(x){\eta }_{{clad}}(x){v}_{f}}{\gamma \lambda }$.

MS2: The fiber cores are solid crystalline materials that, upon melting, abruptly change the aggregation state, becoming inviscid liquid; thus, core-cladding viscosity contrast is considered negligible. It holds for a wide variety of core materials relevant for silica-fiber embedded optoelectronics and photonics, including but not limited to semiconducting, metallic, magnetic, optically nonlinear, superconducting, thermoelectric, ferroelectric, and piezoelectric^6,7,19,41–47^. For fibers where ${{{{{\eta }}}}}_{{{{{core}}}}}/{{{{{\eta }}}}}_{{{{{clad}}}}}$ is finite, such as polymer-cladding chalcogenide/polymer-core^8,17,32^, ${{{{{\rm{Ca}}}}}}$ in Eqs. (2) and (3) needs to be replaced with ${C{{{{{\rm{a}}}}}}}^{\frac{{\eta }_{{core}}}{{\eta }_{{clad}}}}(\lambda,x)\equiv \beta \cdot {v}_{f}/\left[2\lambda \cdot {in}\left(\lambda,{\eta }_{{clad}}(x),{\eta }_{{core}}(x)\right)\right]$, where ${{{{{\rm{in}}}}}}\left({{{{\lambda }}}},{{{{{\eta }}}}}_{{{{{clad}}}}},{{{{{\eta }}}}}_{{{{{core}}}}}\right)$ is the Tomotika instability rate. This choice is guided by the fact that ${{{{{{\rm{Ca}}}}}}}^{\frac{{\eta }_{{core}}}{{\eta }_{{clad}}}}(\lambda,x)$ converges towards ${{{{{\rm{Ca}}}}}}\left(\lambda,x\right)$ for ${\eta }_{{core}}/{\eta }_{{clad}}\to 0$, as is demonstrated in Methods.

MS1 and MS2 under a reasonable assumption that the cladding material is uniform, and thus the spatiotemporal temperature profile fully defines the viscosity of the cladding, leaves us with only four parameters for which the AVG-IM validity must be verified: the maximal temperature of the liquefaction zone ${T}_{\max }$, its boundary width $w$, the core-cladding interface energy $\gamma$, and the core radius $a$. To validate AVG-IM in the subspace spanned by these parameters, we will define their heuristic, behavioral trends, and relevance limits and then scan them one by one within those limits to compare the AVG-IM predictions resulting from each scan to the Navier-Stokes equations solved ab-initio for the accuracy of extrapolation.

**Fig. 7:**
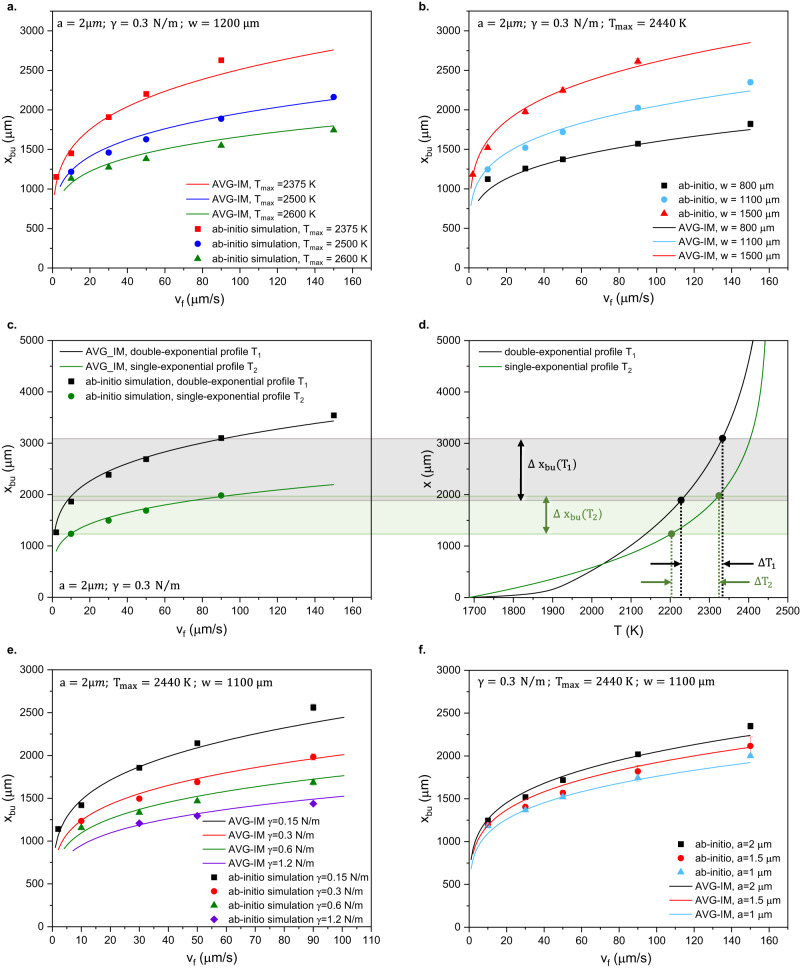
Broad extrapolation of AVG-IM compared to ab-initio solutions of the Navier-Stokes equations. **a** Validation of AVG-IM for varying maximal temperature of the liquefaction zone ${T}_{\max }$. **b** Validation of AVG-IM for varying liquefaction zone boundary width w. **c**, **d** Validation of AVG-IM for complex liquefaction boundary geometry. **c** Pinch position ${x}_{{bu}}$ resulting from the single- and double-exponential temperature profiles, ${T}_{1}$ and ${T}_{2}$. **d** Temperature profiles, ${T}_{1}$ and ${T}_{2}$. Notation: $\Delta {x}_{{bu}}({T}_{1})$ is ${x}_{{bu}}$ $({T}_{1})$ in the range of ${10 \, < \, v}_{f} \, < \, 90\mu m/s$(light grey rectangle); $\Delta {x}_{{bu}}({T}_{2})$ is ${x}_{{bu}}$ $({T}_{2})$ in the range of ${10 \, < \, v}_{f} \, < \, 90\mu m/s$ (light green rectangle). ${\Delta T}_{1}$ and ${\Delta T}_{2}$ are the temperature intervals corresponding to $\Delta {x}_{{bu}}({T}_{1})$ and $\Delta {x}_{{bu}}({T}_{2})$, respectively. **e** Validation of AVG-IM for varying core-cladding interface energy $\gamma$. **f** Validation of AVG-IM for varying core radius $a$. Error bars on simulation data – 1 σ.

Let us start by examining the liquefaction zone properties. The maximal temperature of the liquefaction zone ${T}_{\max }$ resulting from the hydrogen-oxygen flame would vary with the flame oxygenation (H_2_:O_2_ flow rate ratio), the overall gas-flow rate, and the geometry of the torch orifice by hundreds of degrees, while its boundary width $w$ would vary on a sub-millimeter to a millimeter scale. For the liquefaction zone resulting from a laser beam, ${T}_{\max }$ and $w$ would vary with the beam intensity, size of the focal spot, and beam profile. In line with those scenarios and assuming the temperature profile defined by Eq. (6), Fig. 7a demonstrates the influence of the ${T}_{\max }$ on the pinch-off location ${x}_{{bu}}$ calculated from ab-initio and from AVG-IM for $\beta=60$ in a window of temperatures around that of the etalon experiment in Fig. 6 and for the same fiber properties. Similarly, Fig. 7b demonstrates the influence of the liquefaction zone boundary width $w$ on the ab-initio- and AVG-IM-calculated ${x}_{{bu}}$.

In general, Eq. (6) doesn’t necessarily describe the accurate temperature profile. Figure 7c demonstrates an example of AVG-IM validation for the temperature profile that can’t be approximated by the first-order decomposition in exponentials, i.e., the single-exponential. The next in its complexity is the second-order decomposition, i.e., the double-exponential. In Fig. 7c, d, we corroborate the ${x}_{{bu}}$ resulting from the double-exponential profile

7 $${T}_{1}(x)={T}_{\max }-({T}_{\max }-{T}_{{Si}})(C{e}^{-x/{w}_{1}}+(1-C){e}^{-x/{w}_{2}})$$

where ${w}_{1}=2000\mu m$, ${w}_{1}=50\mu m$, and $C=0.77$, against ab-initio and the ${x}_{{bu}}$ resulting from the single exponential profile ${{{T}}}_{2}\left({{{{{\rm{x}}}}}}\right)={{{T}}}_{\max }-({{{T}}}_{\max }-{{{T}}}_{{{{{{\rm{Si}}}}}}}){{{{{{\rm{e}}}}}}}^{-{{{{{\rm{x}}}}}}/{{{{{\rm{w}}}}}}}$, where $w=1100\mu m$. ${T}_{\max }=2450{K}$ for both profiles. For the reasoning behind the specific choice of ${T}_{1}$ and ${{{{{T}}}}}_{2}$, see Methods.

Figure 7c, d overlays the pinch-off positions ${x}_{{bu}}({T}_{1})$ and ${x}_{{bu}}({T}_{2})$ against ${T}_{1}$ and ${T}_{2}$ in the way that allows backtracking of the temperatures corresponding to the pinch positions defined by each feed speed for both temperature profiles. From this backtracking, it is apparent that the fiber exposed to ${T}_{2}$ has overall shorter ${x}_{{bu}}$, i.e., it pinched sooner than the one exposed for ${T}_{1}$, even though the fiber exposed to ${T}_{1}$ entered sooner the soft silica’s temperature regime (>1900 K), where the instabilities can develop. Additionally, for a given feed speed range, while the corresponding pinch-off location range $\Delta {x}_{{bu}}({T}_{{{{{\mathrm{1,2}}}}}})$ depends heavily on the temperature profile geometry ${T}_{{{{{\mathrm{1,2}}}}}}$ overall, the range of temperatures ${\Delta T}_{{{{{\mathrm{1,2}}}}}}$ defining the $\Delta {x}_{{bu}}({T}_{{{{{\mathrm{1,2}}}}}})$, within a reasonable approximation, is invariant of the overall shape of ${T}_{{{{{\mathrm{1,2}}}}}}$.

In other words, for a given fiber undergoing a predictable AVG breakup at a given feed speed, the pinch-off location is defined by the local viscosity ${\left.{{{{{\rm{\eta }}}}}}\left({{{{{\rm{x}}}}}}\right)\right|}_{{{{{{{\rm{x}}}}}}}_{{{{{{\rm{bu}}}}}}}}$ at its vicinity only, regardless of the fluid dynamic history that the core has experienced prior to reaching this location. One can think of ${\left.{{{{{\rm{\eta }}}}}}\left({{{{{\rm{x}}}}}}\right)\right|}_{{{{{{{\rm{x}}}}}}}_{{{{{{\rm{bu}}}}}}}}$ as a phase filter for the breakup. Additionally, from Eqs. (2) and (3) it follows that ${\left.\lambda (x)\right|}_{{x}_{{bu}}}$ is defined by the viscosity gradient ${\left.\frac{\partial {{{{{\rm{\eta }}}}}}\left({{{{{\rm{x}}}}}}\right)}{\partial {{{{{\rm{x}}}}}}}\right|}_{{{{{{{\rm{x}}}}}}}_{{{{{{\rm{bu}}}}}}}}$ only, since for a given feed speed Eqs. (2) and (3) have only two inputs: the cladding viscosity ${{{{{\rm{\eta }}}}}}\left({{{{{\rm{x}}}}}}\right)$ and its gradient $\frac{\partial {{{{{\rm{\eta }}}}}}\left({{{{{\rm{x}}}}}}\right)}{\partial {{{{{\rm{x}}}}}}}$. Thus, $\frac{\partial {{{{{\rm{\eta }}}}}}\left({{{{{\rm{x}}}}}}\right)}{\partial {{{{{\rm{x}}}}}}}$ is the notch filter for the breakup wavelength mentioned in the abstract.

Importantly, this local nature of the predictable AVG breakup relaxes the requirements for the temperature profiles reconstructed by the AVG-IM from the experimental results, such as those in Fig. 6, since they need to represent the real-world temperature accurately in a limited range of $\Delta T$, defined by the experimentally attainable feed speeds, while a typical $\Delta T$ is a hundred to two hundred degrees, as in Fig. 7d.

Finally, Fig. 7e examines the influence of the core-cladding interface tension $\gamma$ on AVG-IM in correspondence to the ab-initio predictions. Note that once the cladding viscosity is set, a choice of $\gamma$ emulates the choice of core material for AVG-IM under the simplifications MS1 and MS2^48^. Figure 7e implies that for the core materials with very dissimilar $\gamma$’s encapsulated in the same cladding and experiencing the breakup within the same temperature profile, the pinch-off locations will be increasingly separated axially for the increasing feed speed. This separation can exceed millimeters, becoming comparable to the overall liquefaction zone width $W$, especially if the heat source is well-localized, such as a laser spot.

This last result, in combination with the ability to select the phase and the period of the breakup independently, culminates in the proposition of the following “staggered” scenario for the scalable assembly of in-fiber devices. Let’s consider two cores, ${c}_{1}$ and ${c}_{2}$, with roughly similar radii but made of dissimilar materials, such that ${\gamma }_{1} \, > \, {\gamma }_{2}$ within a single cladding. Let’s consider two heat sources ${T}_{1}$ and ${T}_{2}$ with maximal temperatures ${T}_{\max }^{1} \, < \, {T}_{\max }^{2}$, boundary widths ${w}_{1}$, ${w}_{2}$, and overall widths of ${W}_{1}$, ${W}_{2}$, aligned along the fiber axis at a distance $\Delta$ from each other. If ${v}_{f}$ exists such that the pinch-off location of the first core ${c}_{1}$ is at ${x}_{{bu}}^{1}({v}_{f})$ within ${w}_{1}$, yet the pinch-off location of the second core ${c}_{2}$ in the first (colder) heat source ${T}_{1}$ is at ${x}_{{bu}}^{2}({v}_{f})$ that exceeds its overall width ${W}_{1}$, it will follow that ${c}_{1}$ will breakup periodically with the wavelength ${\left.\lambda \right|}_{{x}_{{bu}}^{1}({v}_{f})}$ within the first heat source, while ${c}_{2}$ will pass through intact. Now, ${T}_{2}$ can be tuned, such that ${c}_{2}$ breaks up in the second heat source ${T}_{2}$ with a wavelength ${\left.\lambda \right|}_{{x}_{{bu}}^{2}({v}_{f})}$ that is equal to ${\left.\lambda \right|}_{{x}_{{bu}}^{1}({v}_{f})}$. Next, the distance between the heat sources $\Delta$ can be tuned such that $\Delta=N\cdot {\left.\lambda \right|}_{{x}_{{bu}}^{{{{{\mathrm{1,2}}}}}}({v}_{f})}$, where $N$ is any positive integer. As a result, both cores will break up in-phase, with the same period in an axially staggered fashion. If the initial cores ${c}_{{{{{\mathrm{1,2}}}}}}$ are spaced in the fiber cross-section closer than the sum of the spheres’ radii pinching off those cores, the spheres from the neighboring cores will get in contact in the breakup process and form an axial array of fused bi-spherical entities.

**Fig. 8:**
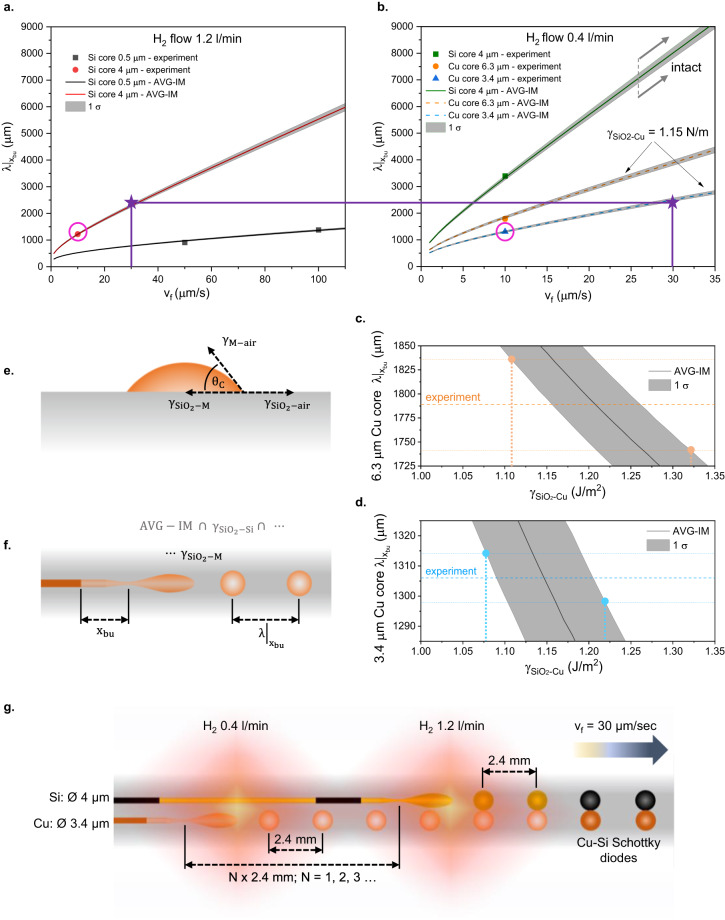
Obtaining the conditions for the in-fiber staggered assembly of Cu-Si Schottky diodes. **a** Experimental breakup and its AVG-IM analysis of Si-core breakup in the ${H}_{2}=1.2l/\min$ flame. AVG-IM fit yields ${T}_{\max }=2185K$ and $w=1275\mu m$. **b** Experimental breakup and AVG-IM analysis of Si- and Cu- core fibers in the ${H}_{2}=0.4l/\min$ flame. AVG-IM fit to the Si data, knowing that the boundary width $w$ is identical (see Supplementary Note 10 and Methods), yields ${T}_{\max }=2065K$. Notations: in **a** and **b** pink circles highlight the experimental results with similar breakup wavelengths at identical feed speed; purple stars signify the proposed conditions for the staggered device assembly in **g**. **c**, **d** Obtaining silica-copper interface energy ${\gamma }_{{{SiO}}_{2}-{Cu}}$ by extrapolating AVG-IM found for the Si core to the experimental results of the Cu-core breakup: **c** for the $2a=6.3\mu m$ core, and **d** for the $2a=3.4\mu m$. **e**, **f** Methods of interface energy measurement for a melt $M$ and silica ${\gamma }_{{{SiO}}_{2}-M}$: **e** Sessile-drop method (conventional) determines ${\gamma }_{{{SiO}}_{2}-M}={\gamma }_{{{SiO}}_{2}-{air}}-{\gamma }_{M-{air}}\cdot \cos {\theta }_{C}$, given the silica-air interface energy ${\gamma }_{{{SiO}}_{2}-{air}}$ and melt-air interface energy ${\gamma }_{M-{air}}$; **f** Fiber-AVG-IM method (new, proposed in this study) determines ${\gamma }_{{{SiO}}_{2}-M}$ by extrapolating the AVG-IM-obtained temperature profile for the reference core material (Si in our case) to the breakup results of the core material M in silica fiber. **g** Staggered Cu-Si diodes assembly schematics and suggested initial conditions based on AVG-IM analysis of Si and Cu breakup results in **a** and **b**. For the breakup periods to match exactly, the flow of hydrogen at the hotter source can be finetuned around the initial setpoint of ${H}_{2}=1.2l/\min$. The exact phase-matching between the two breakups can be achieved by finetuning the distance between the two heat sources, moving the hotter source along the fiber axis further away from the colder one. Error bars – 1 σ.

**Fig. 9:**
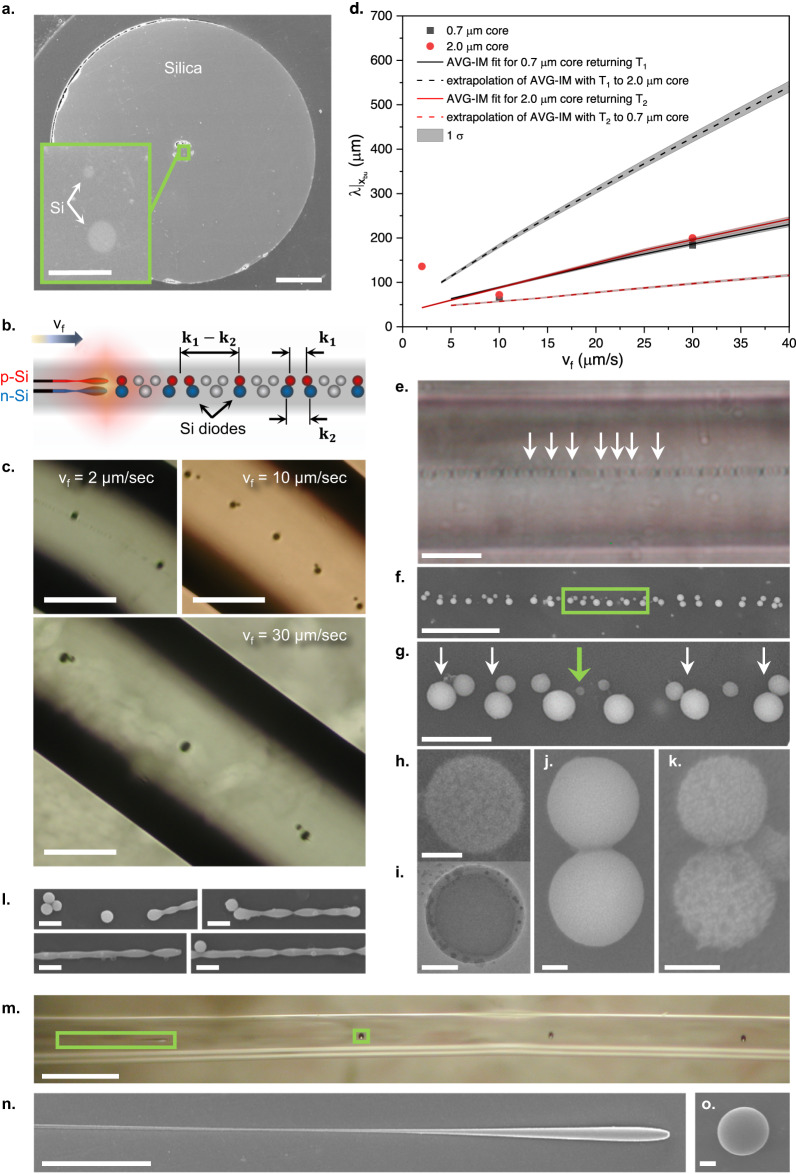
Breakup in multicore fibers. **a** Double Si-core fiber cross-section (scalebar – 50 µm); inset - zoom-in to the cores (scalebar – 5 µm). **b** Interference device assembly: ${{{{{{\bf{k}}}}}}}_{{{{{{\bf{1}}}}}}}\,$ and $\,{{{{{{\bf{k}}}}}}}_{{{{{{\bf{2}}}}}}}$ are the breakup wavevectors for the thinner and thicker cores, respectively. **c** Breakup of the fiber in **a** (scalebars – 100 µm). **d** AVG-IM analysis of the results in **c** using double-exponential profiles ${T}_{1,2}$. **e**–**k** Breakup of the fiber scaled down from the fiber in **a**. **e** Optical micrograph (scalebars – 10 µm). The ${{{{{{\bf{K}}}}}}{{{{{\boldsymbol{=}}}}}}{{{{{\bf{k}}}}}}}_{{{{{{\bf{1}}}}}}}{{{{-}}}}{{{{{{\bf{k}}}}}}}_{{{{{{\bf{2}}}}}}} \, \approx \, 0.3{Hz}/\mu m$ quasiperiodic beat (white arrows) is apparent. **f** The Scanning Electron Microscope (SEM) micrograph (scalebar – 5 µm) of the Si spheres released from the fiber in **e**. **g** SEM zoom in (scalebar – 1 µm) to a single beat period (green rectangle in **f**). White arrows point to the contacted bi-spherical assemblies. The green arrow points to the smallest individual sphere observed. **h** SEM zoom in (top) and **i** tunneling Electron Microscope (TEM) zoom in (bottom) to the smallest sphere observed (scalebars – 50 nm). **j** SEM of a typical bi-spherical assembly (scalebar – 100 nm). **k** SEM of the smallest bi-spherical assembly observed (scalebar – 100 nm). **l** The onset of the VL2-violating breakup. Shown is the SEM micrograph of an incompletely broken 350 nm thick Si core quenched before entering the flame completely. **m**–**o** The onset of predictable AVG breakup. **m** The optical micrograph of the $2{a}=4\mu m$ fiber broken up in H_2_ 0.8 l/min flame at a feed speed of 10 µm/s (see Supplementary Note 10). **n** The droplet forming at the core tip entering the flame quenched before its pinch-off is complete. The SEM micrograph of the core section in the green rectangle in **m** is shown. **o** The SEM micrograph of a sphere in the green square in **m**. Error bars on experimental data – 1 σ.

In the analysis presented in Fig. 8, we obtain specific conditions for assembling Cu-Si Schottky diodes in a double-core fiber using such a coherently staggered multicore breakup. It should be noted from Fig. 7f that ${x}_{{bu}}$ has a weaker dependence on the core radius $a$ than on any other parameter of the model, and thus staggered in-fiber assembly is unlikely to be achieved for the same-material devices, such as Si pn-diodes, by simply choosing different radii for the p-doped and n-doped cores. For that case, a different “interference” scenario is suggested in Fig. 9.

### Examples of practical uses of AVG-IM in fiber-device engineering, assessment of AVG-IM general applicability limits

Investigating the breakup of Si- and Cu-core fibers in a pure hydrogen flame for the ability to yield same-wavelength breakup in disparate-material cores (see Supplementary Note 10), we notice that there are at least two sets of conditions generating very similar breakup wavelengths at ${v}_{f}=10\mu m/s$, which gives us a starting point for looking for a staggered diode assembly scenario: ${\lambda |}_{{x}_{{bu}}}=1220\pm 20\mu m$, in $4\mu m$ thick Si-core, @ H_2_ flow rate = 1.2 l/min; and ${\lambda |}_{{x}_{{bu}}}=1310\pm 20\mu m$, in $3.4\mu m$ thick Cu-core, @ H_2_ flow rate = 0.4 l/min.

Those exact conditions, though, won’t lead to the desired outcome, since the $2a=4\mu m$ Si core at *v*_*f*_ = 10 *μm*/*s* breaks in H_2_ flame of 0.4 l/min as well, with a longer ${\lambda |}_{{x}_{{bu}}}=3400\pm 60\mu m$, while to qualify for the staggered assembly scenario, the core with lower $\gamma$ should pass the colder flame intact.

Worth mentioning is the set of conditions that resulted in the random breakup (see Supplementary Note 10, Fig. 1g), and thus cannot be represented as a point in Fig. 8a: ${\left.\lambda \right|}_{{x}_{{bu}}}={random}$, in $0.5\mu m$ thick Si-core, H_2_ flow rate = 1.2 l/min, $2a=0.5\mu m$, and ${v}_{f}=10\mu m/s$. It allows us to assess experimentally the limitation of AVG-IM validity, violated when the feed speed becomes too slow. Note that the AVG-IM validity limitation VL2 is written as ${x}_{{bu}} \sim {v}_{f}\cdot {t}_{{pinch}} \, \approx \, {{{{{\rm{w}}}}}}$, defines a characteristic feed speed,

8 $${v}_{f}^{{VL}2}\left(a,T(x)\right)\equiv \frac{{{{{{\rm{w}}}}}}}{{t}_{{pinch}}}=w\cdot \frac{\gamma }{\beta a{\left.\eta \left(x\right)\right|}_{{x}_{{bu}}=w}}$$

which is the feed speed that, for a given temperature profile and core radius, results in the pinch location ${x}_{{bu}}$ occurring precisely the distance of the boundary width $w$ into the liquefaction zone. It is the feed speed at which the breakup is safely predictable. If the feed speed is much faster than ${v}_{f}^{{VL}2}$, the core is expected to reach the uniform temperature too soon and fall into a Tomotika-like jetting regime.

Yet surprisingly, we find experimentally that the opposite is also true: the feed speed is much slower than ${v}_{f}^{{VL}2}$ results in the core not experiencing a viscosity gradient high enough by the time of the breakup, and thus also falling into an effectively Tomotika-like jetting regime. Based on the temperature profile obtained by AVG-IM for H_2_ flow rate of 1.2 l/min, we calculate ${v}_{f}^{{VL}2}=67\mu m/s \, \gg \, {v}_{f}=10\mu m/s$ for $0.5\mu m$ thick Si core. For comparison, we calculate ${v}_{f}^{{VL}2}=8\mu m/s \, \approx \, {v}_{f}=10\mu m/s$ for the $4\mu m$-thick Si core, which indeed sticks to the AVG-IM behavior, as can be seen in Fig. 8a. Thus, ${v}_{f}^{{VL}2}\left(a,{T}(x)\right)\approx {v}_{f}$ is a reliable, practical AVG-IM validity criterion.

To extrapolate AVG-IM to the Cu-core fiber results in Fig. 8b, we treat the temperature profile as set to the one found for ${H}_{2}=0.4{l}/\min$ based on Si-core data and use the core-cladding interface energy $\gamma$ as the free fitting parameter. As is demonstrated in Fig. 8c, d, the last step results in the best fit of AVG-IM to the Cu-core of $2a=3.4\mu m$ at ${\gamma }_{{{SiO}}_{2}-{Cu}}=1.15\pm 0.07{J}\cdot {m}^{-2}$, and best fit to the Cu-core of $2a=6.3\mu m$ at ${\gamma }_{{{SiO}}_{2}-{Cu}}=1.21\pm 0.10{J}\cdot {m}^{-2}$.

Two Cu-core results combined, we measure ${\gamma }_{{{SiO}}_{2}-{Cu}}$ to be $1.20\pm 0.12{J}\cdot {m}^{-2}$ using Si-core fiber as a reference and AVG-IM as the analysis framework. In fact, as is demonstrated in Fig. 8e, f, this sets a novel method for measuring interface energy between two melts, potentially more accurate than the conventional sessile-drop method^48^. For a detailed comparison between the two measurement approaches, see Methods.

Here, we can finally propose the conditions for the staggered Cu-Si Schottky diodes assembly in a double-core fiber. AVG-IM for $2a=4\mu m$ Si-core fiber @ ${H}_{2}=0.4l/\min$ predicts breakup periods exceeding the width of the flame $W=7.2{mm}$ (see Supplementary Note 10) for the feed speeds exceeding $27\mu m/s$. Recalling that, ${x}_{{bu}}$ for any dataset in this study was always larger than the corresponding ${\left.\lambda \right|}_{{x}_{{bu}}}$, it’s clear that ${x}_{{bu}} \, > \, W$ for ${v}_{f} > 27\mu m/s$ as well.

Thus, if a double-core fiber with $2a=3.4\mu m$ Cu core and $2a=4\mu m$ Si core is fed at ${v}_{f}=30\mu m/s$ through a combination of two heat sources, the colder of which is the flame @ ${H}_{2}=0.4l/\min$, and the hotter is the flame @ ${H}_{2}=1.2l/\min$, the colder source first, the Cu core will break up in the colder source, while the Si core will pass intact and will break up later on in the hotter source, both with the breakup wavelength of ~2.4 mm. Those conditions, highlighted by purple stars in Fig. 8a, b, provide a starting point which, after a slight finetuning of the synchronization between the two breakups, as is described in Fig. 8g, will result in a staggered assembly of an array of fiber-embedded Cu-Si Schottky diodes.

As mentioned, a different “interference” approach is proposed for the devices’ assembly in multiple cores with identical γ, such as Si pn-diodes, as described and analyzed in Fig. 9. Investigating a breakup in a double core fiber in Fig. 9a, in which the two cores with $2{a}_{1}=0.7\mu m$, $2{a}_{2}=2\mu m$ were separated by a center-to-center gap of $\Gamma=4.8\mu m$, we have found that, unlike in fiber with identical cores^7^ (see Fig. 1d), each of the cores breaks up according to its individual wavevector ${{{{{{\bf{k}}}}}}}_{{{{{{\bf{1}}}}}},{{{{{\bf{2}}}}}}}\equiv \frac{1}{{\left.\lambda \right|}_{{x}_{{bu}}}^{{{{{\mathrm{1,2}}}}}}}$, and only occasionally the spheres from the neighboring cores come in contact with each other at locations of constructive interference between ${{{{{{\bf{k}}}}}}}_{{{{{{\bf{1}}}}}}}$ and ${{{{{{\bf{k}}}}}}}_{{{{{{\bf{2}}}}}}}$, with the beat wavevector of such an interference ${{{{{\bf{K}}}}}}=$ ${{{{{{\bf{k}}}}}}}_{{{{{{\bf{1}}}}}}}{{{{-}}}}{{{{{{\bf{k}}}}}}}_{{{{{{\bf{2}}}}}}}$, as is depicted schematically in Fig. 9b. This in-and-out-of-phase beat is evident at all feed speeds, as can be seen in Fig. 9c, showing the breakup of the double core from Fig. 9a in the flame resulting from flow rates of ${H}_{2}{@}0.45{l}/\min$ and ${O}_{2}{@}0.4{l}/\min$ at varying feed speed. The last is true, with the exception of ${v}_{f}=2{\mu m}/s$. At such a slow feed speed, the thin core broke up chaotically, likely due to the violation of VL2, i.e., ${v}_{f} \, \ll \, {v}_{f}^{{VL}2}\left({a}_{1},{T}(x)\right)$.

Attempts at fitting AVG-IM to the results in Fig. 9c revealed that no temperature profile corresponds to the results observed for both cores concurrently. Figure 9d, demonstrates the best AVG-IM fits resulting from the double-exponential temperature profiles ${T}_{i}={T}_{\max }^{i}-({T}_{\max }^{i}-{{T}}_{{Si}})(C{e}^{-x/{w}_{1}}+(1-C){e}^{-x/{w}_{2}})$, where $i=\left\{{{{{\mathrm{1,2}}}}}\right\}$ are the core indices. The best fits to the thinner ($2{a}_{1}=0.7\mu m$) and thicker ($2{a}_{2}=2\mu m$) core results yield ${T}_{\max }^{1}=2470{K}$ and ${T}_{\max }^{2}=2590{K}$, respectively. ${w}_{1}=4150\mu m$, ${w}_{2}=65\mu m$, $C=0.33$ for both temperature profiles. It is apparent from Fig. 9d that the temperature profile ${T}_{2}$, fitting well the thick core breakups, yields AVG-IM extrapolation that significantly undershoots those of the thinner core, and vice versa, the temperature profile ${T}_{1}$, fitting well the thin core breakup periods, yields extrapolation of AVG-IM to the thick core that significantly overshoots the experimental results, with one notable exception—for the period resulting from ${v}_{f}=2{\mu m}/s$, which shoots up closer to AVG-IM prediction for ${T}_{1}$.

This last exception, in combination with AVG-IM analysis, suggests that when the breakup in both cores is periodic, the instabilities developing in each core are mechanically coupled, drawing the breakup periods much closer together than would be expected for independent, decoupled breakups, with this coupling lost when one of the breakups falls into the random regime. When both cores are breaking up predictably, the periods are long enough for the sum of the sphere radii, which collect into them the core material of the entire period, to exceed the separation between the cores, i.e., ${a}_{1}+{a}_{2} > \Gamma$, which makes the spheres touch and fuse into bi-spherical devices when in phase. Physical contact is a blunt sign of mechanical coupling. On the contrary, when one of the cores breaks up randomly, its breakup period shortens. Due to the volume conservation argument, the spheres become much smaller and might lose that proximity to the spheres from the neighboring periodically breaking up core. Additionally, momentum conservation should hold like for any other mechanical system. In other words, breakups must have similar wavevectors, ${{{{{{\bf{k}}}}}}}_{{{{{{\bf{1}}}}}}}\,{{{{\approx }}}}\,{{{{{{\bf{k}}}}}}}_{{{{{{\bf{2}}}}}}}$, to exchange energy efficiently, while ${{{{{{\bf{k}}}}}}}_{{{{{{\bf{1}}}}}}}\,{{{{\gg }}}}\,{{{{{{\bf{k}}}}}}}_{{{{{{\bf{2}}}}}}}$ when one of the cores breaks up randomly.

Since we lack a quantifiable understanding of the coupling mechanism, we can’t predict whether the breakup periods will eventually get synchronized^49^. We can say, though, that in fibers with submicron core cross-sections (Fig. 9e–k), the coupled breakup continues for tens of beat periods $1/{{{{{\bf{K}}}}}}$ without signs of decay, resulting in 3-4 diode assemblies per bit period. Figure 9e–k analyzes the breakup at ${v}_{f}=10\mu m/s$, in the flame of ${H}_{2}=0.17{l}/\min$ and ${O}_{2}=0.08{l}/\min$, of the double-core fiber resulting from scaling down of the fiber in Fig. 9a to the cladding thickness of 25 µm (see Methods) such that the cores enter the submicron cross-section regime.

It is important to note that in thermal draw and breakup, a shell comprised of Si-silica nanocrystal mixture covering a sphere is formed, resulting from the chemical reaction occurring at the Si-silica interface at elevated temperatures. This shell of the third material (neither Si, nor silica) forms a mechanically isolating but electrically conducting membrane on the spheres. The presence of that shell is what allows the p- and n-doped Si droplets to come in contact, while maintaining the junction, without merging into a uniformly doped single droplet. The SEM analysis of submicron spheres (Fig. 9h) reveals the surface membrane porosity when the silica component is etched out. Augmenting the SEM, TEM (Fig. 9i) shows the core-shell structure of the sphere. Figure 9j–k shows that the spheres in the assemblies are physically fused without merging. For the details of the membrane formation and function, please refer to Ref. ^7^. All the exposed spheres, sphere assemblies, and continuous core sections in Fig. 9e–o were obtained by etching the silica cladding in hydrofluoric acid overnight and evaporating the residual acid (for details, see ref. ^7^).

The breakup behavior in the scaled-down fiber in Fig. 9e cannot be analyzed using AVG-IM since it’s happening deep in the regime, violating VL2 for both cores. From Eq. (8), ${v}_{f}^{{VL}2}\left(a,{T}(x)\right)$ is inversely proportional to $a$. In the scaled-down fibers, the cores are scaled down to $2{a}_{1}\approx 60{nm}$ and $2{a}_{2}\approx 180{nm}$, meaning that both cores are deeper into the VL2-violating regimen than even the $2a=500{nm}$ sample in Fig. 8a, while produced at an identical feed speed.

Experimentally, we know that a submicron core of a single-core fiber (Fig. 1e) breaking up at identical conditions shows a clear Tomioka-like concurrent development of multiple instability periods in a long section of a core, if quenched before entering the flame completely, as can be seen in Fig. 9l. At the same time, an inspection of a comparable, halted-before-complete-breakup section of the core in the predictable AVG regime clearly shows a formation of one discrete droplet at the core tip, as is demonstrated in Fig. 9m–o.

It is essential to mention that the periodicity of the submicron breakup doesn’t make it predictable, at least not in the AVG-IM sense. Based on qualitative experimental observations (see Methods), we suggest that the submicron breakup is chaotic, yet with the reduction of the flame temperature concentrates towards a so-called fixed-point attractor, a well-concentrated preferred region in the otherwise chaotic phase space, resulting in a statistically predictable monodisperse breakup^14,21,22^.

In fact, complex systems are rarely non-statistically predictable. In that sense, AVG-IM, obeying the linear Euler-Lagrange dynamics, thus mapping the user-engineered spatiotemporal conditions into the system dynamics in an exact, one-to-one, and not just probabilistic fashion^50–52^, is a rare instance of “finding order in chaos.” Capable of heuristically decomposing a continuous spectrum of capillary instabilities into single-phase single-wavelength components, we believe that the multimaterial fiber in combination with AVG-IM can be used to physically simulate the otherwise computationally heavy (see Supplementary Note 7) Navier-Stokes equations of viscous multimaterial flow.

## Methods

### Fiber Draw

The silicon-core silica fiber in Fig. 3 was drawn using a draw tower (Optogear OG-510D) in two steps. For the first step, a five cm-long, two mm-thick Silicon rod, 5 N purity (ESPI Metals) was sleeved in concentric silica tubes (Technical Glass Products) (see Supplementary Note 8) and sealed under vacuum using a glass lathe (Optogear OG-440), resulting in a preform $25{mm}$ in diameter, which drawn at a temperature of $1950\,{\,\!}^{\circ}{{{{{\rm{C}}}}}}$, feed speed of $1.28{mm}/\min$ and draws speed of $0.2{m}/\min$. The resulting cane was $2{mm}$ in diameter, with a core 160 $\mu m$ in diameter (Supplementary Note 8). It was re-sleeved in silica tubes and sealed under vacuum in the glass lathe to produce a preform, $25{mm}$ in diameter. This new preform was then drawn at $2000\,{\,\!}^{\circ}{{{{{\rm{C}}}}}}$, with a feed speed of $6.25{mm}/\min$ and draw speed of $10{m}/\min$, to obtain the final 280 $\mu m$-thick fiber, with a $4\mu m$-thick Si core (see Supplementary Note 8). The conceptually similar fabrication procedure, which yielded an architecturally identical fiber in Figs. 1c and 6 is described in ref. ^7^. The Si core fiber samples in Supplementary Note 9 with the $1.5{\mu m}$ core and those in Figs. 1g and 8a with $0.5{\mu m}$ core originate from the same draw as the samples in Figs. 1c and 6, taken from the fiber section resulting from the draw stage where the Si-containing section of the preform enters the draw process, resulting in the adiabatic taper-in of the core over a few meters ($\sim 5{m}$), eventually stabilizing at a steady state for multiple tens of meters ($\sim 60{m}$) at the $4{\mu m}$ core thickness.

The procedure for the fabrication of the double-core fibers in Figs. 1d, 1f, 9a, and 9c is similar to the one in ref. ^7^. First, a preform containing two separate Si rods was prepared and drawn into a double-core fiber. One *p*-type Si rod (electron acceptors concentration ${N}_{A}=6.9\times {10}^{18}{{cm}}^{-3}$) and one *n*-type Si rod (electron donors concentration ${N}_{D}=1.6\times {10}^{19}\,{{cm}}^{-3}$) with a diameter of $2{mm}$ were sleeved into a preform with a silica cladding of $12{mm}$ and drawn at $2130\,{\,\!}^{\circ}{{{{{\rm{C}}}}}}$ with the feed speed ${v}_{f}=0.6{mm}/\min$ and draw speed of up to ${v}_{d}=0.6{m}/\min$. Then one of the sections, $11{cm}$ long, of the resulting cane with an outer cladding diameter of $630{\mu m}$ and containing two cores, each 110 µm thick separated by $200{\mu m}$ center-to-center gap, was incorporated into a new $12{mm}$ thick preform. That preform was then drawn with the feed speed ${v}_{f}=1.2{mm}/\min$ and the draw speed of ${v}_{d}=2{m}/\min$ at the draw temperature of 2085 °C. This resulted in tens of meters of fiber with an outer cladding diameter of $280{\mu m}$, with the pair of cores, the first with the thickness fluctuating slowly (on the scale of meters) around the average of $1.5{\mu m}$ with the standard deviation of $0.4{\mu m}$ in the range of $0.7$ to $2.0{\mu m}$, and the second with the thickness fluctuating around 1.8 µm with the standard deviation of 0.4 µm in the range of $1.2$ to $2.4{\mu m}$. The center-to-center separation between the two averaged at 5.0 µm with the standard deviation of $0.3{\mu m}$ in the range of $4.6$ to $5.4{\mu m}$.

The Cu-core silica fibers used for the study in Fig. 8 were produced by a redraw of $12{cm}$ long 0.4*mm* thick silica cane with $90{\mu m}$ Cu core sleaved and vacuum sealed into a concentric assembly of silica tubes of internal diameter of $0.5{mm}$ and external diameter of $13{mm}$. The cane redraw was performed at $1.1{mm}/\min$ preform feed speed into the furnace and maximal fiber drawing capstan speed of $2{m}/\min$, in $2070\,{\,\!}^{\circ}{{{{{\rm{C}}}}}}$ to $2090\,{\,\!}^{\circ}{{{{{\rm{C}}}}}}$. The redraw process resulted in tens of meters $( \sim 15{m})$ of fiber with a copper core of a thickness ranging from $0.9$ to $7{\mu m}$. The cane used for the redraw resulted from a draw of $0.8{mm}$ copper wire vacuum sealed in silica preform with an internal diameter of $1{mm}$ and external diameter of $13{mm}$. The draw of the cane was performed at $2100\,{\,\!}^{\circ}{{{{{\rm{C}}}}}}$ furnace temperature, at $1.2{mm}/\min$ preform feed speed into the furnace, and maximal fiber drawing capstan speed of $1.8{m}/\min$.

### AVG capillary breakup experiments

We performed the breakup experiments using a hydrogen-oxygen torch as a source of heat. Flows of each of the gases, ${H}_{2}$ and ${O}_{2}$, were controlled by two individual Omega FMA5400A_5500A digital mass-flow controllers calibrated to nitrogen. The mixture of burning gases were ejected through an orifice of a torch (National Torches Model 3H) $0.02\hbox{''}$ in diameter (MSOX-0 mini tip) to form the flame. The distance between the torch orifice and the fiber was 12 mm in the etalon experiment in Fig. 6 but was adjusted up or down for lower and higher flames, respectively, for other experiments, wherever appropriate, such that the fiber is set at the tip of the flame, where the heat deposition is the most efficient. The feed speed ranged from $2\mu m/s$ to $150\mu m/s$. A camera (Amscope MU900 (Fig. 3)/Motic Moticam1000 (Fig. 6)) registered the breakup events. The movies collected were analyzed frame-by-frame to capture each individual droplet breakup. The breakup location was measured with respect to the melting position of the silicon core, which is identified as a dip in the captured image intensity (see Supplementary Fig. 4 in Supplementary Note 4). The breakup period was calculated by multiplying the feed speed and the time between consecutive breakups, as well as measured by optical inspection of the fibers post-breakup.

### Ab-initio simulations

The Navier-Stokes ab-initio simulations were performed in Indiana University’s Big Red 3 and Big Red 200 supercomputers. The output was verified against the outputs obtained from running it at the jdj cluster at the Massachusetts Institute of Technology, where it was previously developed and ran for other studies^30,31,33^ Significant speedup was achieved without a change in outcome (Supplementary Note 7).

### Proof of equivalence of AVG-IM to a description of the system in terms of Euler-Lagrange mechanics

Equation (2) in its rearranged form, as Eq. (4), becomes simply $L\equiv {{{{{\rm{PE}}}}}}-{{{{{\rm{KE}}}}}}=0$.

The Lagrange equation of motion $\frac{d}{{dt}}\left(\frac{\partial L}{\partial \dot{q}}\right)-\frac{\partial L}{\partial q}=0$ can be written as ${\left.\frac{d}{{dt}}\left(a\beta {\eta }_{{clad}}\right)\right|}_{{x}_{{bu}}}+\gamma=0$, taking $q=\lambda$, and noting that $\dot{q}={v}_{f}$, since that’s the speed at which the length of the reshaping core section fed through the front ${x}_{{bu}}$ grows (Fig. 2c). Additionally, noting that $\frac{d}{{dt}}=\frac{{dx}}{{dt}}\frac{d}{{dx}}={v}_{f}\frac{d}{{dx}}$, we arrive at ${\left.\frac{d{\eta }_{{clad}}}{{dx}}\right|}_{{x}_{{bu}}}=-\frac{\gamma }{a\beta {v}_{f}}$, which from Eq. (2) equals to ${\left.-\frac{{\eta }_{{clad}}}{\lambda }\right|}_{{x}_{{bu}}}$. Since ${\eta }_{{clad}}$ is a function of $x$ only, the equation of motion can now be rewritten as ${\left.\lambda \right|}_{{x}_{{bu}}}={\left.\left(-\frac{{\eta }_{{clad}}\left(x\right)}{\partial {\eta }_{{clad}}\left(x\right)/\partial x}\right)\right|}_{{x}_{{bu}}}$, which is Eq. (3) in its explicit form. To see that, we can drop the constants and take the derivatives, after which Eq. (3) takes the form of ${\left.\left(\frac{\partial }{\partial x}-\frac{\partial }{\partial \lambda }\right)\left(\frac{{\eta }_{{clad}}(x)}{\lambda }\right)\right|}_{{x}_{{bu}}}=\frac{1}{\lambda }\cdot {\left.\left(\frac{\partial {\eta }_{{clad}}(x)}{\partial x}+\frac{{\eta }_{{clad}}(x)}{\lambda }\right)\right|}_{{x}_{{bu}}}=0$ that rearranges into ${\left.\lambda \right|}_{{x}_{{bu}}}={\left.\left(-\frac{{\eta }_{{clad}}\left(x\right)}{\partial {\eta }_{{clad}}\left(x\right)/\partial x}\right)\right|}_{{x}_{{bu}}}$

QED∴

### Assertation of the validity limitations of the AVG-IM

VL1: The model is valid only if the breakup period is comparable to the width of the hot-zone boundary: From Eq. (3) in the form ${\left.\lambda \right|}_{{x}_{{bu}}}={\left.\left(-\frac{{\eta }_{{clad}}\left(x\right)}{\partial {\eta }_{{clad}}\left(x\right)/\partial x}\right)\right|}_{{x}_{{bu}}}$, we see that the model is invalid for uniform-viscosity threads. If ${\eta }_{{clad}}\left(x\right)={const}$, it follows that $\partial {\eta }_{{clad}}(x)/\partial x=0$, and ${\left.\lambda \right|}_{{x}_{{bu}}}=\pm \infty$ is not physical. If, additionally, we approximate viscosity and viscosity gradient by average values within the hot-zone boundary, i.e., ${\eta }_{{clad}}\left(x\right)\,\approx \,({\eta }_{\max }-{\eta }_{\min })/2$ and $\partial {\eta }_{{clad}}(x)/\partial x\,\approx \,({\eta }_{\max }-{\eta }_{\min })/w$, we conclude that ${\left.\lambda \right|}_{{x}_{{bu}}}\approx w$. In other words, AVG-IM is valid for breakup periods comparable to the width of the liquified region boundary. If $w=0$, such as in dripping from an orifice, ${\left.\lambda \right|}_{{x}_{{bu}}}=0$ is not physical.

VL2: The model is valid only if the pinch location falls within the hot-zone boundary: From Eq. (2) it follows that $\lambda \sim {v}_{f}$. Thus if ${v}_{f}=0$, ${\left.\lambda \right|}_{{x}_{{bu}}}=0$ is not physical. Additionally, if ${v}_{f}$ is too large, the thread will reach the asymptotic temperature ${T}_{\max }$ before the pinch-off. The breakup then happens at uniform viscosity conditions, where the model doesn’t hold (see (VL1)). In other words, our model is valid if the pinch-off location is within the liquified region boundary, meaning that ${t}_{{pinch}}\approx w/{v}_{f}$.

### Fiber scaling

The fiber in Fig. 9e resulted from scaling down the double-core fiber in Fig. 9a (275 $\mu m$-thick silica cladding with two Si cores, 2.0 and 0.7 $\mu m$-thick, separated by 4.8 $\mu m$ center-to-center) in three steps according to the procedure described in Supplementary Note 9 and ref. ^7^. The fiber diameter reduction by a factor of $\sqrt{5}$ in each step resulted from ${v}_{f}=50\mu m/\sec$ and ${v}_{d}=250\mu m/\sec$. The flame used as a heat source for the reduction procedure resulted from H_2_ flow of 0.25 l/m, 0.22 l/min, and 0.20 l/min for the first, second, and third scaling-down repetitions, respectively.

### Extending AVG-IM to the general viscosity contrast regime

We suggest that AVG-IM will hold true for the general core-cladding viscosity contrast $\frac{{\eta }_{{core}}}{{\eta }_{{clad}}}$, if ${{{{{\rm{Ca}}}}}}$ in Eqs. (2) and (3) is replaced with ${{{{{{\rm{Ca}}}}}}}^{\frac{{\eta }_{{core}}}{{\eta }_{{clad}}}}(\lambda,x)\equiv \beta \cdot {v}_{f}/\left[2\lambda \cdot {{{{{\rm{in}}}}}}\left(\lambda,{\eta }_{{clad}}(x),{\eta }_{{core}}(x)\right)\right]$, where ${{{{{\rm{in}}}}}}\left({{{{\lambda }}}},{{{{{\eta }}}}}_{{{{{clad}}}}},{{{{{\eta }}}}}_{{{{{core}}}}}\right)$ is the Tomotika instability rate.

To justify this educated guess, let us look at ${{{{{\rm{in}}}}}}\left(\lambda,{\eta }_{{clad}},{\eta }_{{core}}\right)$, as is defined in Eq. (38) in ref. ^23^: ${{{{{\rm{in}}}}}}\left(\lambda,{\eta }_{{clad}},{\eta }_{{core}}\right)=(\gamma /2a{\eta }_{{clad}})\cdot F$, where $F=F(a/\lambda,{\eta }_{{core}}/{\eta }_{{clad}})$ is a function of the core radius normalized to the instability wavelength ${{{{a}}}}/{{{{\lambda }}}},$ and the viscosity contrast ${{{{{{\rm{\eta }}}}}}}_{{{{{{\rm{core}}}}}}}/{{{{{{\rm{\eta }}}}}}}_{{{{{{\rm{clad}}}}}}}$. As is demonstrated in Table II in ref. ^23^, $F\to 1$ when ${\eta }_{{core}}/{\eta }_{{clad}}\to 0$ and $a/{\left.\lambda \right|}_{{x}_{{bu}}}\to 0$. In the Tomotika model, ${\left.{{{{\lambda }}}}\right|}_{{{{x}}}_{{{bu}}}}$ is called “the dominant wavelength”. It is the wavelength that maximizes the instability rate spectrum ${{{{{\rm{in}}}}}}\left(\lambda,{\eta }_{{clad}},{\eta }_{{core}}\right)$, and thus develops towards pinching faster than any other wavelength ${{{{\lambda }}}}$ in the spectrum.

In silica-based optoelectronic and photonic fibers, heuristically, ${{{{{{\rm{\eta }}}}}}}_{{{{{{\rm{core}}}}}}}({{{{{\rm{x}}}}}} \, > \, 0) \, \ll \, {{{{{{\rm{\eta }}}}}}}_{{{{{{\rm{clad}}}}}}}({{{{{\rm{x}}}}}} \, > \, 0)$, and any practical ${\left.\lambda \right|}_{{x}_{{bu}}}$ is of the order of tens to hundreds of microns, while $a$ is micrometric or submicrometric; thus $a \, \ll \, {\left.\lambda \right|}_{{x}_{{bu}}}$, resulting in $F \, \approx \, 1$ and ${{{{{\rm{in}}}}}}\left(\lambda,{\eta }_{{clad}},{\eta }_{{core}}\right)\,\approx \,\gamma /2a{\eta }_{{clad}}$ for ${{{{\lambda }}}}$ in the vicinity of ${\left.{{{{\lambda }}}}\right|}_{{{{x}}}_{{{bu}}}}$. Thus, in the limit where the simplification MS2 holds, for ${{{{\lambda }}}}$ in the vicinity of ${\left.\lambda \right|}_{{x}_{{bu}}}$, ${{{{{{\rm{Ca}}}}}}}^{\frac{{\eta }_{{core}}}{{\eta }_{{clad}}}}(\lambda,x)$ converges towards ${{{{{\rm{Ca}}}}}}(\lambda,x)=\beta a{\eta }_{{clad}}(x){v}_{f}/(\gamma \lambda )$ defined in Eq. (1).

Convergence of ${{{{{{\rm{Ca}}}}}}}^{\frac{{\eta }_{{core}}}{{\eta }_{{clad}}}}(\lambda,x)$ towards ${{{{{\rm{Ca}}}}}}\left(\lambda,x\right)$ for ${\eta }_{{core}}/{\eta }_{{clad}}\to 0$ supports our educated guess that extrapolates the AVG-IM into to the general viscosity contrast.

### Comparison of the methods for measuring the interface energy: sessile-drop method vs. fiber-AVG-IM method

Sessile-drop interface energy measurement of an arbitrary melt $M$ with silica, ${\gamma }_{{{SiO}}_{2}-M}$, shown in Fig. 8e entails placing a droplet of melt $M$ on a planar SiO_2_ substrate, measuring the contact angle ${\theta }_{C}$ that the drop of melt forms with the SiO_2_ surface, and calculating ${\gamma }_{{{SiO}}_{2}-M}$ according to

$${\gamma }_{{{SiO}}_{2}-M}={\gamma }_{{{SiO}}_{2}-{air}}-{\gamma }_{M-{air}}\cdot \cos {\theta }_{C}$$

while the silica-air interface energy ${\gamma }_{{{SiO}}_{2}-{air}}$ and melt-air interface energy ${\gamma }_{M-{air}}$ must be known from elsewhere.

To compare, the conventional sessile-drop method in Fig. 8e measures interface energy between solid substrate and melt using two reference interface energies, while the fiber-AVG-IM method in Fig. 8f measures interface energy between two melts using one reference interface energy. For the measurement of ${\gamma }_{{{SiO}}_{2}-{Cu}}$ we use silica-silicon interface energy ${\gamma }_{{{SiO}}_{2}-{Si}}=0.30{J}\cdot {m}^{-2}$ as a reference.

Each additional reference is a potential source of random error. Literature value for ${\gamma }_{{{SiO}}_{2}-{Cu}}$ in ref. ^48^ is $1.04{J}\cdot {m}^{-2}$, a 13% lower than the value we found in our study. Yet, ${\gamma }_{{{SiO}}_{2}-{Si}}$ found in ref. ^48^ is $0.24{J}\cdot {m}^{-2}$ which is 20% lower than the value we use in this study. The discrepancy might arise primarily from the discrepancy in the reference values.

More so, in the sessile-drop method, to accurately measure the contact angle, the substrate must stay planar and thus ***solid*** throughout the measurement, which might be challenging while in contact with the high-temperature melt. Additionally, openness to the ambient atmosphere frequently complicates temperature control. It might introduce unwanted chemical reactions affecting the conclusiveness of the sessile-drop method. At the same time, in the fiber-AVG-IM method, the measured melt can be fully encased in a thermally and chemically isolating cladding.

### Guiding principles for the choice of ${{{{{{\boldsymbol{T}}}}}}}_{{{{{{\bf{1}}}}}}}{{{{{\boldsymbol{(}}}}}}{{{{{\boldsymbol{x}}}}}}{{{{{\boldsymbol{)}}}}}}$ and ${{{{{{\boldsymbol{T}}}}}}}_{{{{{{\bf{2}}}}}}}{{{{{\boldsymbol{(}}}}}}{{{{{\boldsymbol{x}}}}}}{{{{{\boldsymbol{)}}}}}}$ in Fig. 7d

${T}_{1}(x)$ was constructed such that it reaches the softening point of silica (~1900 K) very rapidly, significantly faster than ${T}_{2}\left(x\right)$, but attains ${T}_{\max }$ much slower than it, with the intention to examine the “globality” vs. “locality” of the nature of the predictable AVG capillary instability. On the one hand, to solve Navier-Stokes equations numerically, one must define the viscosities of materials globally, i.e., everywhere in the solution domain, while AVG-IM, in the way its formulated, if fully defined by the local viscosities around the pinch-off location. In the former approach, the viscosities of melts are important everywhere in the solutions domain and are all considered to contribute to the development of the instability, while in the latter—only the viscosities in the vicinity of the actual pinch-off count. So, which view of the two is more accurate?

A comparison of the breakup resulting from the two profiles reveals that the local scenario is more accurate, not only for AVG-IM, but also for ab-initio, which potentially simplifies the numerical calculations and thus shrinks otherwise very extended calculation times.

### AVG-IM application to the Si- and Cu-core breakup in a pure hydrogen flame

AVG-IM fitting to the set of the three Si-core data points collected for ${H}_{2}=1.2l/\min$, shown in Fig. 8a, yields a temperature profile according to Eq. (6) with ${T}_{{{\rm{max }}}}={2185}K$ and $w=1275\mu m$.

To analyze the results for the Cu-core breakups in Fig. 8b, we note (see Supplementary Note 10) that the gas flow of ${H}_{2}=0.4{l}/\min$ results in geometrically similar but overall colder temperature profile than that of ${H}_{2}=1.2{l}/\min$. Thus, to find the temperature profile for ${H}_{2}=0.4{l}/\min$ we fix $w$ at $1275\mu m$, found for ${H}_{2}=1.2{l}/\min$ data, and lower the ${T}_{\max }$, until the best fit is obtained to the only Si data point that we have for the same temperature conditions as the Cu-core breakups we want to analyze. It is the $2a=4\mu m$ Si-core data point in Fig. 8b. This results in the temperature profile with ${T}_{\max }=2065{K}$ for ${H}_{2}=0.4{l}/\min$.

### Assessment of the validity limits of AVG-IM based in Si-core breakup experiments

Worth mentioning is the set of conditions that resulted in the random breakup (see Supplementary Note 10, Fig. 1g), and thus cannot be represented as a point in Fig. 8a:

${\left.\lambda \right|}_{{x}_{{bu}}}={random}$: Si-core, H_2_ flow rate = 1.2 l/min, $2a=0.5\mu m$, and ${v}_{f}=10\mu m/s$.

In combination with another set of conditions in Fig. 8a, yielding a predictable breakup,

${\left.\lambda \right|}_{{x}_{{bu}}}=910\pm 25\mu m$: Si-core, H_2_ flow rate = 1.2 l/min, $2a=0.5\mu m$, and ${v}_{f}=50\mu m/s$, it allows us to access the validity-limitation of AVG-IM experimentally, violated when the feed speed becomes too slow. Specifically, note that the AVG-IM validity limitation VL2, written as ${x}_{{bu}} \sim {v}_{f}\cdot {t}_{{pinch}} \, \approx \, {{{{w}}}}$, defines a characteristic feed speed,

$${v}_{f}^{{VL}2}\left(a,T(x)\right)\equiv \frac{{{{{w}}}}}{{t}_{{pinch}}}=w\cdot \frac{\gamma }{\beta a{\left.\eta \left(x\right)\right|}_{{x}_{{bu}}=w}}$$

which is the feed speed that, for a given temperature profile and core radius, results in the pinch location ${x}_{{bu}}$ occurring exactly the distance of the boundary width $w$ into the liquefaction zone. It defines the feed speed at which the breakup is safely predictable. If the feed speed is much faster than ${v}_{f}^{{VL}2}$, the core is expected to reach the uniform temperature too soon and fall into a Tomotika-like jetting regime. Yet surprisingly, we find experimentally that the opposite is also true: the feed speed much slower than ${v}_{f}^{{VL}2}$ results in the core not experiencing a viscosity gradient high enough by the time of the breakup, and thus also falling into an effectively Tomotika-like jetting regime.

Once the temperature profile for ${H}_{2}=1.2l/\min$ is obtained from AVG-IM fitting, we calculate ${v}_{f}^{{VL}2}=67\mu m/s$ for $2a=0.5\mu m$. Knowing that ${v}_{f}=50\mu m/s$ yields a predictable breakup for that core radius, while for ${v}_{f}=10\mu m/s$ the breakup is random, we assess that feed speeds equal or larger than 75% of ${{{{{v}}}}}_{{{{{f}}}}}^{{{{{VL}}}}2}$ fall safely in the predictable breakup range, while feed speeds equal or smaller than 15% of ${{{{{v}}}}}_{{{{{f}}}}}^{{{{{VL}}}}2}$ fall safely out of the predictable breakup range. For comparison, calculating ${{{{{v}}}}}_{{{{{f}}}}}^{{{{{VL}}}}2}=8{{{{\mu }}}}{{{{m}}}}/s$ for $2{{{{a}}}}=4{{{{\mu }}}}{{{{m}}}}$ for the same temperature profile, we verify that for the same feed speed ${v}_{f}=10\mu m/s$ that results in the random breakup in the core of $2a=0.5\mu m$, the breakup is indeed expected to be predictable, in alignment with the findings in Fig. 8a, since ${v}_{f}=1.25\cdot {v}_{f}^{{VL}2}$ in that case, high enough not to fall into an unpredictable range. For $2a=4\mu m$ 75% of ${v}_{f}^{{VL}2}$ corresponds to ${v}_{f}=6\mu m/s$ and 15% of ${v}_{f}^{{VL}2}=1.2\mu m/s$, the last of which is even below the slowest feed speed ${v}_{f}^{{{\rm{min }}}}=2\mu m/s$ attainable by our experimental setup (see Supplementary Note 10). It is no wonder, thus, that we have never observed random breakup in our etalon fiber.

### Fixed attractor in the chaotic phase space of a submicron core breakup

We qualitatively observe that once the core thickness $2a$ is small enough and the feed speed ${v}_{f}$ is slow enough for the breakup to fall into the random regime ${v}_{f} \, \ll \, {v}_{f}^{{VL}2}\left(a,{T}(x)\right)$, the instability becomes more and more periodic as the temperature of the liquefaction zone ${T}_{\max }$ is reduced. For comparison, a random breakup in Fig. 1g results from a relatively hot flame $({H}_{2}=1.2{l}/\min)$, while that in Fig. 1e from a cold one (${H}_{2}=0.18{l}/\min; {O}_{2}=0.07{l}/\min$). The second flame is an extreme case in which the heat deposited to the fiber by the flame is marginally sufficient to liquify the cladding. When ${H}_{2}$ flow in this flame was reduced by as small as $0.01{l}/\min$, the breakup in the core would stop occurring, and the core would pass the flame intact. In these marginally-liquefying conditions, the breakup becomes strictly periodic, approaching Plateau–Rayleigh limit of $k=1/2\pi a$, which for submicron $a$ is technologically useful for the production of photonic gratings and optical metamaterials in fibers^7^.

Though, the periodicity of the submicron breakup doesn’t make it predictable, at least not in the AVG-IM sense. We suggest that the submicron breakup is chaotic, yet with the reduction of the flame temperature concentrates towards a so-called fixed-point attractor, a well-concentrated preferred region in the otherwise chaotic phase space, resulting in a statistically monodisperse breakup^14,21,22^.

### Supplementary information

Supplementary Information

Peer Review File

Description of Additional Supplementary Files

Supplementary Movie 1

Supplementary Movie 2

## Data Availability

The breakup data and its analysis generated in this study have been deposited online^53^. The additional raw breakup data generated in the course of this study in support of its conclusions is provided in the Supplementary Information.

## Code availability

The Navier-Stokes equations ab-initio simulation code, the code for the analysis of the ab-initio simulations output, and the scripts for generating the AVG-IM analysis used in this study are available through the Zenodo repository^53^.

## References

[1] Tao, G., Abouraddy, A. F., Stolyarov, A. M. & Fink, Y. Multimaterial Fibers. In *Lab-on-Fiber Technology*, Part of the book series: Springer Series in Surface Sciences (SSSUR) Vol. 56, 1–26 (Springer, 2015).

[2] Yan, W. et al. Thermally drawn advanced functional fibers: New frontier of flexible electronics. *Materials Today* **35**, 168–194 (2020).

[3] *Fiber Sensing: Medical fiber-optic sensors offer haptics, 3D shape sensing, and pressure sensing | Laser Focus World*. https://www.laserfocusworld.com/fiber-optics/article/16546889/fiber-sensing-medical-fiberoptic-sensors-offer-haptics-3d-shape-sensing-and-pressure-sensing.

[4] Sorin F (2007). Multimaterial Photodetecting Fibers: a Geometric and Structural Study. Adv. Mater..

[5] Gumennik A (2012). All-in-Fiber Chemical Sensing. Adv. Mater..

[6] Gumennik A (2017). Confined in-fiber solidification and structural control of silicon and silicon−germanium microparticles. PNAS.

[7] Gumennik A (2013). Silicon-in-silica spheres via axial thermal gradient in-fibre capillary instabilities. Nat. Commun..

[8] Rein M (2016). Self-assembled fibre optoelectronics with discrete translational symmetry. Nat. Commun..

[9] Wei L (2017). Optoelectronic Fibers via Selective Amplification of In-Fiber Capillary Instabilities. Adv. Mater..

[10] Zhang Y, Zhao Y, Ren J, Weng W, Peng H (2016). Advances in Wearable Fiber-Shaped Lithium-Ion Batteries. Adv. Mater..

[11] Dong K, Peng X, Wang ZL (2020). Fiber/Fabric-Based Piezoelectric and Triboelectric Nanogenerators for Flexible/Stretchable and Wearable Electronics and Artificial Intelligence. Adv. Mater..

[12] Rein M (2018). Diode fibres for fabric-based optical communications. Nature.

[13] Loke G (2021). Digital electronics in fibres enable fabric-based machine-learning inference. Nat. Commun..

[14] Dreyer K, Hickey FR (1991). The route to chaos in a dripping water faucet. Am. J. Phys..

[15] Xu B (2019). Filament formation via the instability of a stretching viscous sheet: Physical mechanism, linear theory, and fiber applications. Phys. Rev. Fluids.

[16] Xu B, Deng D (2020). Linear analysis of dewetting instability in multilayer planar sheets for composite nanostructures. Phys. Rev. Fluids.

[17] Kaufman JJ (2012). Structured spheres generated by an in-fibre fluid instability. Nature.

[18] Fokine M (2017). Laser structuring, stress modification and Bragg grating inscription in silicon-core glass fibers. Opt. Mater. Express.

[19] Zhang J (2017). Laser-Induced In-Fiber Fluid Dynamical Instabilities for Precise and Scalable Fabrication of Spherical Particles. Adv. Funct. Mater..

[20] Sciamanna M, Shore KA (2015). Physics and applications of laser diode chaos. Nat. Photonics.

[21] Ambravaneswaran B, Phillips SD, Basaran OA (2000). Theoretical Analysis of a Dripping Faucet. Phys. Rev. Lett..

[22] Cahalan RF, Leidecker H, Cahalan GD (1990). Chaotic Rhythms of a Dripping Faucet. Comput. Phys..

[23] Tomotika S (1935). On the Instability of a Cylindrical Thread of a Viscous Liquid Surrounded by Another Viscous Fluid. Proc. R. Soc. Lond. A.

[24] Powers TR, Zhang D, Goldstein RE, Stone HA (1998). Propagation of a topological transition: The Rayleigh instability. Phys. Fluids.

[25] van Saarloos W (2003). Front propagation into unstable states. Phys. Rep..

[26] Faccini de Lima C (2019). Towards Digital Manufacturing of Smart Multimaterial Fibers. Nanoscale Res. Lett..

[27] Zhang J, Wang Z, Wang Z, Zhang T, Wei L (2019). In-fibre particle manipulation and device assembly via laser induced thermocapillary convection. Nat. Commun..

[28] van der Elst L (2021). 3D Printing in Fiber-Device Technology. Adv. Fiber Mater..

[29] Faccini de Lima, C., Leffel, T., Zheng, M., Coulter, J. R. & Gumennik, A. Creating fiber-embedded photonic circuitry by liquid-phase structuring of multi-material cores | SPIE Photonics West. In *Advanced Fabrication Technologies for Micro/Nano Optics and Photonics XVI, Vol. 12433*.12433071–124330713 (SPIE, 2023).

[30] Mowlavi S, Shukla I, Brun PT, Gallaire F (2019). Particle size selection in capillary instability of locally heated coaxial fiber. Phys. Rev. Fluids.

[31] Shukla I (2021). Reduced model for capillary breakup with thermal gradients: Predictions and computational validation. Phys. Fluids.

[32] van der Elst LA (2023). Microstructured Electroceutical Fiber-Device for Inhibition of Bacterial Proliferation in Wounds. Adv. Mater. Interfaces.

[33] Wang, F. *New Modeling of Compact, High-efficiency, and Widely-tunable Gas-phase Terahertz Lasers*. (MIT, 2019).

[34] Plateau, J. *Experimental and theoretical statics of liquids subject to molecular forces only*. *Gauthier-Villars*, *Paris* (1873).

[35] Utada AS (2005). Monodisperse Double Emulsions Generated from a Microcapillary Device. Science.

[36] Doremus RH (2002). Viscosity of silica. J. Appl. Phys..

[37] Danielson DT, Sparacin DK, Michel J, Kimerling LC (2006). Surface-energy-driven dewetting theory of silicon-on-insulator agglomeration. J. Appl. Phys..

[38] Coucheron DA (2016). Laser recrystallization and inscription of compositional microstructures in crystalline SiGe-core fibres. Nat. Commun..

[39] COMSOL, A. B. *COMSOL Multiphysics.* www.comsol.com

[40] Petropoulou A, Drikakis D, Riziotis C (2019). Microspheres Formation in a Glass–Metal Hybrid Fiber System: Application in Optical Microwires. Materials.

[41] Grodkiewicz WH (1975). Fused silica fibers with metal cores. Mater. Res. Bull..

[42] Homa D, Kaur G, Pickrell G, Scott B, Hill C (2014). Electronic and magnetic fibers. Mater. Lett..

[43] Zhang T (2017). High-performance, flexible, and ultralong crystalline thermoelectric fibers. Nano Energy.

[44] Homa D, Liang Y, Pickrell G (2013). Superconducting fiber. Appl. Phys. Lett..

[45] Ballato J (2010). Binary III-V semiconductor core optical fiber. Opt. Express.

[46] Ballato J, Snitzer E (1995). Fabrication of fibers with high rare-earth concentrations for Faraday isolator applications. Appl. Optics.

[47] Scott, B. L. & Pickrell, G. R. *Fabrication of GaSb Optical Fibers. in Processing and Properties of Advanced Ceramics and Composites V* vol. 240 65–70 (Wiley, 2013).

[48] Sangiorgi R, Muolo ML, Chatain D, Eustathopoulos N (1988). Wettability and Work of Adhesion of Nonreactive Liquid Metals on Silica. J. Am. Ceram. Soc..

[49] Nolte, D. D. Coupled Oscillators and Synchronization. *Introduction to Modern Dynamics* 177–204. 10.1093/OSO/9780198844624.003.0006 (2019).

[50] Patsyk A, Sivan U, Segev M, Bandres MA (2020). Observation of branched flow of light. Nature.

[51] Lopez-Herrera JM (1999). One-Dimensional Simulation of the Breakup of Capillary Jets of Conducting Liquids. Application to E.H.D. Spraying. JAERS.

[52] Luo H, Svendsen HF (1996). Theoretical model for drop and bubble breakup in turbulent dispersions. AIChE J..

[53] Faccini de Lima, C. et al. Multimaterial Fiber as a Physical Simulator of a Capillary Instability: Source Data and Code. *Zenodo* https://zenodo.org/record/8256497, 10.5281/ZENODO.8256497 (2023). 10.1038/s41467-023-41216-7 PMC10522671 37752148

