# Supplementary material for: Multimaterial fiber as a physical simulator of a capillary instability
Source: Nat Commun. 2023 Sep 26;14:5816. doi: 10.1038/s41467-023-41216-7 (PMC10522671; doi:10.1038/s41467-023-41216-7)
Supplement: Supplementary file 1 — Supplementary Information [file 41467_2023_41216_MOESM1_ESM.pdf]

Multimaterial Fiber as a Physical Simulator of a Capillary Instability

Supplementary Note 1. Model derivation – Geometrical Argument

This section describes the mathematical steps in the model derivation through a geometrical argument. As mentioned previously, we are interested in describing the capillary breakup behavior in a regime that is not contemplated by traditional models, as described in the main text: namely of viscous fluids in a non-isothermal regime, where the fluid thread is fed through a hot zone, usually a flame or laser, which induces a propagating temperature gradient. Once the fiber is inside the hot zone of the flame, the Si core liquefies, and the silica cladding softens. While the core plus cladding system continues to move through the hot zone in the axial direction x, surface tension minimization drives the pinching-off of the core into droplets at an approximately constant pinching location x_{bu} for each feed speed (Figure 2), as is observed experimentally. At this location, the core thread reshapes into a droplet and pinches off while a critical amount of material is fed into the droplet. This results in uniformly spaced spheres with a wavelength λ.

To understand the process shown above, we consider that the amount of core material that is fed into the droplet before it breaks off the core at the time $\tau = t_{pinch}$ (pinch time, defined in the main text) is equivalent to that stored in a section of a core of length λ being fed for a time t_g (growth time). This relationship is mathematically expressed as:

$$\tau = \frac{\lambda}{v_f} \qquad \text{(S1)}$$

, where τ may be a function of interface energy, viscosity and λ, such that $\tau = \tau(x, \lambda)$. Moreover, we state that the derivative of the neck thickness around the breakup point x_{bu} is smooth and equal to 0 at x_{bu}.

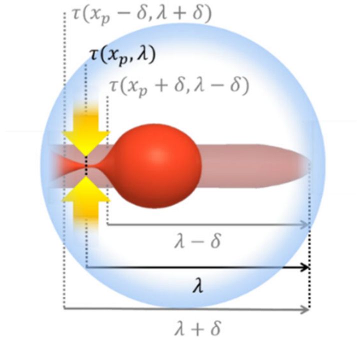

Supplementary Fig. 1. **Illustration of pinch-off process of the core thread at position x_{bu}.** The light red capped cylinder in the background represents the intact core thread shape, in comparison to the reshaped core in solid red shown on the foreground, representing the core during the pinch-off process at a time τ. The location of the pinch is highlighted by the yellow arrows.

In Supplementary Fig. 1, we show an illustration of the pinch-off process: Once the length λ is fed through the pinching front at x_{bu}, a breakup occurs, and at this time, the neck thickness is zero. We can also define the pinching speed v_p as the ratio between a' and the pinch-off time τ where a' is the difference between the thread radius a, and the neck half-thickness $d_{1/2}$ at the front location.

Let us now consider what happens in a position $x_{bu} + \delta$, where δ is an infinitesimally small quantity: at the same moment as the pinching is occurring at x_{bu}, it hasn't yet occurred at $x_{bu} + \delta$. Moreover, the material has begun to be fed through $x_{bu} + \delta$ later than at x_{bu} by a factor of $\tau' = {}^{\delta}/_{v_f}$. In this way, we can define an "excessive pinching time" τ^+ as the pinching time τ at $x_{bu} + \delta$ minus τ':

$$\tau^+ = \tau(\lambda - \delta, x_{bu} + \delta) - \frac{\lambda - \delta}{v_f} \quad \text{(S2)}$$

Similarly, for the pinching front $x_{bu} - \delta$, we have:

$$\tau^- = \tau(\lambda + \delta, x_{bu} - \delta) - \frac{\lambda + \delta}{v_f} \quad \text{(S3)}$$

By multiplying the pinch-off time by a', we can obtain the half-thickness $d^{\pm}_{1/2}$ of the neck at the locations $x_{bu} \pm \delta$, at the moment of pinch-off, which are expressed in Eq. (S2) and (S3), and illustrated in Supplementary Fig. 2:

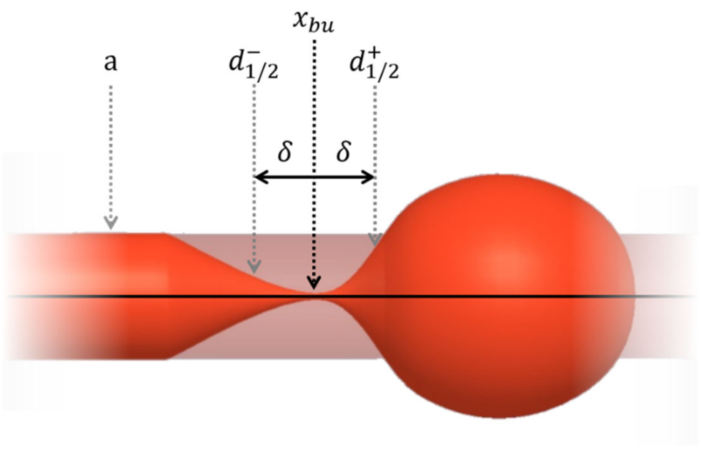

Supplementary Fig. 2. **Schematic representation of the fiber core during pinch-off.**

$$d_{1/2}^{+} = a\left(1 - \frac{\lambda+\delta}{v_f}\,\frac{1}{\tau(\lambda+\delta,\, x_{bu}-\delta)}\right) \tag{S4}$$

$$d_{1/2}^{-} = a\left(1 - \frac{\lambda-\delta}{v_f}\,\frac{1}{\tau(\lambda-\delta,\, x_{bu}+\delta)}\right) \tag{S5}$$

As stated before, we propose that the derivative of the neck thickness at x_{bu} is smooth and equal to 0:

$$\frac{\partial}{\partial\delta}\left[a\left(1 - \frac{\lambda-\delta}{v_f}\,\frac{1}{\tau(\lambda-\delta,\, x_{bu}+\delta)}\right)\right]_{\delta=0} = 0 \tag{S6}$$

$$\left[\tau(\lambda-\delta, x_{bu}+\delta) + (\lambda-\delta)\frac{d\tau}{d\delta}\right]_{\delta=0} = 0 \tag{S7}$$

Since $\tau = \tau(x,\lambda)$, we write the partial derivative on τ as

$$\frac{d\tau}{d\delta} = \frac{\partial\tau}{\partial x}\frac{\partial x}{\partial\delta} + \frac{\partial\tau}{\partial\lambda}\frac{\partial\lambda}{\partial\delta} = \left(\frac{\partial}{\partial x} - \frac{\partial}{\partial\lambda}\right)\tau \tag{S8}$$

So we can rewrite Eq. (S7) as:

$$\tau(\lambda, x_{bu}) + \lambda\left(\frac{\partial}{\partial x} - \frac{\partial}{\partial\lambda}\right)\tau = 0 \tag{S9}$$

Substituting τ from Eq. (S1), and dividing Eq. (S9) by λ, we obtain:

$$\frac{1}{v_f} + \left(\frac{\partial}{\partial x} - \frac{\partial}{\partial\lambda}\right)\tau(\lambda, x_{bu}) = 0 \tag{S10}$$

Thus, from Eq. (S1) and (S10), we obtain the following set of two equations in two variables from which we can obtain our solution:

$$\begin{cases} \left.\left(\dfrac{\lambda}{v_f} - \tau(\lambda, x)\right)\right|_{x_{bu}} = 0 \\ \left.\left(\dfrac{\partial}{\partial x} - \dfrac{\partial}{\partial\lambda}\right)\left(\dfrac{\lambda}{v_f} - \tau(\lambda, x)\right)\right|_{x_{bu}} = 0 \end{cases} \tag{S11}$$

Solving this system, however, requires information about the breakup time τ. Considering that $\eta_{clad} \gg \eta_{core}$, we have

$$\tau \sim \frac{\beta a\, \eta_{clad}}{\gamma} \tag{S12}$$

, where β is a system-dependent function of viscosity contrast between the core and the cladding materials. In some specific cases, for large viscosity contrast, β converges asymptotically to a constant derived by solving the Marginal Stability Criterion for propagating Plateau-Rayleigh instability case, as is done in Powers *et al.* (1998)[1], and to a different constant assuming the classical Tomotika's growth rate.

Feeding Eq. (S12) into (S11), and considering that, for silica, $\mu_{clad} = \mu_{clad}\big(T(x)\big) = Ae^{B/T(x)}$, where $A = 5.7 \times 10^{-8}\, Pa\ \cdot s^{-1}$ and $B = 61812\, K$ are material properties[2], we rewrite (S11) as:

$$\begin{cases} \left.\left(\dfrac{\lambda}{v_f} - \dfrac{\beta a A e^{B/T(x)}}{\gamma}\right)\right|_{x_{bu}} = 0 \\ \left.\left(\dfrac{\partial}{\partial x} - \dfrac{\partial}{\partial \lambda}\right)\left(\dfrac{\lambda}{v_f} - \dfrac{\beta a A e^{B/T(x)}}{\gamma}\right)\right|_{x_{bu}} = 0 \end{cases} \tag{S13}$$

Supplementary Note 2. Lagrangian derivation

Starting with

$$\frac{d}{dt}\left(\frac{\partial L}{\partial \dot{q}}\right) - \frac{\partial L}{\partial q} = 0 \tag{S14}$$

It holds that

$$\frac{d}{dt}\left(\frac{\partial C F^{v_f}(\lambda, x)}{\partial \dot{q}}\right) - \frac{\partial C F^{v_f}(\lambda, x)}{\partial q} = 0 \tag{S15}$$

with $\dot{q} = v_f$ and $q = \lambda$. We can expand (S15) as

$$\frac{d}{dt}\left(C\frac{\partial F^{v_f}(\lambda, x)}{\partial v_f} + F^{v_f}\frac{\partial C}{\partial v_f}\right) - \left(C\frac{\partial F^{v_f}(\lambda, x)}{\partial \lambda} + F^{v_f}\frac{\partial C}{\partial \lambda}\right) = 0 \tag{S16}$$

As both $\frac{dC}{dt} = 0$ and $\frac{dF^{v_f}}{dt} = 0$, (16) becomes

$$\left[C\frac{d}{dt}\left(\frac{\partial F^{v_f}(\lambda, x)}{\partial v_f}\right) + F\frac{d}{dt}\left(\frac{\partial C}{\partial v_f}\right)\right] - \left[\left(C\frac{\partial F^{v_f}(\lambda, x)}{\partial \lambda}\right) + \left(F^{v_f}\frac{\partial C}{\partial \lambda}\right)\right] = 0 \tag{S17}$$

We can easily see that since $\frac{\partial C}{\partial v_f} = 2\pi a\, \gamma$, it follows that $\frac{d}{dt}\left(\frac{\partial C}{\partial v_f}\right) = 0$. Also, $\frac{\partial C}{\partial \lambda} = 0$, and thus we have:

$$C\frac{d}{dt}\left(\frac{\partial F^{v_f}(\lambda, x)}{\partial v_f}\right) - C\frac{\partial F^{v_f}(\lambda, x)}{\partial \lambda} = 0 \tag{S18}$$

We can verify that (S18) is true by performing the derivatives on $F^{v_f}(\lambda, x)$:

$$\frac{d}{dt}\left(\frac{\partial F^{v_f}(\lambda, x)}{\partial v_f}\right) = \frac{d}{dt}\left[\frac{\partial}{\partial v_f}\left(\frac{\lambda}{v_f} - \frac{\beta a\, \eta_{clad}}{\gamma}\right)\right] \tag{S19}$$

$$\frac{d}{dt}\left(\frac{\partial F^{v_f}(\lambda, x)}{\partial v_f}\right) = \frac{d}{dt}\left(-\frac{\lambda}{v_f^2}\right) = -\frac{1}{v_f^2}\frac{dx}{dt}\frac{d\lambda}{dx} = \frac{1}{v_f} \tag{S20}$$

And

$$\frac{\partial F^{v_f}(\lambda, x)}{\partial \lambda} = \frac{\partial}{\partial \lambda}\left(\frac{\lambda}{v_f} - \frac{\beta a\, \eta_{clad}}{\gamma}\right) = \frac{1}{v_f} \tag{S21}$$

Therefore,

$$\frac{d}{dt}\left(\frac{\partial F^{v_f}(\lambda, x)}{\partial v_f}\right) = \frac{\partial F^{v_f}(\lambda, x)}{\partial \lambda} \tag{S22}$$

Supplementary Note 3. Silicon-Silica Surface Tension

The value of the surface tension γ_{Si-SiO_2} between liquid silicon and silica was obtained as follows:

In Fujii *et al.* (2006)[3] , the surface tension γ_{Si} of molten silicon is measured by the microgravity oscillating drop method and the following relation between γ_{Si} (in mN/m) and the temperature T, is presented:

$$\gamma_{Si} = 733 - 0.062(T - 1687) \qquad \text{(S23)}$$

for a temperature range of 733 K to 1890K, in agreement with Lucas *et al.* (1984)[4].

From Parikh (1985)[5], we get that the surface tension of silica is $\gamma_{SiO_2} = 280\ mJ/m^2$. In Li *et al.* (1992)[6], the wettability of silica by molten silicon is investigated through the sessile drop method, and the contact angle θ of silicon on silica was determined to be 92° at 1703 K. The work of adhesion W is then given by:

$$W = \sigma_{SL}(1 - cos\theta) = 708\ mJ/m^2 \qquad \text{(S24)}$$

With $\sigma_{SL} = \gamma_{Si} + \gamma_{SiO2} - \gamma_{Si-SiO_2}$.

Thus, we can calculate γ_{Si-SiO_2} as

$$\gamma_{Si-SiO_2} = \gamma_{Si} + \gamma_{SiO_2} - W = 0.298\ J/m^2 \sim 0.3\ J/m^2 \qquad \text{(S25)}$$

which is consistent with the values found in the literature[7,8].

Supplementary Note 4. Capturing the breakup:

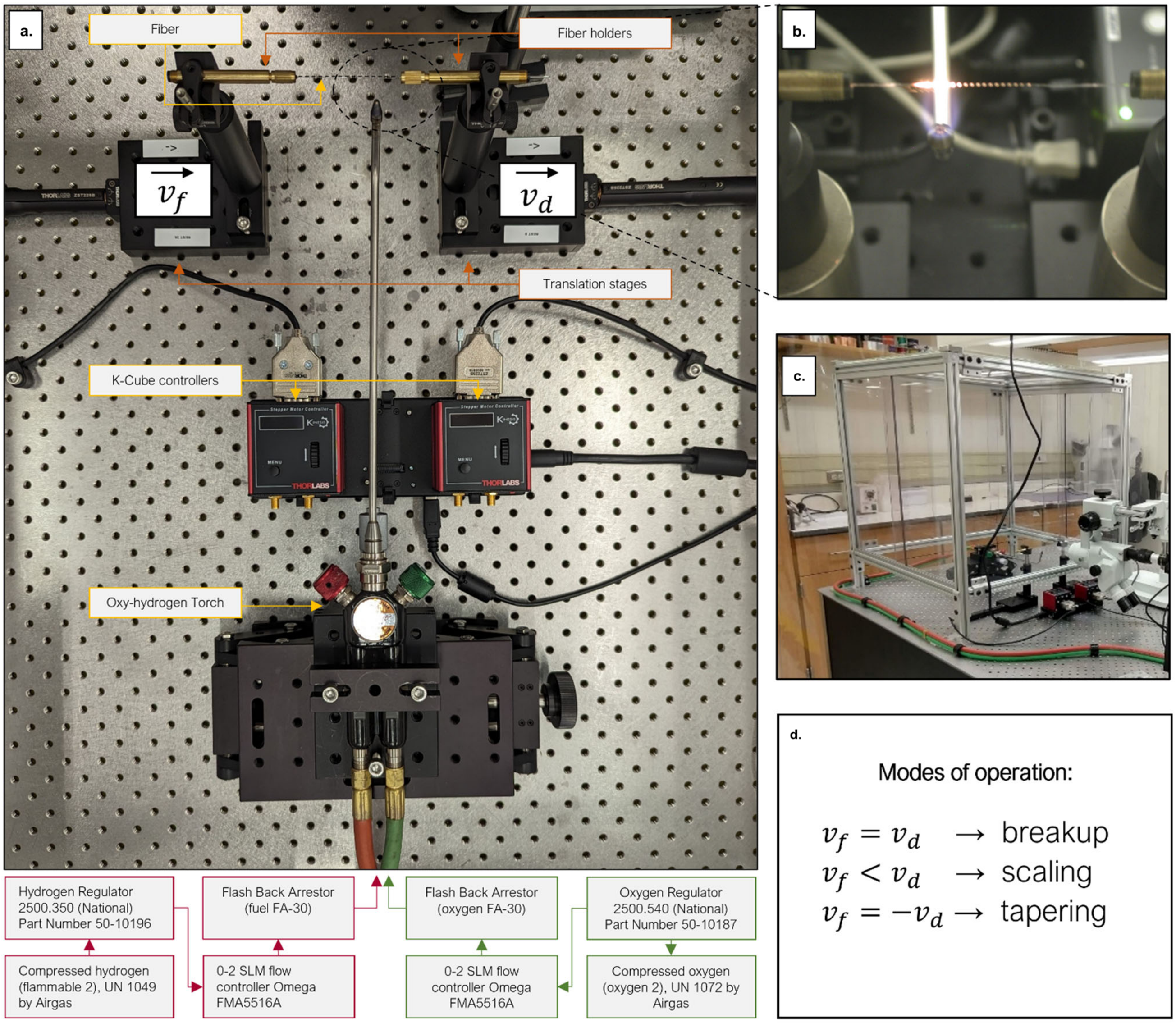

Supplementary Fig. 3. **High-Temperature Breakup Setup. a** top-view of the setup, with main elements identified. The gas components are indicated in the red (hydrogen) and green (oxygen) boxes on the bottom of the picture., and are connected using a Twin Welding Hose (Nationaltorch CT-12-3AB). The torch (Nationaltorch 3H Hydrogen/Oxygen Torch 34-10099) is mounted on a Lab jack (Thorlabs L200) and a 1" Translation Stage with Standard Micrometer, 1/4"-20 Taps PT (Thorlabs PT1), and has a 0.02” stainless steel tip attached (Nationaltorch MSOX-0 Part Number 34-10314-2). The fiber is secured on fiber holders (Newport), which are mounted on V-clamps (Thorlabs VC1) attached to pillar posts and holders (Thorlabs ½” optical hodlers series). Each post is then secured to 1” translation stages (Thorlabs PT1) equipped with a stepper motor (Thorlabs ZST225B), controlled individually by a set of K-Cube controllers (Thorlabs KST101), such that the translation speeds can be set independently. **b** Inset of the fiber and torch tip during a breakup process, where the core of the fiber can be observed as continuous before it is fed through the torch, and broken up into spheres, afterwards. **c** Picture of the setup installed on top of an optical table. A plexiglass cage was installed around the setup to minimize air flow disturbances on the torch and ensure its stability. A boom microscope (Amscope ZM-4TW3-FOD-9M) equipped with a digital camera (Amscope MU900/Motic Moticam 1000) is used to capture the breakup experiments. **d** Modes of operation: The setup can be used for capillary breakup experiments, when the feed speed v_f is equal to the draw speed v_d. When $v_f < v_d$, the system operates as a scaling setup, and when the stages are moving in opposite directions (for example $v_f = -v_d$), as a tapering setup.

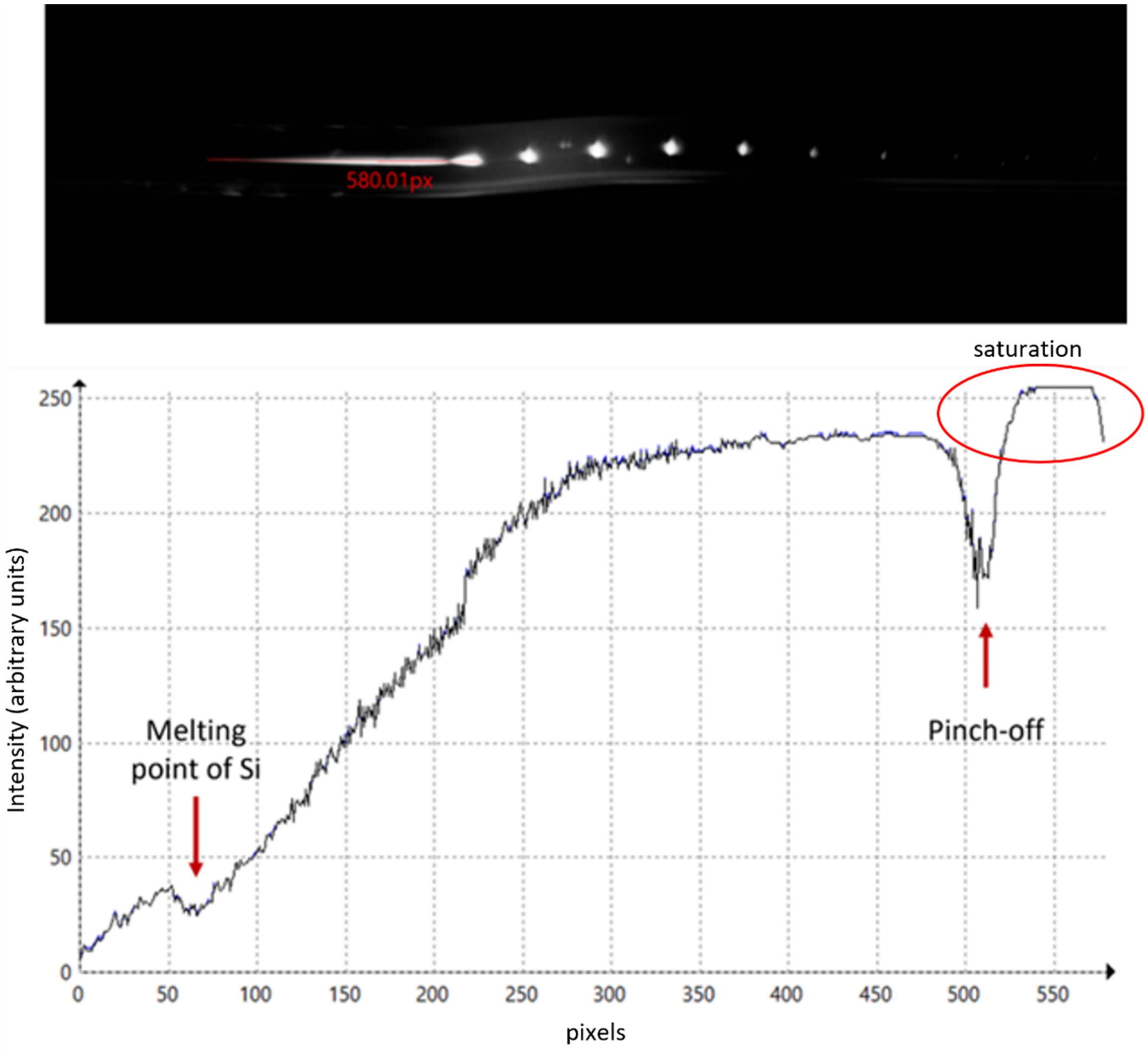

Supplementary Fig. 4. **Snapshot of a breakup experiment**. It illustrates how the breakup location is measured with respect to the melting position of silicon, which can be determined as a dip in the intensity profile (y-scale on the bottom graph is intensity in arbitrary units, x-scale is the number of pixels starting from right along the red line 580 pixels long on the image, along which the intensity profile was collected).

To capture the dip in emissivity associated with the Si melting point, the dynamic range is compromised: it can be noted that the signal saturates around pixel 550, thus emissivity corresponding for T_{max} is not captured. At the same time, in our experience, if the sensitivity of the camera is reduced, such that there is no saturation of the signal in the frame, the dip in the emissivity corresponding to the melting of silicon blends into the noise and can't be captured reliably. Hence the challenge of reconstructing the temperature profile from the emissivity signal, mentioned in the main text.

Supplementary Note 5. Model fitting to Experimental data

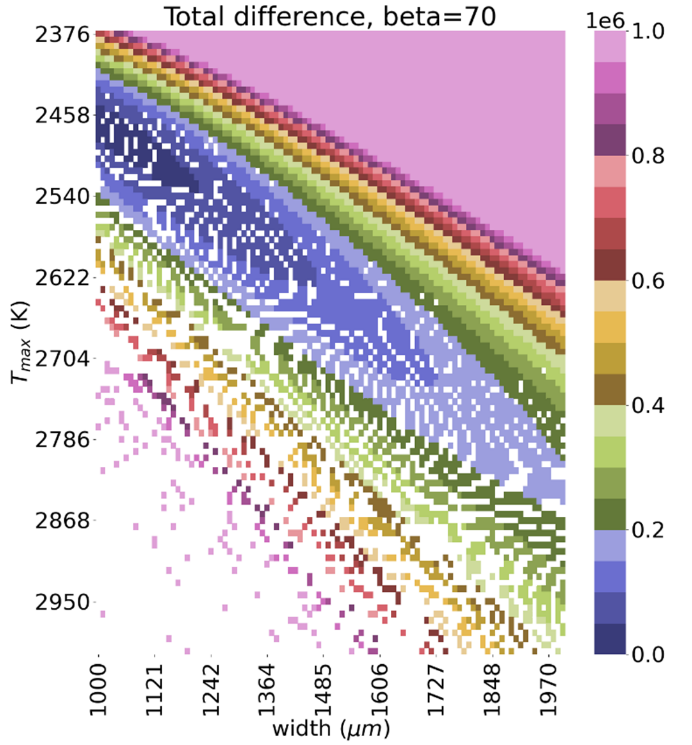

Supplementary Fig. 5. **Example map of the sum of the sum of the square difference for $\lambda|_{x_{bu}}$ and x_{bu} with respect to the experimental values, created by sweeping the temperature parameters T_{max} and w.**

To determine the best temperature profile parameters to fit the model to the experimental results, a simple algorithm was developed: for any given combination of temperature profile parameters, the sum of the square difference (ssd) between the model prediction and the experimental results can be easily calculated. By sweeping the parameters T_{max} and w, a map is generated, which allows the identification of the parameters that yield the best model fit to the data. This was performed for each value of β evaluated.

Supplementary Note 6. COMSOL evaluation of the numerical constant β:

Through similar COMSOL[9] simulations, as performed in previous work by Faccini de Lima *et al.* (2019)[10], we evaluated β by comparing the pinch-off time of the first sphere off the initially continuous room-temperature core when exposed at time=0 to a static localized heat source set to generate various temperatures T (Supplementary Fig. 6). We considered a 4 μm-thick silicon core surrounded by a silica cladding, exposed to a temperature profile described in Ref.[10]. The surface tension between silica and silicon was set to $\gamma = 0.3$ N/m. The results, shown in Supplementary Fig. 6, indicate good agreement between the COMSOL simulation results and our model predictions for $\beta = 60 \pm 10$, with respect to the time needed for the first pinch-off in a stand-still initially continuous fiber core to occur while at time zero exposed to the hydrogen-oxygen flame.

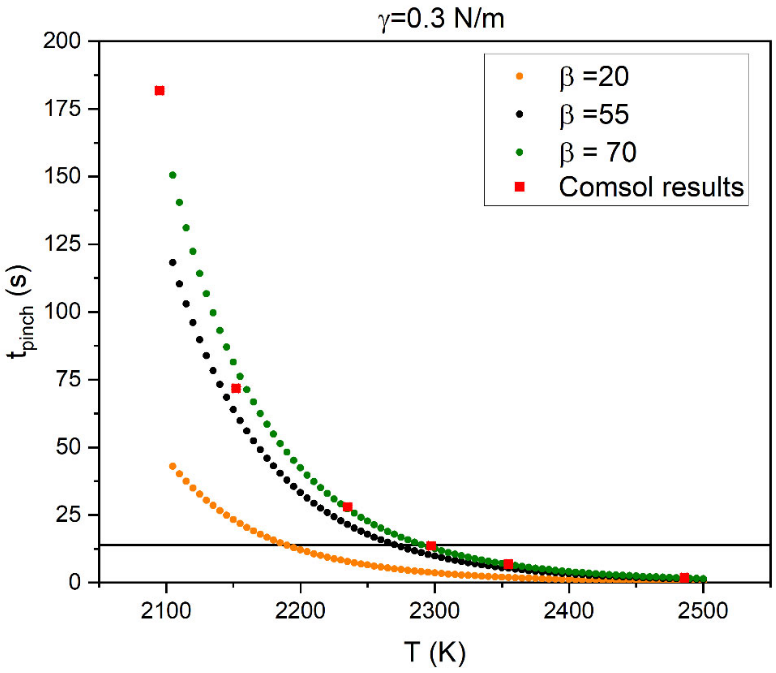

Supplementary Fig. 6. **Pinch-off time t_{pinch} as function of temperature T for a 4 μm-thick silicon core surrounded by silica, under surface tension of 0.3 N/m.** The results obtained from COMSOL simulations are compared to the AVG Instability model predictions under assumption of different β values, sowing best agreement for $\beta = 60 \pm 10$.

Supplementary Note 7. Capillary breakup simulation dependence on the computational resource:

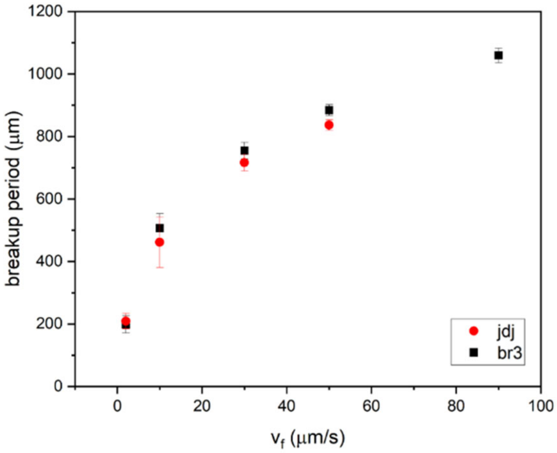

Supplementary Fig. 7. **Verification of the installation of the ab-initio simulation code on Indiana University's BR3 supercomputer and comparison with a simulation result obtained, for the same simulation parameters, at MIT's jdj cluster.** Simulations were run for a temperature profile of the form $T = T_{max} - (T_{max} - T_{Si})e^{-x/w}$, with $T_{max} = 2410\ K$ and $x = 2735\ \mu m$. Error bars – 1 σ.

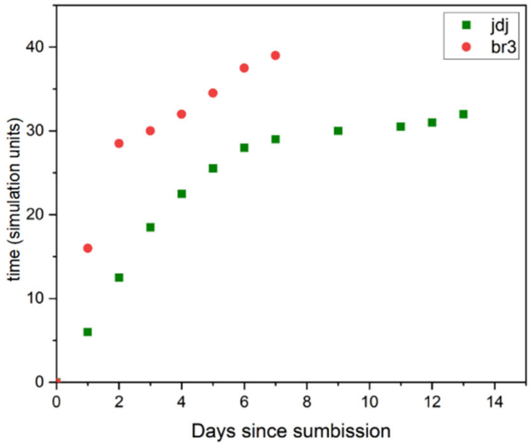

Supplementary Fig. 8. **Short-term acceleration.** Demonstrated is the ime propagation comparison between BR3 and jdj clusters under the simulation parameters described in S6, as a function of simulation run time (days since submission). The data shown represents the simulation progress for $v_f = 30\ \mu m/s$ from the start (time = 0) up to around the second breakup event (time ≈ 30 simulation units). One unit of time in the simulation is equivalent to 2.06 s. In this example we can see how the simulations ran at BR3 achieved longer time propagations, i.e., more time is simulated) within shorter run times.

At jdj the simulations were set to run on two cores of a single CPU. At BR3 the simulations were set to run on 32 CPU's, two cores on each. The short-term acceleration is depicted in Figure S8. The long-term acceleration is such that for the breakup process to reach the steady state (~3 breakup periods are simulated), it would take 4-6 months on jdj, compared to 2-3 weeks on BR3 depending on the feed speed simulated – an order of magnitude faster, although not fast enough to enable optimization calculations, only in combination with AVG-IM model, as is described in Figure 4 in the main text.

Supplementary Note 8. Si-core Fiber preform fabrication and draw results:

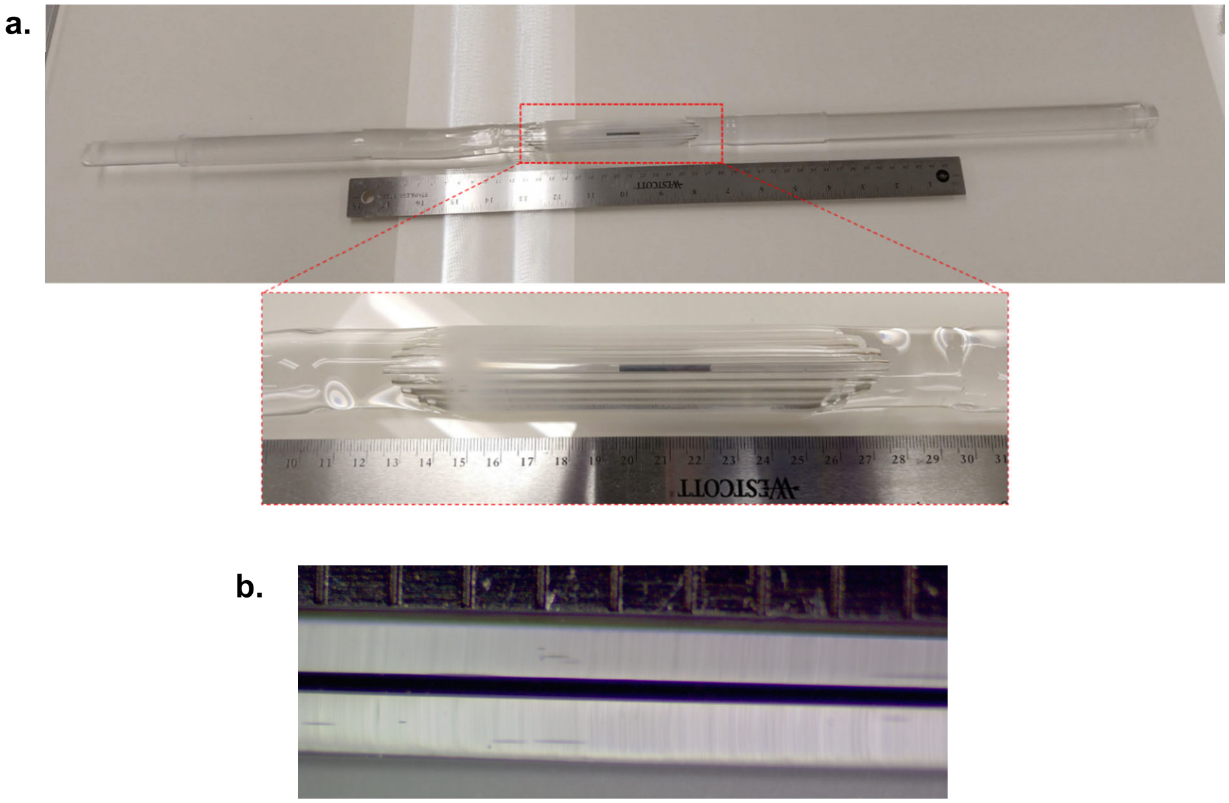

Supplementary Fig. 9: **Assembled preform for the fabrication of a silicon-core silica fiber. a** A silicon rod is inserted in concentric silica tubes and sealed under vacuum with a glass lathe. **b** The resulting first-draw cane is demonstrated next to a ruler with 1 mm ticks. This cane was stacked into a new preform, as is described in the Methods section and redrawn into a fiber on Supplementary Fig. 10.

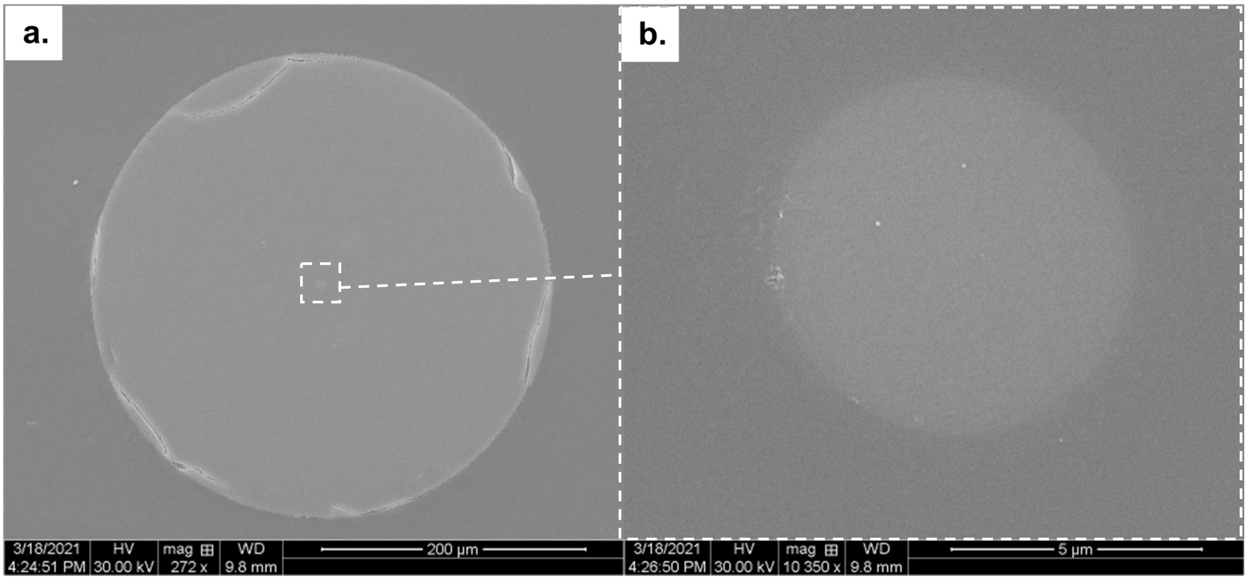

Supplementary Fig. 10. **Silicon-core fiber cross section. a** SEM image of the silicon core fiber cross-section. **b** Details of the silicon core.

Supplementary Note 9. Determining the framework of parameters relevant to the AVG-IM:

AVG-IM-relevant parameters can be roughly divided into two groups: the first relates to the fiber, and the second relates to the liquefaction zone it is fed through. The fiber-related properties are core and cladding dimensions, viscosities, and the interface energy between the two (five parameters). The relevant liquefaction zone properties are its maximal temperature and the width of its boundary (two parameters).

The fiber-related properties are core and cladding dimensions, viscosities, and the interface energy between the two (five parameters). The relevant liquefaction zone properties are its maximal temperature and the width of its boundary (two parameters). A quantitatively conclusive examination of the general applicability of AVG-IM would necessitate an exhaustive numerical and experimental exploration of capillary instability in at least the seven-dimensional space spanned by the parameters above. While such a fundamental study is of great interest to us, it would require resources significantly surpassing those currently available.

Instead, in this section, we want to outline the roadmap for assessing the AVG-IM applicability to solving technology-relevant problems by presenting several specific use-case studies where the seven parameters above vary in the subspace relevant to the engineering of optoelectronic fiber devices and systems. To be technologically relevant, the variation of each parameter needs to stem from an experimentally realistic scenario.

Let us start with the two experimentally justifiable simplifications that AVG-IM makes:

Model Simplification MS1: The cladding-to-air interface of the fiber is not significantly prone to capillary instability; thus, the cladding can be considered an infinitely large diameter. This simplification is reasonable since the fiber cladding diameter is much wider than the diameters of the in-fiber cores considered in this study. Thus, t_{pinch}, which is proportional to the liquid thread diameter under consideration, is significantly longer for the cladding than for the cores. Additionally, since the liquefaction zone is localized, and the feed speeds are finite, no section of the fiber dwells in the liquid state for long enough for the instability to develop significantly on the cladding-air interface. In other words, we assume that t_{pinch} of the cladding is much larger than $v_f \cdot W$, where W is the overall width of the liquefaction zone.

This simplification needs to be reconsidered case-by-case in scenarios where the cladding diameter is not axially uniform, such as tapered fibers, the breakup recently becoming a topic of great technological interest [11]. The experimental verification of its validity for the cladding dimensions of the etalon fiber from Figure 6 is demonstrated in Supplementary Fig. 11.

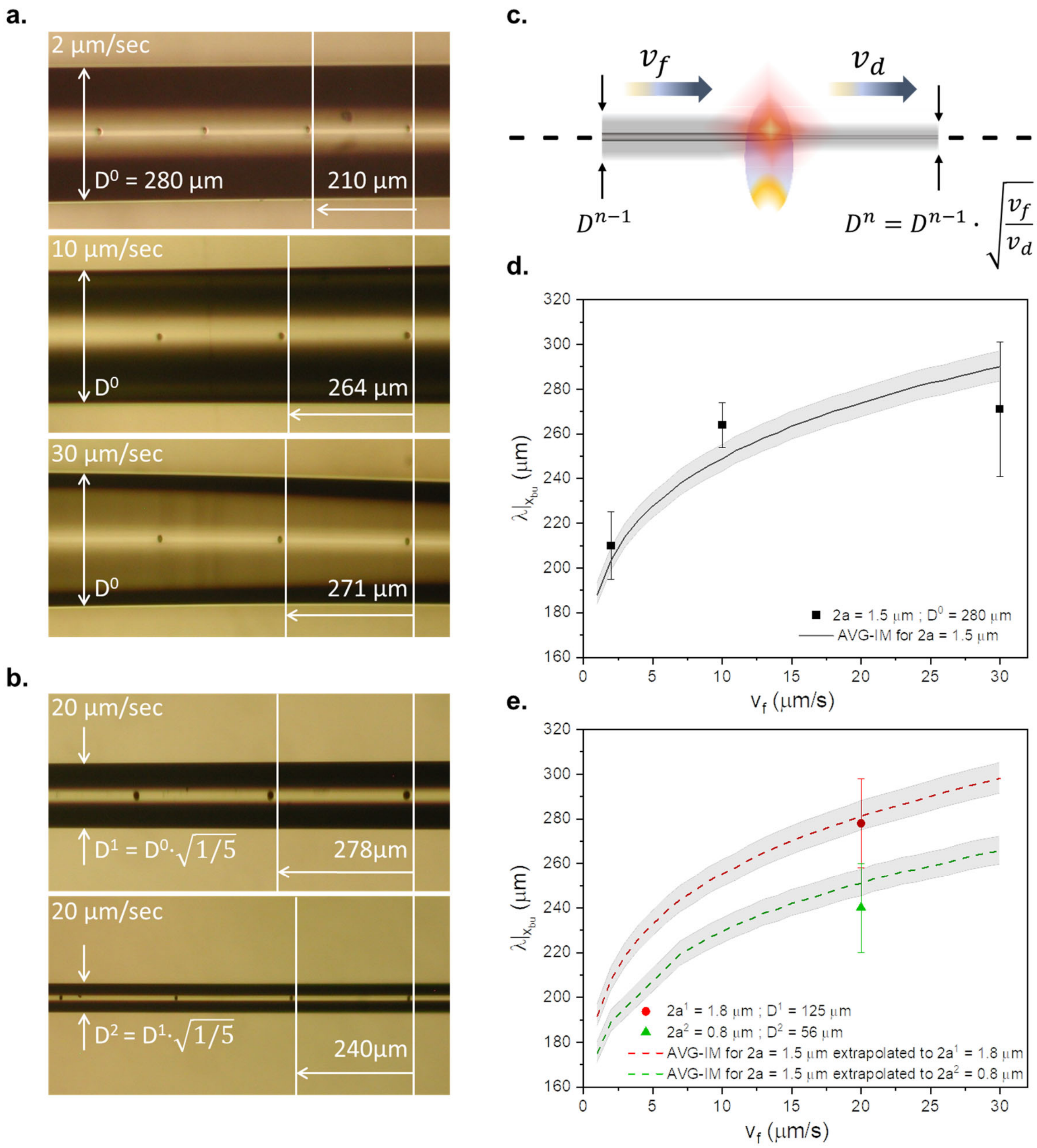

Supplementary Fig. 11. **Extrapolation of AVG-IM to tapered fiber cores and claddings. a, b** Breakup of Si-core fibers in the flame resulting from H_2 flow of 0.3 l/min and O_2 flow of 0.2 l/min: **a** - Optical micrographs of the breakup in 280 µm thick silica fiber with 1.5 µm silicon core at varying feed speed; **b** - Optical micrographs of the breakup in 280 µm thick silica fiber with 4.0 µm silicon core scaled down in thickness by a factor of $\sqrt{5}$ once (top, cladding 125 µm, core 1.8 µm) and twice (bottom, cladding 56 µm, core 0.8 µm). **c.** Schematics of the scale-down procedure (for details, see Methods). **d, e** AVG-IM applicability examination: **d** - AVG-IM fit to the 1.5 µm core breakup results yielding the temperature profile $T(x) = T_{max} - (T_{max} - T_{Si})e^{-x/w}$ with $T_{max} = 3050\ ^0K$ and $w = 2975\ \mu m$; **e** - Extrapolation of AVG-IM to 1.8 µm and 0.8 core breakup compared to the experimental results. Notation: $D^i, a^i : i = \{0,1,2\}$ are cladding diameter and core radius past the scaling step i. Error bars – 1 σ.

Here, we investigated the breakup in the fiber in Supplementary Fig. 11a, which has the same cladding diameter $D^0 = 280\ \mu m$ as that of the etalon fiber in Figure 6, yet a significantly smaller core diameter $2a =$

1.5 μm. Using AVG-IM interpolation (Supplementary Fig. 11d), we have reconstructed the temperature profile of the hot zone created by the flame. Using the same flame settings, we have performed a breakup of the fibers scaled down, as is shown in Supplementary Fig. 11c, from the etalon fiber such that the cladding dimensions of the scaled-down sections $D^1 = 125\ \mu m$ and $D^2 = 56\ \mu m$ are significantly smaller than D^0, yet the core dimensions $2a^1 = 1.8\ \mu m$ and $2a^2 = 0.8\ \mu m$ are comparable to $2a$ (Supplementary Fig. 11b).

The fiber in Supplementary Fig. 11b resulted from scaling the etalon fiber (the one used for the experiments in Figure 6, with a 280 μm-thick silica cladding and a 4 μm-thick Si core) in two steps according to the procedure schematically depicted in Supplementary Fig. 11c.

Next, we verified that AVG-IM extrapolates well to the results of the breakup in fibers with thinned-out claddings (Supplementary Fig. 11e), thus supporting experimentally that AVG-IM is independent of the cladding dimensions, i.e., MS1 holds.

Even if MS1 holds, the cores of the tapered fibers are tapered too, i.e., $a = a(x)$, thus AVG-IM needs to be verified for varying core dimensions. Figures 6 and Supplementary Fig. 11 present an experimental verification that AVG-IM describes well the breakup for four discrete core diameters – 4, 1.8, 1.5, and 0.8 µm, thus we know that AVG-IM will hold for adiabatically tapered cores in this range of core dimensions. In the general tapered-fiber case, $a = a(x)$ must be considered to be changing continuously and significantly within a single breakup period. We hypothesize that the central equation of AVG-IM, Eq. (2), will hold for such tapered cores if Ca in it is replaced with a modified Ca^{taper} accordingly, i.e., $\mathrm{Ca}^{taper}(\lambda, x) \equiv \frac{\beta a(x)\, \eta_{clad}(x)\, v_f}{\gamma\lambda}$, although the validity of the model and its limitations, in that case, need to be reexamined experimentally and numerically as part of the future follow-up study.

Model Simplification MS2: The fiber cores are solid crystalline materials that abruptly change the aggregation state upon melting, becoming an inviscid liquid. This simplification results in the core-cladding viscosity contrast $\eta_{core}/\eta_{clad} \to 0$ in the temperature range relevant for the development of capillary instability and allows us to consider only the cladding viscosity $\eta_{clad}(x)$ past the melting point of the core $x = 0$ to define the AVG-IM. It holds true for a wide variety of core materials relevant for silica-fiber embedded optoelectronics and photonics, including but not limited to semiconducting, metallic, magnetic, optically non-linear, superconducting, thermoelectric, ferroelectric, and piezoelectric [2,12–20].

The situation is different for the polymer-fiber embedded devices and systems, where amorphous materials, such as chalcogenides[21,22] or polymers different from this comprising the cladding[23], are used for the cores functionalizing the fiber optically or electronically. For such fibers η_{core}/η_{clad} is finite. Thus, we

hypothesize that for the AVG-IM to hold true, Ca in Eq. (2) needs to be replaced with a modified $\mathrm{Ca}^{\frac{\eta_{core}}{\eta_{clad}}}$. The educated guess that we suggest for $\mathrm{Ca}^{\frac{\eta_{core}}{\eta_{clad}}}$ for scenarios considering a finite $\eta_{\mathrm{core}}/\eta_{\mathrm{clad}}$ is $\mathrm{Ca}^{\frac{\eta_{core}}{\eta_{clad}}}(\lambda, x) \equiv \beta \cdot v_f/[2\lambda \cdot in(\lambda, \eta_{clad}(x), \eta_{core}(x))]$, where $\mathrm{in}(\lambda, \eta_{clad}, \eta_{core})$ is the Tomotika instability rate. This choice is guided by the fact that $\mathrm{Ca}^{\frac{\eta_{core}}{\eta_{clad}}}(\lambda, x)$ converges towards $\mathrm{Ca}(\lambda, x)$ for $\eta_{core}/\eta_{clad} \rightarrow 0$, as is demonstrated in Methods. Although, once again, the validity of the model and its limitations for such extrapolation need to be reexamined experimentally and numerically.

Supplementary Note 10. Si vs. Cu breakup in a pure H_2 flame:

Presented in this section is the investigation of Si- and Cu-core fibers in pure hydrogen flame for the ability to yield same-wavelength breakup in desperate-material cores, scanning the feed speed, core radius, and maximal temperature of the liquefaction zone. Figures 8A and 8B show the subsets of the data set in Supplementary Note 10 that were collected in identical temperature profile conditions, and thus can be analyzed by AVG-IM, resulting from H_2 flow rate of 1.2 l/min and 0.4 l/min, respectively.

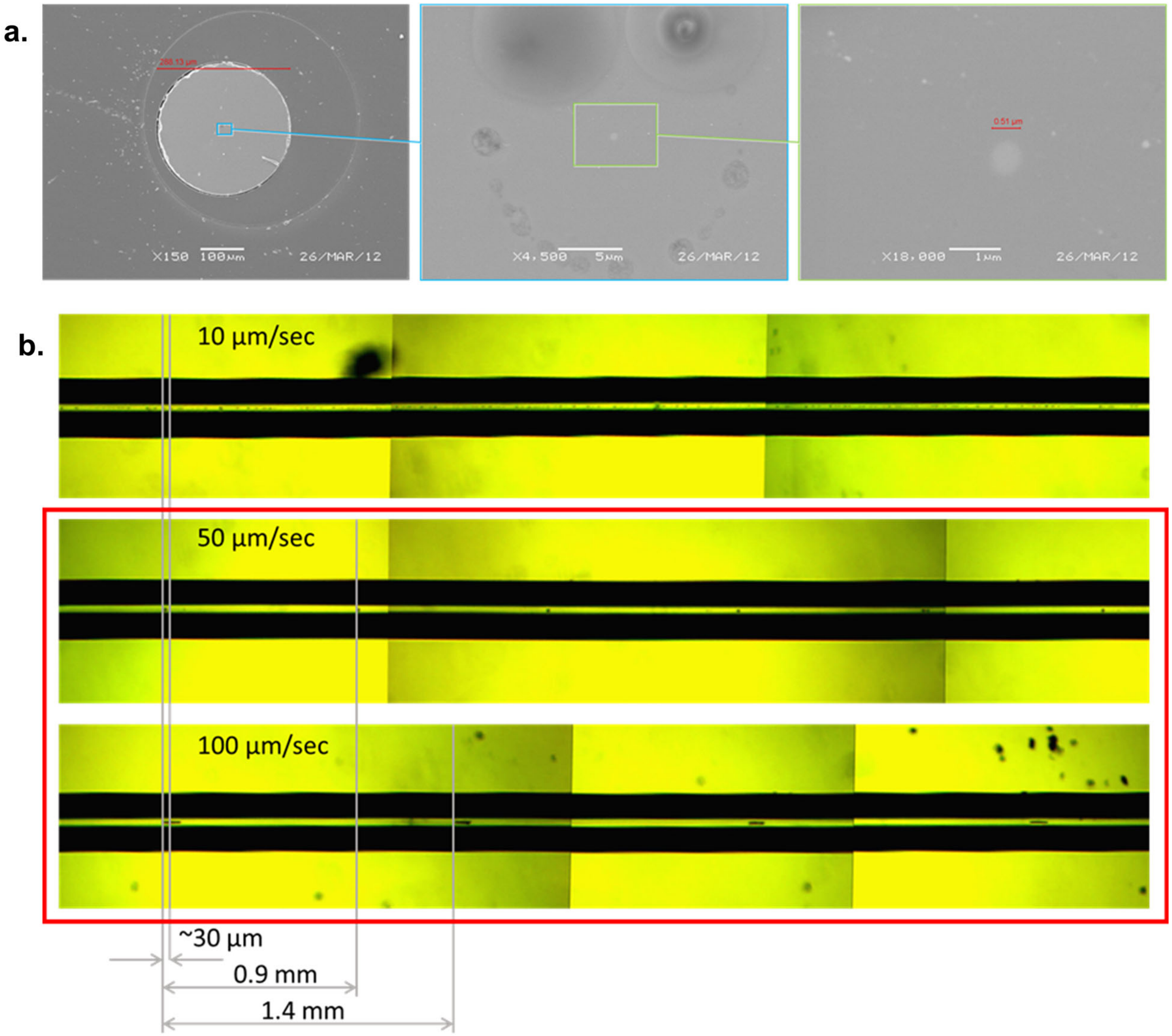

Supplementary Fig. 12. **Silicon-core breakup in a pure H_2 flame of constant gas flow at varying feed speed. a F**iber cross-section SEM: silica cladding of 288 µm, Si sore of 0.5 µm core; **b** Breakup results optical micrographs, side-view of the fiber: breakup conditions - H_2 flow of 1.2 l/min, varying feed speed. Red rectangle frames the data used for Figure 8 A in the main text. It is obvious that 0.5 µm core Silicon breakup at 10 µm/sec is chaotic (non-predictable), out of the Validity Limitations of AVG-IM. Elongated droplets result from the flame being too narrow for the full reshaping into a sphere to take place past the pinch-off before the droplet leaves the flame.

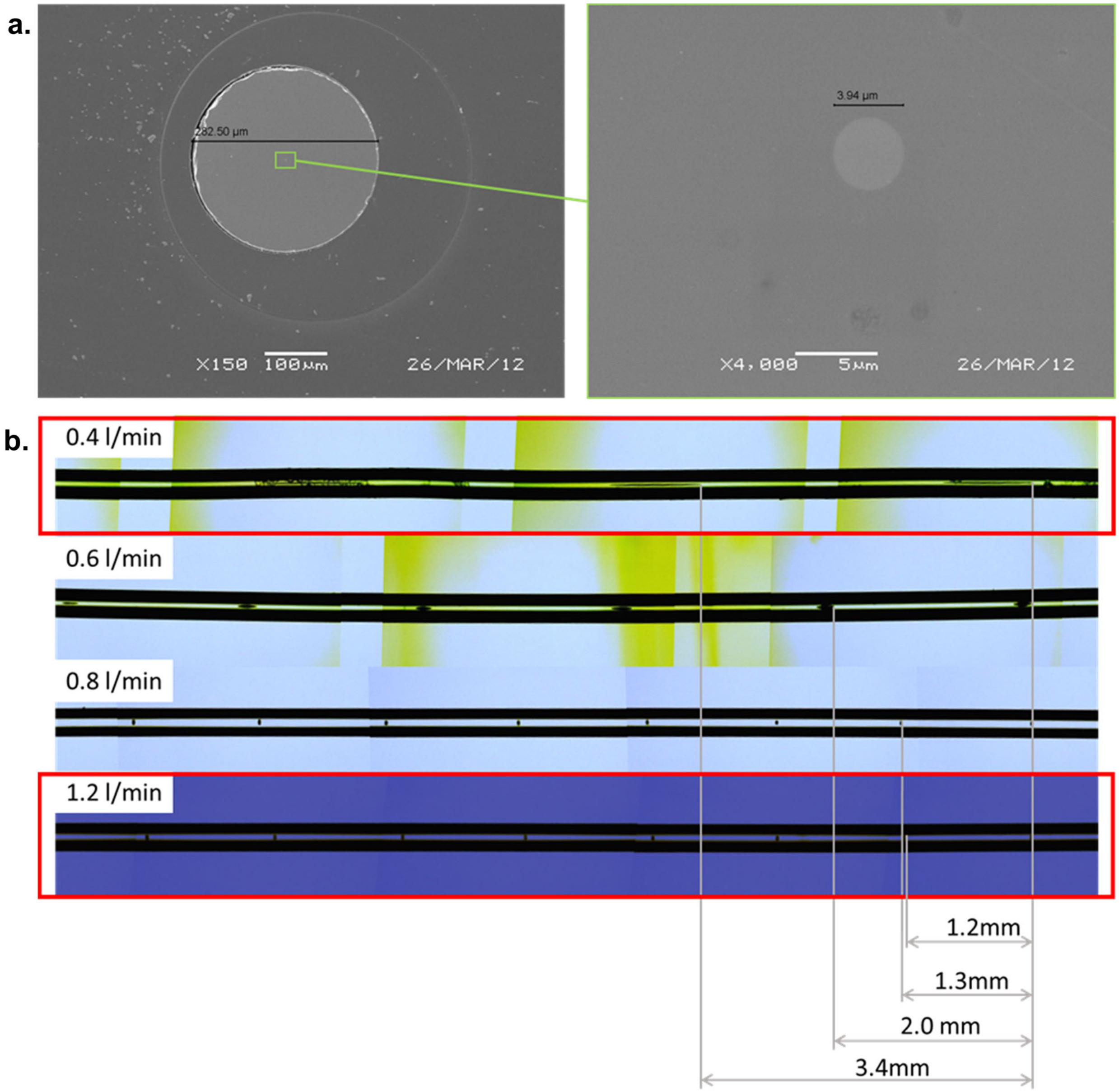

Supplementary Fig. 13. **Silicon-core breakup in a pure H_2 flame of varying gas flow at constant feed speed. a.** Fiber cross-section SEM: silica cladding of 282 µm, Si sore of 4 µm core; **b.** Breakup results optical micrographs, side-view of the fiber: breakup conditions - feed speed 10 µm/sec, varying H_2 flow. Red rectangles frame the data used for Figure 8 a,b in the main text. Elongated droplets result from the flame being too narrow for the full reshaping into a sphere to take place past the pinch off before the droplet leaves the flame.

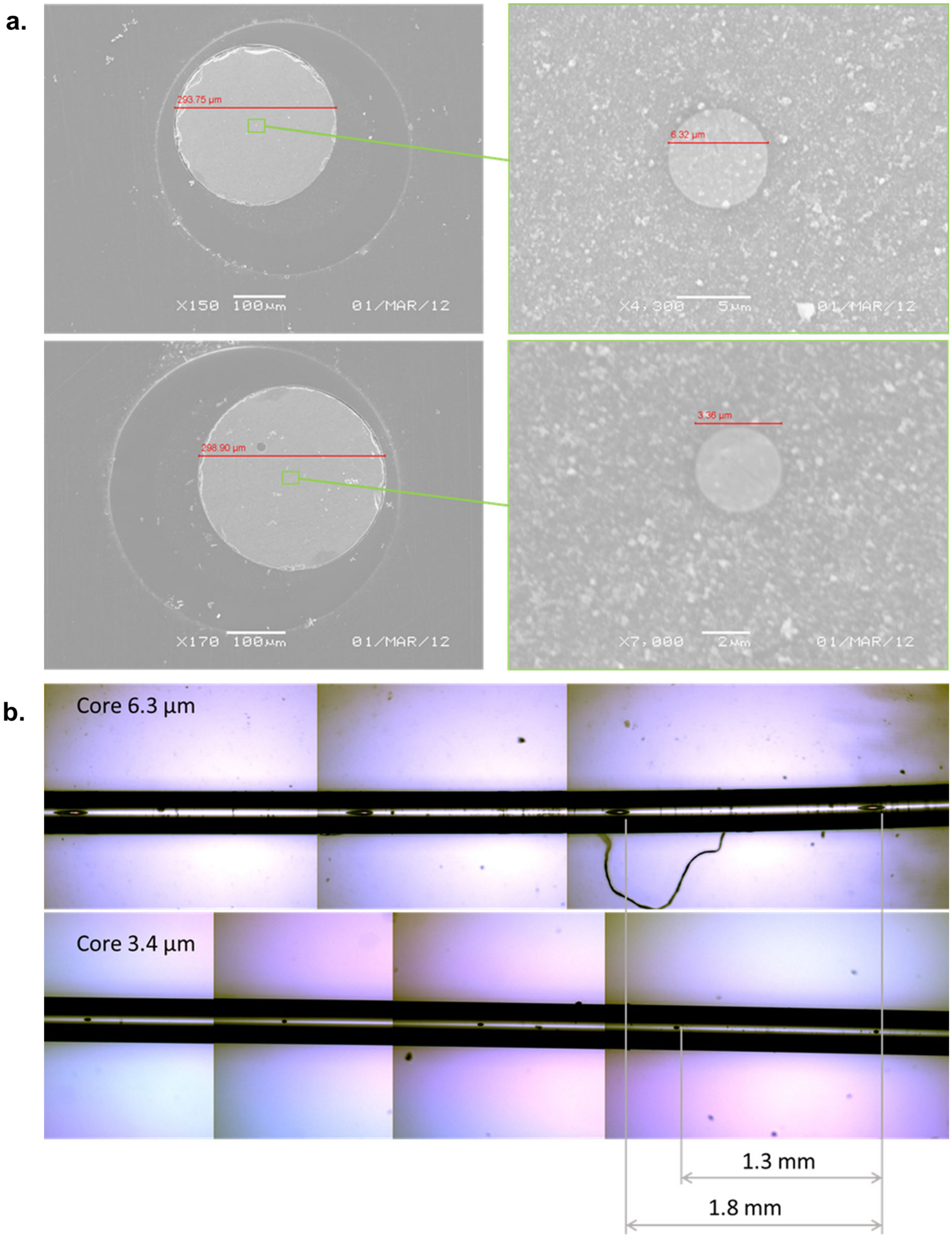

Supplementary Fig. 14. **Copper-core breakup in a pure H2 flame.** a Fiber cross-section SEMs: silica claddings of 294 and 299 µm with Cu cores of 6.3 and 3.4 µm cores, respectively. **b** Breakup results optical micrographs, side-view of the fiber: breakup conditions - feed speed 10 µm/sec, H_2 flow of 0.4 l/min. The data is used for Figure 8 b in the main text. Elongated droplets result from the flame being too narrow for the full reshaping into a sphere to take place past the pinch before the droplet leaves the flame.

From the results in Figures S12-S14 we notice that there are at least three sets of conditions yielding very similar breakup wavelengths:

$\lambda|_{x_{bu}} = 1380 \pm 20\mu m$: Si-core, H_2 flow rate = 1.2 l/min, $2a = 0.5\ \mu m$, and $v_f = 100\ \mu m/sec$

$\lambda|_{x_{bu}} = 1220 \pm 20\ \mu m$: Si-core, H_2 flow rate = 1.2 l/min, $2a = 4\ \mu m$, and $v_f = 10\ \mu m/sec$

$\lambda|_{x_{bu}} = 1310 \pm 20\ \mu m$: Cu-core, H_2 flow rate = 0.4 l/min, $2a = 3.4\ \mu m$, and $v_f = 10\ \mu m/sec$

Only the last two sets of conditions give a good starting point for looking for a staggered diode assembly scenario because they yield a similar breakup period at the same feed speed.

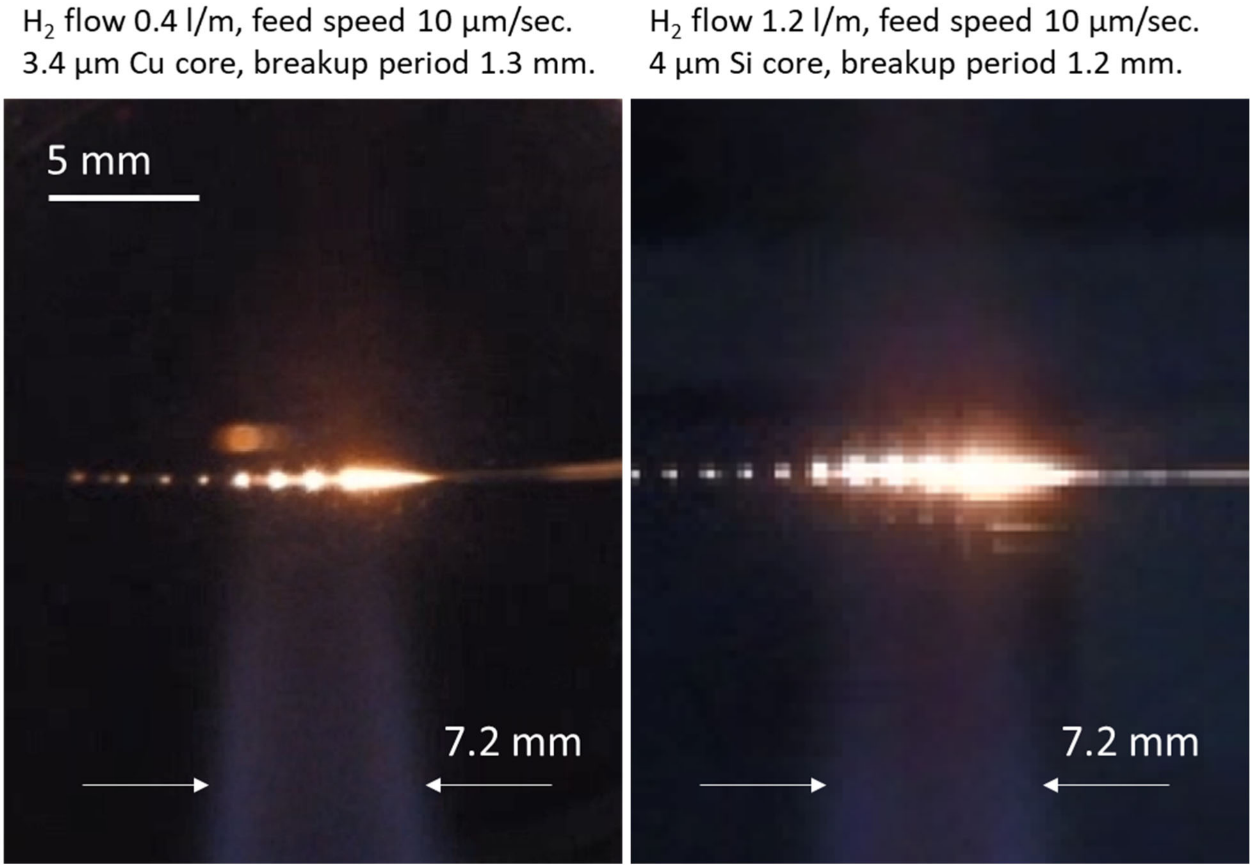

Supplementary Fig. 15. **Heuristic comparison of the flame resulting in the hydrogen flow of 0.4 l/m vs. 1.2 l/m.** The flame height grows with the increase in the gas flow rate. Yet, the fiber position in the flame for every consecutive breakup experiment is adjusted to stay at the flame tip. Thus, the hot zone width and its geometry are roughly preserved. The heat deposition and, thus, the maximal temperature of the hot zone increases with the gas flow rate, resulting in increased emissivity of the core material.

Supplementary References

1. Powers, T. R., Zhang, D., Goldstein, R. E. & Stone, H. A. Propagation of a topological transition: The Rayleigh instability. *Physics of Fluids* **10**, 1052–1057 (1998).
2. Zhang, J. *et al.* Laser-Induced In-Fiber Fluid Dynamical Instabilities for Precise and Scalable Fabrication of Spherical Particles. *Adv Funct Mater* **27**, 1703245 1–8 (2017).
3. Fujii, H., Matsumoto, T., Izutani, S., Kiguchi, S. & Nogi, K. Surface tension of molten silicon measured by microgravity oscillating drop method and improved sessile drop method. *Acta Mater* **54**, 1221–1225 (2006).
4. Lucas, L. D. *Techniques de l'Ingénieur*. (1984).
5. PARIKH, N. M. Effect of Atmosphere on Surface Tension of Glass. *Journal of the American Ceramic Society* **41**, 18–22 (1958).
6. Li, J. G. & Hausner, H. Wetting and adhesion in liquid silicon/ceramic systems. *Mater Lett* **14**, 329–332 (1992).
7. Eaglesham, D. J., White, A. E., Feldman, L. C., Moriya, N. & Jacobson, D. C. Equilibrium shape of Si. *Phys Rev Lett* **70**, 1643–1646 (1993).
8. Danielson, D. T., Sparacin, D. K., Michel, J. & Kimerling, L. C. Surface-energy-driven dewetting theory of silicon-on-insulator agglomeration. *J Appl Phys* **100**, 83507 1–10 (2006).
9. COMSOL AB. COMSOL Multiphysics. www.comsol.com.
10. Faccini de Lima, C. *et al.* Towards Digital Manufacturing of Smart Multimaterial Fibers. *Nanoscale Res Lett* **14**, 209 1–16 (2019).
11. Petropoulou, A., Drikakis, D. & Riziotis, C. Microspheres Formation in a Glass–Metal Hybrid Fiber System: Application in Optical Microwires. *Materials 2019, Vol. 12, Page 1969* **12**, 1969 1–12 (2019).
12. Gumennik, A. *et al.* Silicon-in-silica spheres via axial thermal gradient in-fibre capillary instabilities. *Nat Commun* **4**, 2216 1–8 (2013).
13. Gumennik, A. *et al.* Confined in-fiber solidification and structural control of silicon and silicon−germanium microparticles. *PNAS* **114**, 7240–7245 (2017).
14. Grodkiewicz, W. H. Fused silica fibers with metal cores. *Mater Res Bull* **10**, 1085–1090 (1975).
15. Zhang, T. *et al.* High-performance, flexible, and ultralong crystalline thermoelectric fibers. *Nano Energy* **41**, 35–42 (2017).
16. Scott, B. L. & Pickrell, G. R. Fabrication of GaSb Optical Fibers. in *Processing and Properties of Advanced Ceramics and Composites V* vol. 240 65–70 (wiley, 2013).

17. Ballato, J. & Snitzer, E. Fabrication of fibers with high rare-earth concentrations for Faraday isolator applications. *Applied Optics, Vol. 34, Issue 30, pp. 6848-6854* **34**, 6848–6854 (1995).

18. Ballato, J. *et al.* Binary III-V semiconductor core optical fiber. *Opt Express* **18**, 4972–4979 (2010).

19. Homa, D., Liang, Y. & Pickrell, G. Superconducting fiber. *Appl Phys Lett* **103**, 082601 1–4 (2013).

20. Homa, D., Kaur, G., Pickrell, G., Scott, B. & Hill, C. Electronic and magnetic fibers. *Mater Lett* **133**, 135–138 (2014).

21. Kaufman, J. J. *et al.* Structured spheres generated by an in-fibre fluid instability. *Nature* **487**, 463–467 (2012).

22. Rein, M. *et al.* Self-assembled fibre optoelectronics with discrete translational symmetry. *Nat Commun* **7**, 12807 1–8 (2016).

23. van der Elst, L. A. *et al.* Microstructured Electroceutical Fiber-Device for Inhibition of Bacterial Proliferation in Wounds. *Adv Mater Interfaces* **10**, 2201854 1–11 (2023).
