# Supplementary material for: Multimaterial fiber as a physical simulator of a capillary instability
Source: Nat Commun. 2023 Sep 26;14:5816. doi: 10.1038/s41467-023-41216-7 (PMC10522671; doi:10.1038/s41467-023-41216-7)
Supplement: Supplementary file 3 — Description of Additional Supplementary Files [file 41467_2023_41216_MOESM3_ESM.pdf]

<u>Description of Additional Supplementary Files</u>

Supplementary Movie 1 – representative breakup simulation ab-initio.

Supplementary Movie 2 – experimental breakup of 4-micron Si-core 280-micron cladding silica fiber at feed speed 10, 30, and 50 microns/sec (framerate accelerated x 32).
