# Supplementary material for: Multimaterial fiber as a physical simulator of a capillary instability
Source: Nat Commun. 2023 Sep 26;14:5816. doi: 10.1038/s41467-023-41216-7 (PMC10522671; doi:10.1038/s41467-023-41216-7)
Supplement: Supplementary file 2 — Peer Review File [file 41467_2023_41216_MOESM2_ESM.pdf]

nature portfolio

Peer Review File

Multimaterial Fiber as a Physical Simulator of a Capillary Instability

Open Access This file is licensed under a Creative Commons Attribution 4.0 International License, which permits use, sharing, adaptation, distribution and reproduction in any medium or format, as long as you give appropriate credit to the original author(s) and the source, provide a link to the Creative Commons license, and indicate if changes were made. In the cases where the authors are anonymous, such as is the case for the reports of anonymous peer reviewers, author attribution should be to 'Anonymous Referee' followed by a clear attribution to the source work. The images or other third party material in this file are included in the article's Creative Commons license, unless indicated otherwise in a credit line to the material. If material is not included in the article's Creative Commons license and your intended use is not permitted by statutory regulation or exceeds the permitted use, you will need to obtain permission directly from the copyright holder. To view a copy of this license, visit http://creativecommons.org/licenses/by/4.0/.

REVIEWER COMMENTS

Reviewer #1 (Remarks to the Author):

The paper reports on a fluid dynamics model to simulate the capillary breakup inside multimaterial fibres. I am not an expert in fluid dynamics modelling and thus cannot comment on the model itself. I have a few concerns about the conclusion and presentation of the paper:

* The papers claims that the multimaterial fibre is a universal physical simulator of capillary instability. However, the model was only verified for one system: silicon-in-silica fibre. More systems should be used to show the universal nature.

* Figure 2 is an example that the figure caption is no sufficient to describe what is shown in the figure. The different colours are not explained. For example, in figure 2c, it is not clear what the light magenta 'rod' stands for. Is it at a different time scale, i.e. that the rod is formed via stretching and then after a certain time, it turns into a red sphere-like shape due to surface tension? Or does it show two scenarios with different material properties considered?

* It should be clearer articulated under which conditions the capillary breakup becomes predictable. The abstract explains it quite good, but in the main text it is lost in the details. I find the explanation of the model hard to follow for a non-expert.

Reviewer #2 (Remarks to the Author):

Authors study capillaries instabilities and break up phenomena in multimaterial fibers, which is a promising platform for the development of in-fiber integrated optoelectronic components and devices. The work proposes, develops, and formulate a model based on Axial Gradient Instability model (so called AVG-IM in the manuscript) based on a physically intuitive geometric approach of break up formation in a multimaterial fiber system. It is assumed that by direct heat or laser based local heating the fiber reliquefies locally, and capillary instability breaks up its initially continuous cylindrical cores into arrays of discrete spheres. It is proved that the model derivation is essentially equivalent to Euler Lagrange dynamics. Current physically intuitive models such as Tomotika and Plateau-Rayleigh, both fail to accurately predict the observed breakup periods in real capillary systems while the proposed model is

more accurate. Break up wavelength and breakup location are predicted by AVG-IM in good agreement with experimental results and also to ab initio Navier-Stokes models. However, ab-initio simulations are computationally intensive and impractical for certain physical systems.

The model has some reasonable validity limitations related to the practical implementation of instability studies in a multimaterial fiber exposed to thermal processing while feeded with material during the drawing process and these are: 1) the width of the hot-zone boundary is finite, and 2) the feed speed is finite.

The work is significant as it provides for the first time an intuitive physical understanding and formulation of the computational problem of capillary instability that could allow the deterministic design of features such as microspheres. Current accurate ab initio methods are extremely computational intensive and impractical for application in real problems. Although the results of instability studies is focused in a rather restricted audience working on fiber based capillary instabilities, the physical approach is well formulated and pedagogical and has a broader value suitable for publication in Nature.

The description of work is complete supported by carefully designed experimental studies based on prototype composite fibers of silica claddings and silicon core. The results within the control range are in excellent agreement with the theoretical predictions. Furthermore ab-initio simulations based on Navier-Stokes formulation agree with the proposed AVG-IM method further validating the results.

The followed methodology of the problem definition, formulation and development of theoretical model is well defined and scientifically accurate, while the work has been described in adequate detail in both the theoretical and the experimental validation part.

Further to the aforementioned discussion, a few issues need to be considered by the Authors:

1) The study could be supported also on the validation part with complete simulation of the capillary break up phenomenon by FEM (COMSOL) simulation, which is reasonably achievable in such a well-defined example of constant diameter silica/silicon fiber, considered here. This will provide an additional comparison with the model and the ab-initio simulation.

2) The title would be better described as: “Multimaterial Fiber as a Physical Simulator of a Capillary Instability”

3) The papers claims the model application in multimaterial fibers , although the development of the model was restricted to the specific case of a 2-material systems and validated in a silica /silicon composite fiber system. I suggest that authors should discuss briefly and comment the generalization aspects in greater than two (2) material system or multiple cores. Obviously the application of model depends on the distance and properties of cores/capillaries. Can be applied a linear superposition of individual capillary phenomena in distant and adequately separated core fibers? What if individual cores are in adequate proximity and heating zone affects both simultaneously?

4) Is understandable that Authors considered the simplified case of uniform cladding diameter during the drawing process. However there is a significant interest in flexible post processing of fibers through tapering by using heating and stretching method (for example: Materials 2019, 12, 1969; doi:10.3390/ma12121969) that can create through breakup, variable microsphere sizes in contrast to the method here. While appreciating the theoretical model focus, assumptions and the considered case, could authors comment on the potential applicability or adaptability of the method and the associated potential implications in such a more complex case of a linear diameter tapering?

5) At some sentences the word “Multimaterial” has been replaced probably by mistake by the word “Multilateral”

Reviewer #3 (Remarks to the Author):

This paper reports on the development of an axial viscosity gradient instability model (AVG-IM) that can be used to provide useful physical insight into the breakup of a multi-material fiber core. The motivation for this work is that capillary instability and the resulting breakup of a cylindrical core into a period array of spheres is a useful way to produce in-fiber optoelectronic devices. Thus, understanding the breakup so that it can be controlled is necessary to ensure that devices can be produced with spheres of the desired number, dimensions, and period. As such, the results are interesting and show good agreement with the silicon core fibers under test, and thus these are likely to be of interest to the silicon photonics and optoelectronics community. However, I have to say that I have found interpreting the results quite challenging (though I admit I am not an expert on fluid dynamics) and I do have some questions around the general usefulness of the studies and how they could be more broadly applied. In this regard, I have provided a series of comments that I feel would be helpful if the authors could address during their revisions.

1. The authors present the analysis in a very general way, that is not necessarily specific to a particular core/cladding material combination. It would be helpful to know what the optimum range is for the viscosity ratio between the core/cladding, and thus what range of fiber materials this could be applicable too.

2. The authors state that AVG breakup cannot be thought of as either dripping or jetting, does this mean that they are looking for conditions of Ca~1? If so, how is this controlled/ensured?

3. The temperature profile of the hot zone is assumed to be an exponential function. What sort of heating is the relevant to? Is it just for flame heating or could it work for a laser as well? What happens if you use a different temperature profile? Does everything else still hold?

4. I find the presentation of the results in Fig 4 very confusing. As these seem to be the main results, it makes understanding the conclusions for this work challenging. For example, there are no y-axis labels. Do the titles on the top of the columns correspond to the y-axis? Also, the authors talk about the model fit and ab-initio calculations. It would be helpful if the model fit could be more clearly defined. I presume this is their AVG-IM model – if so, could they just call it that? Both the labels and the caption should be improved.

Also, the model seems to fit well with the experimental data for all values of beta but the ab-initio does not. Which is correct?

Moreover, in the text they state:

"For this reason, we consider that the breakup period data from the simulations may be trusted qualitatively, while the breakup location data can be trusted quantitatively."

Is this just for the ab-initio simulations? If so, why does their model work so much better? Some discussion around this would be very helpful.

5. Related to the point above, on line 252 the authors state that they get good quantitative agreement for beta ~60 for the experiments, model, and the breakup positions for the ab-initio simulations (shown both in Fig 5 and Fig 6). However, other values of beta give good agreement between the model and the experiments in Fig 5. Why is this? Is this expected and how could you arrive at the right beta without comparison to the ab-initio simulations?

6. The authors conclude that multi-material fibers can be used as a generalized physical simulator of fluid dynamic processes. Is this true for all multi-material fibers, or just the silicon core/silica cladding fibers?

7. Finally, it would be helpful if the authors could comment on what useful applications could be derived from this work.

Minor points

1. On line 114 the font used for the definition of the core radius is different from what appears in the equations. Are these the same thing?

2.In the sentence starting on line 102, the position x_bu appears twice, but it seems the two positions should be different. Is this correct or should one x_bu be something else?

3. On line 212, a previous study is mentioned in relation to core emissivity. They claim this was for a germanium core fiber, but this was actually a SiGe core fiber I believe.

4. In the SI, line 23, "α'" is mentioned but not defined. Is this related to the core radius a? (Note, I am referring to the use of different fonts again.) "α" also appears in several equations and so should be clearly defined.

5. SI Fig S3. There are no labels on the axis. Although they are mentioned in the caption, it is quite confusing.

6. SI Fig. S7, the labels in the legend do not match the caption. There are also typos in the labels and caption. I also do not understand the data in this. It is claimed that the BR3 data is obtained much faster than the jdj data, but yet the simulated time for the BR3 data is longer (though there are no units so I can't be sure).

First, we would like to thank the reviewers for their valuable comments. The comments were quite insightful and gave us a clear direction for improving our manuscript, such that it addresses all possible reader curiosities and concerns. Due to that detailed and focused direction, we improved our product significantly.

It looked like a consensus among the reviewers that the paper, as first submitted, lacked a validation of AVG-IM in the broader range of parameters relevant to the fiber and breakup process.

It looked like a consensus, as well, that the experimental validation, demonstrating the technological relevance of the model to engineering devices and systems in fibers, is necessary to convince the reader that the model is indeed a helpful tool.

For that reason, we added a new section to the paper "Discussion II: Assessment of AVG-IM General Applicability Limits. Examples of Practical Uses of AVG-IM in Fiber-Device Engineering". It demonstrates, corroborated by ab-initio, that AVG-IM extrapolates broadly to various fiber geometries, compositions, and breakup conditions. It also investigates practical device-assembly designs in multimaterial and multicore fibers, suggesting "educated guesses" for device assembly scenarios based on AVG-IM analysis of experimental breakup results and demonstrating their implementation. It discusses in detail which conditions the model doesn't apply to and suggests pathways to its generalization wherever possible.

Since the revised paper is a significantly rewritten version of the original, we have highlighted only the portions of the main text that directly address the reviewers' points rather than all edited/added text.

REVIEWER COMMENTS ADDRESSED POINT BY POINT

Reviewer #1:

* The papers claims that the multimaterial fibre is a universal physical simulator of capillary instability. However, the model was only verified for one system: silicon-in-silica fibre. More systems should be used to show the universal nature.

We have added in Discussion II, Figure 8, a study of the capillary breakup of copper that includes analysis of it with AVG-IM in comparison to Si, which allows deriving technologically and scientifically relevant conclusions, such as proposing a scenario to an assembly of Cu-Si Schottky diodes in fibers and demonstrating a novel method of measuring interface tension between two melts based in AVG-IM as the analysis framework (also, in Methods, section "Comparison of the methods for measuring the interface energy: sessile-drop method vs. fiber-AVG-IM method")

* Figure 2 is an example that the figure caption is no sufficient to describe what is shown in the figure. The different colours are not explained. For example, in figure 2c, it is not clear what the light magenta 'rod' stands for. Is it at a different time scale, i.e. that the rod is formed via stretching and then after a certain time, it turns into a red sphere-like shape due to surface tension? Or does it show two scenarios with different material properties considered?

We added a clarifying explanation to the caption of Figure 2: “The core section reshaping into a growing droplet of melt at the core tip is overlayed over a fainted image of the original core, how it would look at a given time if it wouldn’t reshape. It demonstrates the time-lapse of the growing core-section length streamed into the growing droplet as the capillary instability develops between the pinch events.

* It should be clearer articulated under which conditions the capillary breakup becomes predictable. The abstract explains it quite good, but in the main text it is lost in the details. I find the explanation of the model hard to follow for a non-expert.

In the Results section, **page 7**, we have edited the explanation that leads to the formulation of the central set of equations of the model, Eq. (2), and we hope it reads easier now.

In the Results section, the last paragraph of it on **page 9**, we have rewritten for more clarity the definition of the validity limitations VL1 and VL2 for the AVG-IM, i.e., that the model is valid for the regime in which the breakup wavelength is comparable to the width of the liquefaction zone boundary. The pinch-off position falls within that boundary. Further clarifications are in the Methods section, “Assertation of the validity limitations of the AVG-IM.” In Discussion II we have added an experimental examination of the validity limitations of AVG-IM in single- and double-core Si fibers, in explanation to Figures 8 and 9, respectively.

Reviewer #2:

1) The study could be supported also on the validation part with complete simulation of the capillary break up phenomenon by FEM (COMSOL) simulation, which is reasonably achievable in such a well-defined example of constant diameter silica/silicon fiber, considered here. This will provide an additional comparison with the model and the ab-initio simulation.

In parallel to the ab-initio simulations described in the paper, we have been conducting Comsol studies of the same. We have been able to simulate a stationary fiber in a stationary heat source reliably.

Yet, we haven’t found a viable way to reliably simulate a geometry that includes a movement of the heat source along the fiber axis, either moving the fiber while the heat source is stationary or vice versa. All those attempts led to results that didn’t look physical.

For instance, the cladding is highly viscous at any temperature. When moving the fiber through the heat source, we would observe turbulences in some of those simulations, which are only possible in inviscid liquid. At the same time, that turbulence wouldn’t occur in an exactly identical stationary configuration.

Comsol, however powerful it is, is a “black box,” which we had no success in hacking to do what we needed, even with the extensive support of the Comsol engineering team. Since we had no positive results after a years-long effort, we decided to leave it for now and rely on our software tailored to solve fluid dynamics problems.

We have a source code for the ab-initio Navier-Stokes equations simulations done in this study. We know what it does exactly. We are convinced that it provides reliable, physically meaningful results, and since we have recently installed it on Big Red 200, IU’s newest supercomputer, it gives those results relatively fast.

At the end of this text, we have appended a section, “Considerations on COMSOL simulations of AVG-Breakup, “ detailing our thought process and results in addressing breakup in fibers with Comsol over the years.

2) The title would be better described as: “Multimaterial Fiber as a Physical Simulator of a Capillary Instability”

The title has been edited according to this suggestion.

3) The papers claims the model application in multimaterial fibers , although the development of the model was restricted to the specific case of a 2-material systems and validated in a silica /silicon composite fiber system. I suggest that authors should discuss briefly and comment the generalization aspects in greater than two (2) material system or multiple cores. Obviously the application of model depends on the distance and properties of cores/capillaries. Can be applied a linear superposition of individual capillary phenomena in distant and adequately separated core fibers? What if individual cores are in adequate proximity and heating zone affects both simultaneously?

In Discussion II, Figure 8, an investigation of Cu-core breakup is compared to Si-core breakup, including a discussion on similar instability behaviors between such dissimilar materials when exposed to dissimilar heat sources and dissimilar instability behavior when exposed to the same heat source.

In Figure 9, a double-core Si fiber is investigated, in which the cores are closely packed. Using AVG-IM analysis, we find that the breakups are coupled mechanically in some conditions and decoupled in others. We discuss the implications of that fact to the breakup-induced device self-assembly in multicore-fibers. We conclude that the distance between the cores affects the process. In closely packed cores, i.e., when the sum of the sphere radii pinching from the neighboring cores is larger than the separation between the original, continuous cores, the breakup cannot be treated as a superposition of individual core behaviors.

4) Is understandable that Authors considered the simplified case of uniform cladding diameter during the drawing process. However there is a significant interest in flexible post processing of fibers through tapering by using heating and stretching method (for example: Materials 2019, 12, 1969; doi:10.3390/ma12121969) that can create through breakup, variable microsphere sizes in contrast to the method here. While appreciating the theoretical model focus, assumptions and the considered case, could authors comment on the potential applicability or adaptability of the method and the associated potential implications in such a more complex case of a linear diameter tapering?

In the beginning of Discussion II, Simplification S1 (**page 13**), and further on in the Supplementary SI.IX, we cite the paper mentioned in this comment. After reading it carefully, we experimentally investigate the influence of varying core and cladding thickness on the applicability of AVG-IM to adiabatic tapers. Further, in Simplification S1, we suggest a pathway to generalizing the model for non-adiabatically tapered fibers with a varying core diameter.

5) At some sentences the word “Multimaterial” has been replaced probably by mistake by the word

“Multilateral”

Thanks, it has been fixed. We also proofread the article multiple times by all the authors.

Reviewer #3:

1. The authors present the analysis in a very general way, that is not necessarily specific to a particular core/cladding material combination. It would be helpful to know what the optimum range is for the viscosity ratio between the core/cladding, and thus what range of fiber materials this could be applicable too.

In Discussion II, page 13, in Simplification S2, we talk about the range of the materials to which AVG-IM applies as is, for all of which $\eta_{\text{core}}/\eta_{\text{clad}}$ is negligible. Those include semiconducting, metallic, magnetic, optically nonlinear, superconducting, thermoelectric, ferroelectric, and piezoelectric [1–10] cores in a silica cladding. It also suggests a pathway to generalizing the model for finite $\eta_{\text{core}}/\eta_{\text{clad}}$, such as in chalcogenide-core polymeric fibers.

One way to emulate dissimilar core-cladding combinations to which AVG-IM applies is through core-cladding interface tension γ.

Typically, when the core and cladding are two chemically dissimilar glasses or ceramics, the values of γ are of the order of 0.1 N/m. In contrast, if the cladding is glass and the core is metal, the typical value of γ is ≥1 N/m. For semiconducting-core silica glass fibers, γ falls in between those two extremes.

Figure 7D demonstrated the extrapolation of AVG-IM to a range of γ’s corroborated against ab-initio simulations.

Figure 8 demonstrates the applicability of AVG-IM to material systems with very different interface energies, on the example of Si-silica and Cu-silica, and discusses the technological implications of it to the in-fiber device assembly.

2. The authors state that AVG breakup cannot be thought of as either dripping or jetting, does this mean that they are looking for conditions of Ca~1? If so, how is this controlled/ensured?

The main point that this paper makes is that the predictable breakup process itself, in conditions of axial viscosity gradient, “chooses” a steady-state pinch-off location at the conditions, where

$$\left\{\begin{array}{c} \mathrm{Ca}(\lambda,x)\big|_{x_{bu}} = 1 \\ \left(\frac{d}{dx}\mathrm{Ca}(\lambda,x)\right)\Big|_{x_{bu}} = 0 \end{array}\right\} = \left\{\begin{array}{c} \left.\frac{\beta a\,\eta_{clad}(x)\cdot v_f}{\gamma\lambda}\right|_{x_{bu}} = 1 \quad (2a) \\ \left(\frac{\partial}{\partial x}-\frac{\partial}{\partial\lambda}\right)\left(\frac{\beta a\,\eta_{clad}(x)\cdot v_f}{\gamma\lambda}\right)\Big|_{x_{bu}} = 0 \quad (2b) \end{array}\right\}$$

Equation (2a) is Ca=1. Since the viscosity gradient is a function of the axial position of the fiber in the flame, the fiber that is fed into the flame will assume this position necessarily, at some point, for every lambda. Equation (2a) formulates the condition for which the instability will develop into the pinch-off at the earliest. One can think of it as energy conservation in a process where potential energy on the core-cladding interface is translated into work of overcoming viscose forces. The position that ensures

that this conversion happens as fast as possible is the pinch-off location. When the droplet starts growing, such conversion starts; when it pinches, it is completed. It also can be thought of as synchronization of the timing dedicated to the growth of the droplet at the core tip with the timing necessary for its pinching. And the location of the pinching ensures the fastest synchronization of that kind.

For better clarity, we have edited the model explanation on Page 7, which results in Eq. (2) formulation according to the physical picture of timing synchronization.

The discussion on Page 8 deals with the same in terms of energy conservation.

3. The temperature profile of the hot zone is assumed to be an exponential function. What sort of heating is the relevant to? Is it just for flame heating or could it work for a laser as well? What happens if you use a different temperature profile? Does everything else still hold?

Exponentials form a complete set, meaning that any function can be decomposed in exponentials (in the same way as any continuous and differentiable function can be decomposed in polynomials).

The first-order decomposition of a continuous differentiable function is a single exponent. For a hot zone boundary resulting from a single-orifice flame, or a low-order laser beam, such as Gaussian, which is a typical zero-order laser mode, or a Bessel function, which is a basic mode emerging from a fiber, it would describe reasonably well the temperature profile in its fiber-entrance boundary, i.e., the region relevant to the predictable kind of the breakup. This is because, qualitatively, at any entrance to any of such hot zones, the temperature will be rising from the melting point of silicon to the maximal temperature in the middle of the hot zone monotonously and with a monotonously decreasing temperature gradient towards the hot zone center, likewise the single exponent.

In Discussion II Figure 7C, we test AVG-IM for validity for the next order decomposition - a double-exponential profile. We further demonstrate the pinch-off temperature region is limited, such that the approximate decomposition of the real-world profile in exponential, practically speaking, should be accurate only in a relatively small window of ~200 K for AVG-IM to correspond accurately to the real-world behavior of the system.

The best sanity check is always the fit to the experimental results. Figures 5, 6, 8, 9, and S11 all show the model-experiment correspondence in a variety of core materials, core dimensions, cladding dimensions, feed speeds, and heat-source maximal temperatures and overall profiles resulting from flames of varying overall flow rate and relative hydrogen-oxygen ratio. Of course, one needs to verify this experimentally to ensure it works well for lasers. But so far, we have no reason to think that it won't, at least as long as the profile is a single-peak function of some kind, such as Gaussian.

4. I find the presentation of the results in Fig 4 very confusing. As these seem to be the main results, it makes understanding the conclusions for this work challenging. For example, there are no y-axis labels. Do the titles on the top of the columns correspond to the y-axis? Also, the authors talk about the model fit and ab-initio calculations. It would be helpful if the model fit could be more clearly defined. I presume this is their AVG-IM model – if so, could they just call it that? Both the labels and the caption should be improved.

In Figure 4, titles on the top of each column correspond to the y-axis of all the graphs in the respective column; that format was chosen to avoid redundancy, which would clutter the figure. The titles on the left show the beta choice for the respective row.

In the analysis in Figure 4, we look for the beta and temperature profile for which the pinch-off location coincides for the ab-initio, AVG-IM, and experiments. For those same conditions, we require that the breakup period for the model coincides with that found in experiments but not necessarily with ab-initio.

On **page 11,** we explain that since ab-initio calculations use the level-set method implemented[11,] which doesn't conserve volume, we can't rely on the breakup period simulated ab-initio quantitatively, only qualitatively.

In simulations, as the capillary instability develops, the lack of mass conservation results in the core thinning out in addition to pinching, causing the pinch process to happen faster, at a higher rate, than in real-world conditions, where volume is conserved. In other words, the simulated period is expected to undershoot the experimental results.

On the other hand, the ab-initio simulated breakup location is trusted quantitatively. Because to even start reshaping, the core needs to reach the right viscosity region. We have also verified this rigorously in Discussion II Figure 7C, obtaining that, looking at the simulation results, the pinch-off location is determined by the absolute temperature at the pinch-off location only, with no regard to the overall shape of the profile.

Also, the model seems to fit well with the experimental data for all values of beta but the ab-initio does not. Which is correct?

Ab-initio has only one input – the temperature profile.

The model has two inputs before the beta is set – the temperature profile and beta. For every beta, one can find a temperature profile that will fit the model well to the experimental results. Thus, the only way to obtain the correct beta for the model is to check for which beta the pinch-off location the model provides coincides with the pinch-off location the ab-initio calculations provide for the same temperature profile, which is what is highlighted by the red dashed-line rectangle as the correct beta range for the model in Figure 4.

Moreover, in the text they state:
"For this reason, we consider that the breakup period data from the simulations may be trusted qualitatively, while the breakup location data can be trusted quantitatively."
Is this just for the ab-initio simulations? If so, why does their model work so much better? Some discussion around this would be very helpful.

We don't trust the breakup period of the ab-initio because it doesn't conserve volume due to the use of the level-set method, as explained above.

The model doesn't have any approximations of that or any other kind that wouldn't make the breakup period provided by it non-trustable. The best sanity check is always the experiment

On the one hand, it was impossible to find a temperature profile that would make ab-initio fit well the entire experimental data set (i.e., the period and the pinch-off location) concurrently, only each subset individually, i.e., breakup period XOR pinch-off location. Thus, we had to choose which one of the subsets provided by the simulation to trust. We have chosen to trust the pinch-off location for the lack of volume conservation in the simulation.

But on the other hand, the model fits the experiment for the entire set of experimental data, both period and pinch-off location. In that sense, we think that AVG-IM is more accurate in representing the whole picture than ab-initio.

5. Related to the point above, on line 252 the authors state that they get good quantitative agreement for beta ~60 for the experiments, model, and the breakup positions for the ab-initio simulations (shown both in Fig 5 and Fig 6). However, other values of beta give good agreement between the model and the experiments in Fig 5. Why is this? Is this expected and how could you arrive at the right beta without comparison to the ab-initio simulations?

For Figure 5 $\beta = 60$, and the feed speed is scanned. Figure 5 is a graphical representation of Eq (2), the solution of which is the model fit in Figure 6. Panel 2A. represents Eq. (2a), Panel 2B. represesets Eq. (2b). We have edited the caption of Figure 5 to add this clarification. For Figure 6 $\beta = 60 \pm 10$ to also consider a reasonable error range because in Figure 4, we found that beta fits the model to ab-initio and experiments well everywhere in the range of betas from 50 to 70.

6. The authors conclude that multi-material fibers can be used as a generalized physical simulator of fluid dynamic processes. Is this true for all multi-material fibers, or just the silicon core/silica cladding fibers?

In the new Discussion II section, we have further verified the model for Si- and Cu-cores in Figure 8.

Figure 7D demonstrated the extrapolation of AVG-IM to a range of γ's corroborated against ab-initio simulations. Scanning γ emulates a choice of different core materials, as has already been mentioned above.

7. Finally, it would be helpful if the authors could comment on what useful applications could be derived from this work.

Based on AVG-IM analysis of experimental results, we have come up in Figure 8 with an in-fiber "staggered" device-assembly scenario in dissimilar core materials, such as Cu-Si Schottky diodes. We also have demonstrated a novel way of measuring interface energy between two melts, using Si-core fiber as a reference and AVG-IM as an analysis framework. In Figure 9, we demonstrated and analyzed using AVG-IM the "interference" in-fiber device assembly in similar core materials, such as pn-Si diodes, and have drawn conclusions of technological relevance, such as whether the breakups in neighboring cores are coupled or they are a superposition of independent processes in individual cores, and whether the assembly scenario is miniaturizable to the nanoscale. This is relevant to the scalability of the in-fiber device fabrication.

Minor points

1. On line 114 the font used for the definition of the core radius is different from what appears in the equations. Are these the same thing?

We have proofread and fixed it.

2.In the sentence starting on line 102, the position x_bu appears twice, but it seems the two positions should be different. Is this correct or should one x_bu be something else?

We have proofread and fixed it.

3. On line 212, a previous study is mentioned in relation to core emissivity. They claim this was for a germanium core fiber, but this was actually a SiGe core fiber I believe.

We have proofread and fixed it.

4. In the SI, line 23, "α'" is mentioned but not defined. Is this related to the core radius a? (Note, I am referring to the use of different fonts again.) "α" also appears in several equations and so should be clearly defined.

We have proofread and fixed it.

5. SI Fig S3. There are no labels on the axis. Although they are mentioned in the caption, it is quite confusing.

We have proofread and fixed it.

6. SI Fig. S7, the labels in the legend do not match the caption. There are also typos in the labels and caption. I also do not understand the data in this. It is claimed that the BR3 data is obtained much faster than the jdj data, but yet the simulated time for the BR3 data is longer (though there are no units so I can't be sure).

We have edited the caption to add clarification. This figure shows that simulations ran at BR3 achieved extended simulated times compared to jdj, (i.e., more simulation time units are simulated, a more significant portion of the simulation process is completed) within shorter actual run times of the calculation. More simulated time units in the same real-world time mean faster simulation.

The rest was proofread and fixed.

References:

1. Gumennik, A. *et al.* Silicon-in-silica spheres via axial thermal gradient in-fibre capillary instabilities. *Nat Commun* **4**, 2216 (2013).
2. Gumennik, A. *et al.* Confined in-fiber solidification and structural control of silicon and silicon−germanium microparticles. *PNAS* **114**, 7240–7245 (2017).
3. Zhang, J. *et al.* Laser-Induced In-Fiber Fluid Dynamical Instabilities for Precise and Scalable Fabrication of Spherical Particles. *Adv Funct Mater* **27**, 1703245 (2017).
4. Grodkiewicz, W. H. Fused silica fibers with metal cores. *Mater Res Bull* **10**, 1085–1090 (1975).
5. Homa, D., Liang, Y. & Pickrell, G. Superconducting fiber. *Appl Phys Lett* **103**, 82601 (2013).
6. Homa, D., Kaur, G., Pickrell, G., Scott, B. & Hill, C. Electronic and magnetic fibers. *Mater Lett* **133**, 135–138 (2014).
7. Zhang, T. *et al.* High-performance, flexible, and ultralong crystalline thermoelectric fibers. *Nano Energy* **41**, 35–42 (2017).
8. Snitzer ; Cunnell, E., Saker, E. & Edmond, J. *Fabrication of Fibers with High Rare-Earth Concentrations for Faraday Isolator Applications*. *Opt. Quantum Electron* vol. 39 (2007).
9. Scott, B. L. & Pickrell, G. R. Fabrication of GaSb Optical Fibers. in *Processing and Properties of Advanced Ceramics and Composites V* vol. 240 65–70 (wiley, 2013).
10. Ballato, J. *et al.* Binary III-V semiconductor core optical fiber. *Opt Express* **18**, 4972 (2010).
11. Wang, F. New Modeling of Compact, High-efficiency, and Widely-tunable Gas-phase Terahertz Lasers. (2019).

Considerations on COMSOL simulations of AVG-Breakup:

We have conducted an extensive investigation on the possibility of simulating AVG breakup in fiber cores using COMSOL Multiphysics and worked closely with the software's technical support team in hopes to bypass the challenges faced in simulating such a phenomenon. Among those, the high viscosity contrast and aspect ratio of the system, as well as the inherent non-linearity of the process posed the most difficulty.

Initial approach: stationary fiber

.5As a first-order approach, we began by simulating a stationary regime, that is, we are interested in analyzing how the instabilities first develop in an initially stationary fiber subjected to a thermal gradient. This process is analogous to the initial step of the capillary breakup experiments in Gumennik et al. (2013), where a silica fiber with a 4 µm-thick Si core is fed through the hot zone of a hydrogen-oxygen flame at flows of 0.3 and 0.1 L/min respectively. The results discussed in this section were published in Faccini de Lima et al. (2019).

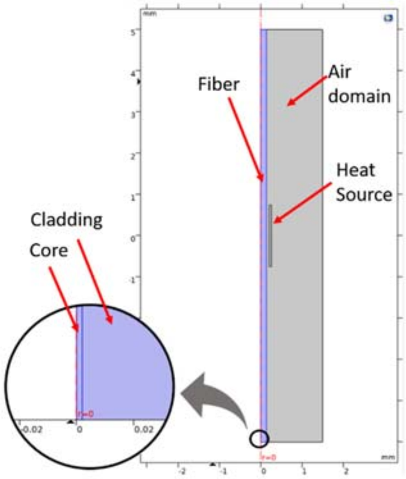

Figure 1: Fiber configuration in the COMSOL simulation

In our simulations (using COMSOL Multiphysics® 5.3a, with its Microfluidics and Heat Transfer modules) we assume an axisymmetric fiber with radius$r_{fiber} = 140\ \mu m$, composed of a thin Silicon core $r_{core} = 2\ \mu m$ enclosed in a Silica cladding. This configuration is shown in Fig. 1, where the Fiber and Air domains are specified, as well as the heat source (located in the air domain) and the fiber's inner structure of cladding and core, in the insert.

The fiber is heated through a heat source located in the air domain, close to the fiber. Parameters such as heat source thickness, length and distance to the fiber were adjusted in order to reproduce the effects of a flame. Here, the heat source is simulated as a cylindrical shell that surrounds the fiber, centered about z=0, with height $h_{heat\ source} = 1.5$ mm and thickness 0.05 mm. Moreover, the center of the heat source is set to be at a distance $r = 1.5 \times r_{fiber}\ mm$ of the fiber surface. The output power P_0 is set to 1.15 W, such that the heat source produces $Q_0 = \frac{P_0}{Volume_{heat\ source}}\ W/m^3$.

Initial heat transfer simulations showed that a fiber length of 15 mm is sufficient to ensure that the fiber extremities remain at room temperature, thus avoiding influence from the boundaries to the breakup process. The external boundaries of the air domain are set to a fixed temperature$T = T_{room} = 293\ K$.

Meshing and other Important Simulation Parameters:

The meshing for the simulations was defined separately for each domain, as described below, and is shown in Figure 2.

- Fiber core: Free triangular mesh, configured for Fluid Dynamics (COMSOL's internal setting), with maximum element size of $5 \times 10^{-4} mm$ and minimum element size of $5 \times 10^{-5} mm$.
- Fiber cladding: Free triangular mesh, configured for Fluid Dynamics (COMSOL's internal setting), with maximum element size of $0.006\ mm$ and minimum element size of $5 \times 10^{-5} mm$.
- Air: Free triangular mesh, configured for General Physics (COMSOL's internal setting), with maximum element size of $0.67\ mm$ and minimum element size of $0.003\ mm$ ("normal" settings).

In addition to the meshing settings discussed above, in this simulation, we used the feature "moving mesh" from COMSOL, in which the free surface is modeled as a geometrical surface separating two domains. Surface tension and other surface forces are directly applied as boundary conditions at the free surface.

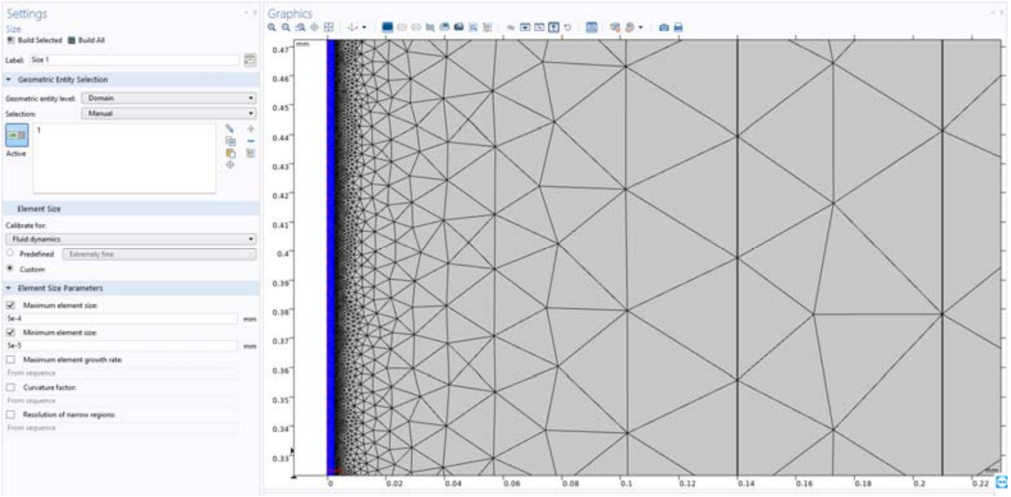

Figure 2:Detail of meshing of the system, with highlight in blue to the configurations of the meshing in the Core domain

Other relevant parameters used in these simulations are listed in Table 1:

Parameter	Expression	Value
Core radius	r_{core}	2 μm
Fiber radius	r_{fiber}	140 μm
Fiber length	h_{fiber}	15 mm
Thermal conductivity of Si	k_{Si}	149 W/mK
Thermal conductivity of SiO_2	k_{SiO_2}	1.3 W/mK
$Si - SiO_2$ Surface tension coefficient	σ_0	1.5 N/m
$Air - SiO_2$ Surface tension coefficient	σ_1	0.75 N/m
Si tangent coefficient of isothermal expansion	α_{Si}	$2.5 \times 10^{-6} K^{-1}$
Softening Temperature of SiO_2	T_s	1710°C
Melting Temperature of Si	T_m	1414°C

Simulation results and discussion:

The simulations are performed in two steps: first, the steady-state temperature profile is calculated for different heat source powers, in order to achieve the desired maximum temperature T_{max}. The results are then exported to a time-dependent fluid-flow simulation, where the Navier-Stokes equation is solved with time steps of 0.05 seconds, from 0 s until the first droplet pinch-off $t_{breakup}$.

The droplet breakup time $t_{breakup}$ is dependent on T_{max}, as can be seen in Figure 3C, where $t_{breakup}$ is plotted for different values of T_{max}. It is possible to observe that $t_{breakup}$ has an exponential dependence on T_{max}, which is expected since $t_{breakup}$ is proportional to the core's viscosity, which in turn is exponentially dependent on the temperature. Preliminary simulations indicate that the phase transition of the Si core has no significant influence on the steady state temperature profile obtained, nor on the subsequent fluid-flow simulations since, in the entire region where the capillary instabilities occur, the temperature is higher than the melting point of Si. Therefore, in order to maintain the model less computationally expensive, we simulate a core of liquid Si as a first approximation. Moreover, although a fully coupled heat-transfer and fluid-flow simulation is preferable, we consider that the effects of coupling bring second-order corrections to the solution and thus can be neglected at this stage. An image of the simulated core

during the first droplet formation, right before the pinch-off, is shown in Figure 3A, for T_{max} =1900°C. A plot of the densities of silica and silicon, as a function of the axial position (in the simulation, the z-axis) for this particular simulation is presented in Figure 3D, with the temperature profile over-imposed.

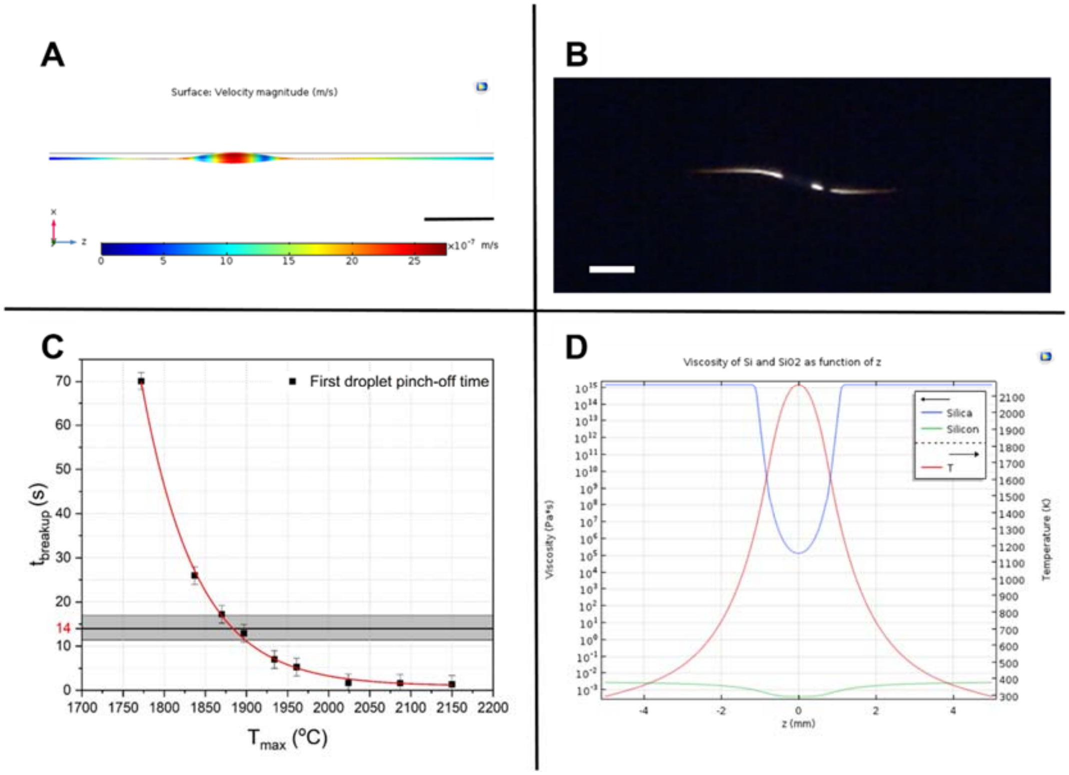

Figure 3: Capillary Breakup Simulations: A} Image of the simulated Si core during the first droplet formation, right before the pinch-off for T_{max}=1900°C. The color scale represents surface velocity (scale bar: 50 μm). B Snapshot of fiber breakup experiment recording just after the first droplet pinch-off, indicating a breakup time of 14 ± 3) s (scale bar: 60 μ). C Breakup time $t_{breakup}$ for different values of T_{max}. The exponential dependence of $t_{breakup}$ on T_{max} is evident, a behavior that is expected due to the dependence of $t_{breakup}$ on the core's viscosity, which in turn depends exponentially on the temperature. The shaded rectangle encompasses the temperature range compatible with this breakup timescale. D Si core and SiO_2 cladding viscosities as a function of axial position, with temperature profile over-imposed, for T_{max}= 1900°C.

Our results allow us to define the ranges of viscosities for which the time scales for breakup are comparable to those observed experimentally, thus assessing qualitatively the temperature profile imposed to the fiber. Figure 3B shows a snapshot of a fiber breakup experiment recorded just after the first droplet pinch-off, at the frame corresponding to t = 39 s. Since the resolution of the images before this frame are not ideal, judging by the symmetry of the breakup behavior it is possible to assess that the pinch-off of the first droplet occurs at t = (35 ±3) s, which translates to a breakup time, after the temperature steady-state regime is achieved, of (14 ±3) s. The shaded rectangle in Figure 3C encompasses the temperature range compatible with this breakup time-scale, from which we can infer that the maximum temperature to which the fiber is exposed is (1885 ± 15)°C.

Although the use of COMSOL's Microfluidics module with Moving Mesh yielded satisfactory results for the case of a stationary fiber, this model is incapable of simulating the system after the droplet breakup - restricting our study to the stages of initial droplet formation. Other simulation approaches with COMSOL included utilizing the Two-Phase Flow module, with Level Set. This approach, although better suited for discontinuous domains, was unsuccessful due to the very high viscosity contrast between the Si core and silica cladding, which could not be handled by the simulation.

REVIEWERS' COMMENTS

Reviewer #1 (Remarks to the Author):

The author addressed all comments and hence the manuscript is now recommended for publication.

Reviewer #2 (Remarks to the Author):

After considering the revised version and the replies to all the reviewers' comments I feel that the manuscript has been drastically improved in terms of technical coverage and physical explanations in several unclear issues. Although the full content of the manuscript with a great number of details makes difficult at some points the continuous follow of the main focus, this is understandable, as the paper deals with a quite challenging problem involving various theoretical and simulations approaches connected to experimental results not always clearly related. However, I have no any useful suggestions to improve the presentation of all this technical analysis in a better and more structured way.

The important issue is that this work provides an entirely new physically insightful approach for the study of capillaries instabilities and break up phenomena in multimaterial fiber systems, and the presented results convince for the validity and applicability of the method, which could be of great interest to a large audience working on fluid dynamics but also to photonics applications. Therefore I suggest the acceptance of the manuscript for publication in Nature Communications.

Reviewer #3 (Remarks to the Author):

The authors have tried hard to address all the comments from the reviewers. Some issues have been more clearly dealt with than others, and I still find a few of the discussions in the paper quite confusing, but this may be because fluid dynamics is not my specialty. But I am happy for this to be published. I suggest that the authors proofread the paper carefully again as there are still some issues with missing words and formatting errors.

All the authors thank the reviewers for the thoughtful and thorough revisions. The reviewers' guidance resulted in a significantly improved and focused study. Our remaining comments on the reviewers' input are below (in red).

REVIEWERS' COMMENTS

Reviewer #1 (Remarks to the Author):

The author addressed all comments and hence the manuscript is now recommended for publication.

Thank you for your favorable consideration of our efforts to improve the paper's content.

Reviewer #2 (Remarks to the Author):

After considering the revised version and the replies to all the reviewers' comments I feel that the manuscript has been drastically improved in terms of technical coverage and physical explanations in several unclear issues. Although the full content of the manuscript with a great number of details makes difficult at some points the continuous follow of the main focus, this is understandable, as the paper deals with a quite challenging problem involving various theoretical and simulations approaches connected to experimental results not always clearly related. However, I have no any useful suggestions to improve the presentation of all this technical analysis in a better and more structured way.

The important issue is that this work provides an entirely new physically insightful approach for the study of capillaries instabilities and break up phenomena in multimaterial fiber systems, and the presented results convince for the validity and applicability of the method, which could be of great interest to a large audience working on fluid dynamics but also to photonics applications. Therefore I suggest the acceptance of the manuscript for publication in Nature Communications.

Thanks a lot! Your input is very encouraging. We believe that you are right in anticipating the high impact of this work, and we are immensely grateful for your insightful comments, suggestions, and encouragement throughout the review process.

Reviewer #3 (Remarks to the Author):

The authors have tried hard to address all the comments from the reviewers. Some issues have been more clearly dealt with than others, and I still find a few of the discussions in the paper quite confusing, but this may be because fluid dynamics is not my specialty. But I am happy for this to be published. I suggest that the authors proofread the paper carefully again as there are still some issues with missing words and formatting errors.

We deeply appreciate your insight and will continuously improve in formulating the physical picture more intuitively and comprehensively in our follow-up studies. Capillary instability is indeed a very complex topic, and the challenge of describing it in simple enough words to result in a very intuitive, easily perceivable depiction goes far beyond the rigor of just mathematically accurate formulation.

Additionally, we have extensively proofread and edited the remaining spelling mistakes, stylistic inconsistencies, and formatting bugs.
